# Oligonucleotide mapping via mass spectrometry to enable comprehensive primary structure characterization of an mRNA vaccine against SARS-CoV-2

**DOI:** 10.1038/s41598-023-36193-2

**Published:** 2023-06-03

**Authors:** Brian C. Gau, Andrew W. Dawdy, Hanliu Leah Wang, Bradley Bare, Carlos H. Castaneda, Olga V. Friese, Matthew S. Thompson, Thomas F. Lerch, David J. Cirelli, Jason C. Rouse

**Affiliations:** 1 BioTherapeutics Pharmaceutical Sciences, Pfizer Inc, Chesterfield, MO USA; 2 BioTherapeutics Pharmaceutical Sciences, Pfizer Inc, Andover, MA USA

**Keywords:** Liquid chromatography, Drug development, RNA vaccines, Mass spectrometry, Data processing

## Abstract

Oligonucleotide mapping via liquid chromatography with UV detection coupled to tandem mass spectrometry (LC-UV-MS/MS) was recently developed to support development of Comirnaty, the world’s first commercial mRNA vaccine which immunizes against the SARS-CoV-2 virus. Analogous to peptide mapping of therapeutic protein modalities, oligonucleotide mapping described here provides direct primary structure characterization of mRNA, through enzymatic digestion, accurate mass determinations, and optimized collisionally-induced fragmentation. Sample preparation for oligonucleotide mapping is a rapid, one-pot, one-enzyme digestion. The digest is analyzed via LC-MS/MS with an extended gradient and resulting data analysis employs semi-automated software. In a single method, oligonucleotide mapping readouts include a highly reproducible and completely annotated UV chromatogram with 100% maximum sequence coverage, and a microheterogeneity assessment of 5′ terminus capping and 3′ terminus poly(A)-tail length. Oligonucleotide mapping was pivotal to ensure the quality, safety, and efficacy of mRNA vaccines by providing: confirmation of construct identity and primary structure and assessment of product comparability following manufacturing process changes. More broadly, this technique may be used to directly interrogate the primary structure of RNA molecules in general.

## Introduction

Two messenger RNA (mRNA) vaccines, Comirnaty from Pfizer-BioNTech and Spikevax from Moderna, have been approved by the FDA, EMA, and other regulatory agencies worldwide to combat the coronavirus disease 2019 (COVID-19) pandemic^1,2^. COVID-19 is caused by severe acute respiratory syndrome coronavirus-2 (SARS-CoV-2) infection^3^. mRNA vaccines can be used for immunization, as they contain the genetic instructions for self-translation of the target immunogenic protein^4^. mRNA in approved COVID-19 vaccines encode for the S-2P protein^5^, which differs from the SARS-CoV-2 spike protein by two stabilizing proline mutations that engender a prefusion conformation^6^.mRNA drug substance (DS) is manufactured by in vitro transcription (IVT) using bacteriophage T7 polymerase with N1-methyl pseudouridine forming a nucleoside-modified messenger RNA (modRNA). modRNA DS is subsequently encapsulated with lipid nanoparticles (LNPs) for cellular delivery and protection from degradative forces. Formulated modRNA-LNPs comprise the drug product (DP) that is administered for vaccination.

Drug manufacturers have an unqualified responsibility to characterize and understand the molecular structure, sequence, heterogeneity, and impurities of the final medicines and vaccines they market. The general primary structure of mRNA DS, from the 5′ to the 3′ end, is: 5′ Cap, 5′ untranslated region (UTR), antigen coding region, 3′ UTR, and 3′ polyadenosine tail (poly(A)-tail)^7^ in a single-stranded format. To be translation-competent, mRNA must be capped on its 5′ end^8^ and have a poly(A)-tail with a sufficient number of consecutive adenosine nucleotides^9^. The 5′ cap is often a 5′ terminal N7-methylated guanosine triphosphate connected to the 5′ carbon of the ribose of the next adenosine or guanosine nucleotide, which may also be methoxylated on its 2′ carbon^10^. These 5′ cap and poly(A)-tail attributes also confer stability by protecting the mRNA from cellular degradation processes^11,12^. Integrity of the full-length mRNA primary structure, including the 5′ cap and poly(A)-tail termini, represent critical quality attributes for vaccine DS that are closely monitored to ensure the desired product quality.

Multiple chromatographic and electrophoretic “oligonucleotide mapping” techniques have been developed over the past decades^13–15^. More recently, mass spectrometry (MS)-based mRNA characterization methods utilizing ion pair reversed-phase high performance liquid chromatography coupled to mass spectrometry (IP RP-HPLC-MS) have been developed for in-depth characterization of mRNA vaccines’ 5′ Cap^16^ and 3′-Poly(A)-tail^17^. However, these require purification and dedicated analytical methods for characterization of each terminus. Additional analytical methods utilizing enzymatic digestions and IP RP-HPLC with and without tandem MS (MS/MS) for oligonucleotide sequencing of large RNA have recently been developed^18–22^. Various in solution enzymes^22^, or immobilized RNase T_1_ on magnetic particles, have been assessed for improved sequence coverage. Nakayama et al. developed a LC-MS based mRNA profiling method using stable isotope-labeled standards. Despite the pioneering work on RNA primary structure characterization discussed earlier^18–22^, it is beneficial to the field to have an analytical method that is both comprehensive and simple to employ. An ideal oligonucleotide map fit for straightforward implementation is one capable of directly and efficiently characterizing the entire length of RNA, including 5′ Cap and 3′ terminal heterogeneity, in a one-pot, one-enzyme digest, requiring no stable-isotope-labeled controls. It resolves sequence isomers and produces a fully annotated UV chromatogram with a map of maximum sequence coverage.

Herein, we describe an oligonucleotide mapping method for direct, comprehensive characterization of full-length mRNA primary structure, using Comirnaty as a case study. Our oligonucleotide mapping method employs state-of-the-art ion pair reversed-phase ultrahigh performance liquid chromatography with UV detection (IP-RP-UHPLC-UV) coupled to ultrahigh-resolution electrospray ionization tandem mass spectrometry (ESI MS/MS) to separate and identify the oligonucleotide fragments generated from Ribonuclease T_1_ (RNase T_1_) digestion. Custom-built data analysis tools enable comprehensive semi-automated primary structure readouts: (1) UV chromatogram completely annotated with hundreds of digestion products that represent the specific fingerprint of mRNA primary structure, (2) coverage map showing unique and maximum sequence coverage, (3) 5′ Cap and 3′ terminal microheterogeneity assessment.

The Pfizer-BioNTech COVID-19 vaccine has been launched in 186 markets globally as of July 2022^23^. To facilitate this rollout, a series of structural elucidation and comparability studies using oligonucleotide mapping were completed and filed with worldwide health authorities. This demonstrated that new DS manufacturing sites and increased production scales, introduced to expand capacity and global supply of the vaccine, produced mRNA with comparable product quality to contemporary batches. Here, our oligonucleotide mapping method facilitated complete primary structure assessment of Comirnaty DS in a single method with 100% maximum sequence coverage. Optimized MS/MS fragmentation parameters and specialized software in conjunction with commercial software were essential in distinguishing oligonucleotide sequence isomers for high maximum sequence coverage. Our comprehensive, semi-automated oligonucleotide mapping method was developed to provide full-length mRNA heightened characterization of sequence, terminal forms, and potential modifications. It is a critical component of mRNA primary structure understanding, comparability assessment, and it may be used as an orthogonal DS identity assay for differentiating highly similar mRNA sequences derived from SARS-CoV-2 variants.

## Methods

Oligonucleotide mapping was developed with a representative batch of Comirnaty BNT162b2 Original DS (i.e., the original Pfizer-BioNTech COVID-19 vaccine that encodes for the spike glycoprotein (S) of the SARS-CoV-2 virus, the Wuhan-Hu-1 isolate: GenBank: QHD43416.1) and it has been applied to subsequent Comirnaty BNT162b2 constructs (BNT162b2s04 [Delta] and BNT162b2s05 [Omicron]) and other portfolio mRNA molecules. Fifty micrograms of mRNA DS was digested with 2500 U of RNase T_1_ in a 50 mM Tris(hydroxymethyl)aminomethane (Tris) pH 7.5 buffer with 20 mM Ethylenediaminetetraacetic acid (EDTA) 90 min at 37 °C. The resulting enzymatic fragment solution was spiked with 10× triethylamine (TEA) and 1,1,1,3,3,3-hexafluoro-2-propanol (HFIP) emulsion to give a final v/v concentration of 0.1% TEA 1% HFIP. A 4 µg load was injected and fragments were separated by ion-pair reversed-phase ultrahigh performance liquid chromatography (IP RP-UHPLC) with UV detection at 260 nm using a 1290 Infinity II Bio LC System (Agilent) paired with an ACQUITY Premier Oligonucleotide C18 column: 130 Å, 1.7 µm, 2.1 × 150 mm (Waters). Each mobile phase contained 0.1% TEA and 1% HFIP. The TEA functions as the ion-pairing agent, and the HFIP provides MS-compatible buffering as a volatile weak acid. The gradient progressed from 1 to 17% mobile phase B (50% methanol) in 195 min, then 17–35% B in 60 min, followed by wash and equilibration segments. The flow rate was 0.2 mL/min with a post column split: 50 µL/min to the UV diode array detector, and 150 µL/min to an Orbitrap Eclipse Tribrid Mass Spectrometer (Thermo Fisher Scientific). The on-line electrospray ionization (ESI) MS acquisition was done in negative ion mode with a spray voltage of 2700 V. MS scans were from 400 to 2000 m/z at 120,000 resolving power (RP) at 400 m/z. Tandem mass spectrometry (MS/MS) was accomplished at 30,000 RP by a 17, 21, 25 stepped higher-energy collisional dissociation (HCD) of multiply charged precursor candidates selected by the data dependent acquisition (DDA) algorithm.

BioPharma Finder version 5.0 software (Thermo Fisher Scientific) was used to identify oligonucleotides based on both MS and MS/MS matches to theoretical RNase T_1_ digest products. An MS match required the observed oligonucleotide neutral mass to be within 5 ppm of the theoretical mass. An MS/MS match required that all major fragments were identified, and that the complete sequence could be inferred from fragment ions containing the 5′ or 3′ ends (not internal fragments). To ensure that the automated software employed stringent MS/MS matching, the software also searched the entire LC-MS/MS dataset against theoretical RNase T_1_ digests of decoy constructs having random arrangements of the same composition of nucleotides as the mRNA molecule. To augment the list of automated software identifications, Excel Visual Basic for Applications (VBA) scripts were employed to examine unidentified LC-UV features and underlying mass spectra one-by-one. Protein Metrics Byos software was employed to characterize the 5′ or 3′ termini, including the 73-mer R1062 and its related poly(A)-tail species. The 5′ or 3′ identifications were made using deconvolved, zero-charge mass spectra, without MS/MS.

The detailed step-by-step method is provided as a Supplementary Data document that describes the enzymatic treatment of the sample, separation, and detection of oligonucleotides by UHPLC-UV, and oligonucleotide identification by high resolution mass spectrometry. It also describes how to use multiple Excel VBA tools, BioPharma Finder software, and Protein Metrics Byos to achieve a heightened characterization of the mRNA digest.

## Results

### Characterization of mRNA primary structure by oligonucleotide mapping

The primary structure of mRNA intended for a vaccine or therapeutic drug is considered a critical quality attribute by regulatory agencies and it must be empirically confirmed for integrity to ensure quality, safety and efficacy. The ideal primary structure characterization technique provides unambiguous elucidation of the full-length mRNA sequence, the 5′ and 3′ termini, and any site-specific modifications by direct measurement of the mRNA molecule.

A single-enzyme oligonucleotide mapping method was developed to directly characterize the Comirnaty BNT162b2 Original mRNA primary structure by combining IP RP-HPLC-UV-MS and MS/MS to separate and identify all oligonucleotides produced via RNase T_1_ digestion. It enabled the detection of 388 oligonucleotides. Seventy-four of these oligonucleotides occur more than once in the construct: they are sequence motif repeats with different starting positions (loci). If such observed oligonucleotides originate from each locus in the construct upon RNase T_1_ digestion, then all 4283 theoretical nucleotides in BNT162b2 have been sampled by the method. Thus, the possible maximum sequence coverage achieved by this method was 100%. The other 314 oligonucleotides each originate from a single locus in the construct. There is no ambiguity in their origin: they account for 2380 nucleotides, giving a unique sequence coverage of 55.6%. With the exception of long poly(A)-tail oligonucleotides, all oligonucleotides were identified by MS/MS fragmentation spectrum matching.

RNase T_1_ digestion of RNA cleaves the phosphodiester backbone on the 3′ side of each guanosine nucleotide and leaves a phosphate on the 3′ carbon of the 3′-end guanosine ribose. Thus, there is no phosphate on the 5′ carbon of the 5′-end nucleotide ribose of a RNase T_1_ digestion product. A missed-cleavage digestion product is an oligonucleotide with one or more internal (non-3′ terminal) guanosine nucleotides. A theoretical RNase T_1_ digestion of BNT162b2 creates 1062 oligonucleotides that group to 302 unique oligonucleotides due to sequence motif repeats in the construct. Of the 388 oligonucleotides identified in the study, 302 are theoretical digestion products of RNase T_1_. The other 86 oligonucleotides include 23 additional poly(A)-tail, 49 missed cleavage, and 14 non-specific cleavage oligonucleotides (Supplementary Data Table 1).

**Figure 1:**
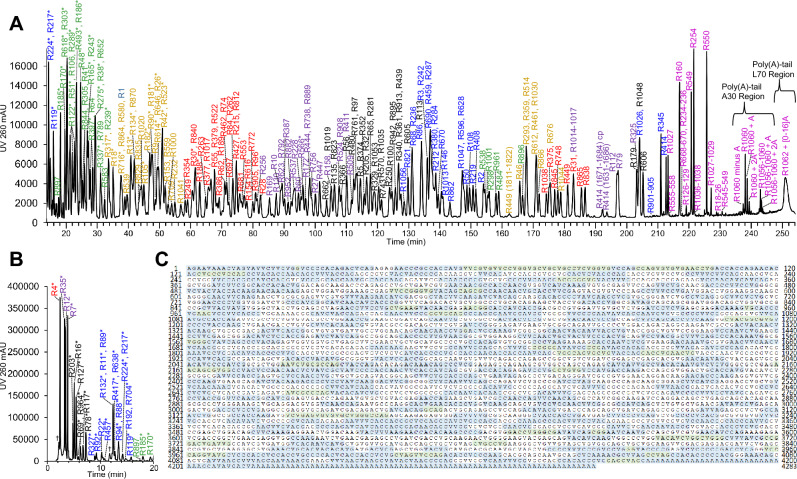
RNase T1 Oligonucleotide Map of BNT162b2 Original DS. (**A**) IP RP-UHPLC-UV-MS/MS RNase T_1_ oligonucleotide map of BNT162b2 Original DS, 14–254 min. “R” represents oligonucleotide RNase T_1_ digestion products indexed from the 5′ to 3′ end. “*” denotes a sequence-repeat oligonucleotide, where the single peak assignment represents all identical oligonucleotides in the sequence. Each color distinguishes the number of nucleotides per digestion product: blue: 4, 10 and 16; green: 5 and 11; gold: 6 and 12; red: 7 and 13, purple: 8 and 14, black: 9 and 15, magenta: > 16. For graphical clarity, not all observed oligonucleotides are annotated on the chromatogram; a complete list is in Supplementary Data Table 1. (**B**) IP RP-UHPLC/UV/MS/MS RNase T_1_ oligonucleotide map of BNT162b2 Original DS, 0–20 min. “R” represents oligonucleotide RNase T_1_ digestion products indexed from the 5′ to 3′ end. “*” denotes a sequence-repeat oligonucleotide, where the single peak assignment represents all identical oligonucleotides in the sequence. Each color distinguishes the number of nucleotides per digestion product: red: 1; purple: 2; black: 3; blue: 4; green: 5. For graphical clarity, not all observed oligonucleotides are annotated on the chromatogram; a complete list is in Supplementary Data Table 1. (**C**) The 55.6% unique sequence coverage is illustrated as shaded by blue and green, based on 314 observed unique-sequence oligonucleotides. Blue nucleotides comprise unique-sequence RNase T_1_ oligonucleotides; green nucleotides comprise unique-sequence missed-cleavage and fragment oligonucleotides. Green and white nucleotides also comprise repeat-sequence RNase T_1_ oligonucleotides, based on 74 observed repeat-sequence oligonucleotides. “V” is N1-methyl pseudouridine.

The first readout of oligonucleotide mapping is a fully annotated UV chromatogram of the RNase T_1_ digestion products (Fig. 1A), which is generated by matching the retention times of each oligonucleotide identified by MS to its corresponding UV peak. In general, the method separates species by the number of nucleotide residues, with shorter oligonucleotides eluting before longer oligonucleotides. The dominant stationary reversed phase-analyte interactions are with triethylammonium-phosphodiester backbone ion pairs. For each subset of oligonucleotide lengths, elution order is influenced by the composition of nucleobases and sequence. In particular, the 5′-end nucleotide influences this order. For oligonucleotides of the same length, the elution order tends to be C first, then V, then A (V represents N1-methyl pseudouridine; Supplementary Data Fig. 1).

Because of sequence motif repeats, some features in the oligo map originate from more than one digestion locus. For example, the “AAAG” digestion product eluting at 15.8 min is a sequence-repeat oligonucleotide which may originate from one or up to all 4 of its loci in the sequence. The first AAAG locus in the BNT162b2 construct is at nucleotide residues 514-517; it follows the 118th G counting from the 5′ end and is thus designated as the “R119” oligonucleotide. This oligonucleotide 4-mer has four UV 260 nm chromophores, and its LC/UV peak area is 2.88 × 10^5^ (Supplementary Data Table 1). No other oligonucleotides co-elute with this species. The 15-mer R606 with sequence ACCCCVCCVAVCAAG elutes at 205 min with no co-eluting species and peak area of 2.55 × 10^5^. By similarity in peak areas, and in stoichiometry of chromophores, it is reasonable to conclude that the 16 min AAAG feature is comprised of all four RNase T1 AAAG oligonucleotide digestion products (R119, R731, R914, and R1046). Observed LC/UV peak areas of all peaks were compared to their predicted (theoretical) peak areas given their oligonucleotide assignment, showing that LC-UV peaks comprised of sequence-repeat oligonucleotides have contributions from all of their loci (Supplementary Data Fig. 2). Thus, oligonucleotide mapping of BNT162b2 achieved a possible maximum sequence coverage of 100%.

To simplify the oligonucleotide map chromatogram in Fig. 1, repeat sequences were annotated with the first locus instance with an asterisk suffix; thus the 16 min feature is “R119*”. Most “*” peaks are small oligonucleotides: 1-mers (G), 2-mers (AG, CG, VG), 3-mers (AAG, etc.); the largest is an 8-mer, VACAVCVG, occurring at two sites (R566 and R947). These repeat sequences make up a significant portion of the BNT162b2 construct. The early UV chromatogram (Fig. 1A and B) shows peaks representing repeat sequences have significant peak areas, commensurate with the number of loci for each species (Supplementary Data Table 1).

While missed-cleavage species are detected at low levels, these abundant repeat sequences indicate the RNase T_1_ digest is largely complete at 90 min. Similar to the conventional treatment of non-unique peptides when performing peptide mapping by LC–MS/MS^24^, each locus of a non-unique oligonucleotide is considered in the determination of maximum sequence coverage. This is also warranted given the correlation between observed and predicted UV peak areas (Supplementary Data Fig. 2).

The second readout of oligonucleotide mapping is the unique sequence coverage map of each nucleotide detected (Fig. 1C). By considering the subset of oligonucleotides that have only 1 locus, the unique sequence coverage was 55.5%. All theoretical RNase T_1_ digest unique-sequence oligonucleotides were observed.

### mRNA construct comparability and identity by oligonucleotide mapping

**Figure 2:**
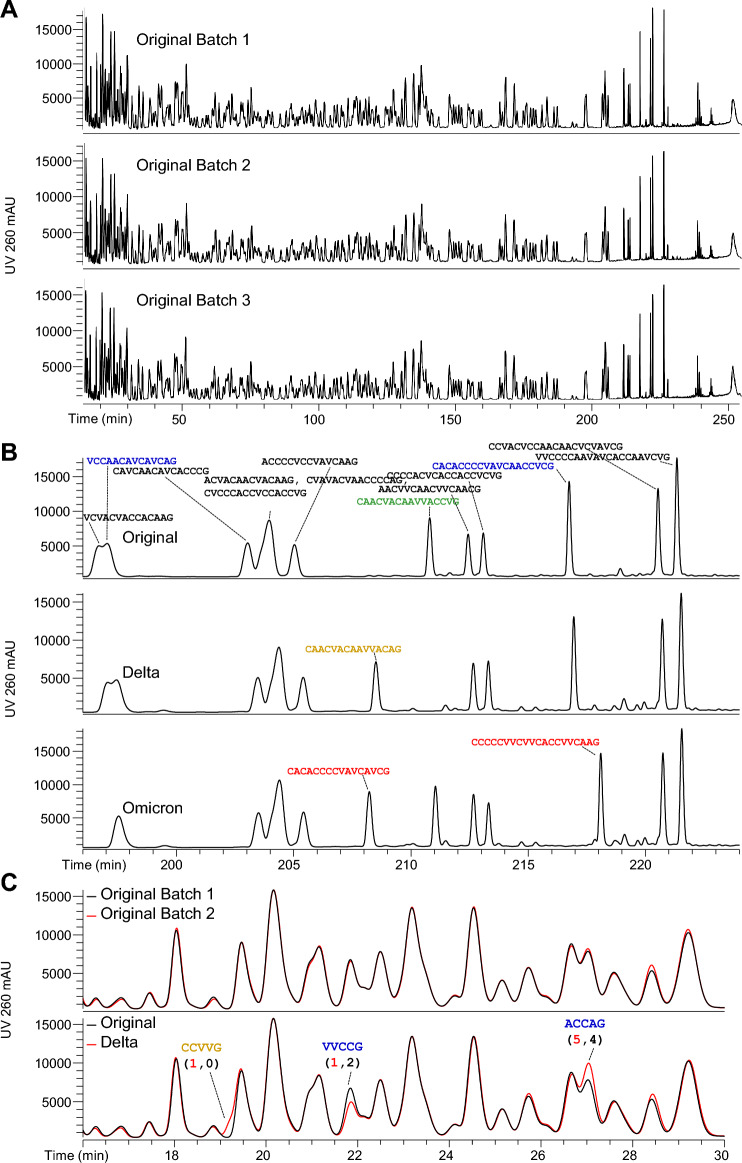
Batch Comparability and Construct Identity Assayed by Oligonucleotide Mapping. (**A**) Three GMP-manufactured BNT162b2 Original DS batches were evaluated by oligonucleotide mapping. The resulting UV chromatograms are visually comparable, demonstrating process consistency. Supplementary Data Fig. 3 provides a six-segment magnification of these data. Batch 3 was made at small scale; Batches 1 and 2 were made by the same GMP process. (**B**) Partial oligonucleotide maps of the BNT162b2 Original, BNT162b2 Delta, and BNT162b2 Omicron mRNA DS. RNase T1-digest oligonucleotide sequences shown in black are shared by all 3 variant constructs; blue sequences are shared by BNT162b2 Original and BNT162b2 Delta constructs; green is shared by BNT162b2 Original and BNT162b2 Omicron constructs; and orange and red oligonucleotides are unique to the BNT162b2 Delta and BNT162b2 Omicron constructs, respectively. (**C**) Partial oligonucleotide maps of two batches of BNT162b2 Original overlaid (top pane), and one BNT162b2 Original batch overlaid with BNT162b2 Delta (bottom pane). Differences between the BNT162b2 Original and BNT162b2 Delta construct chromatograms are annotated with the oligonucleotides accounting for the difference. Blue oligonucleotides are shared by BNT162b2 Original and BNT162b2 Delta constructs; the orange oligonucleotide is unique to the BNT162b2 Delta construct. The numbers in parentheses count the number of sequence repeats in each construct sequence: red signifies the number of occurrences in the BNT162b2 Delta construct and black signifies the number of occurrences in the BNT162b2 Original construct.

Clinical and commercial manufacturing process changes (e.g., site, scale) are common during mRNA vaccine production, and analytical techniques must demonstrate product comparability for pre- and post-change batches^25^. Oligonucleotide mapping provides a direct, detailed assessment of mRNA primary structure comparability across multiple mRNA DS batches. This is analogous to application of peptide mapping by LC-UV-MS/MS for comparability assessment of therapeutic protein batches^26^. The mRNA primary structure of three commercial BNT162b2 batches were deemed comparable (Fig. 2A), as demonstrated by the superimposition of the full-length chromatograms and by the superimposition of zoomed segments of the chromatograms (Supplementary Data Fig. 3).

In a similar comparative analysis, oligonucleotide mapping can identify unique and subtle variance in mRNA primary structure. The SARS-CoV-2 Delta and Omicron variant vaccine construct sequences, BNT162b2 Delta and BNT162b2 Omicron, are 99.6% and 98.6% similar to BNT162b2 Original as shown in Supplementary Data Fig. 4. The mRNA primary structures of all three constructs exhibited distinct peak profile differences by oligonucleotide mapping (Fig. 2B), owing to oligonucleotides that are present in or are absent from at least one of the three. Of the two Original, one Delta, and 15 Omicron oligonucleotides that are unique to their construct, 16 were clearly differentiated by UV and by extracted ion chromatogram analysis in the expected manner (absent in two construct maps, present in one; Supplementary Data Fig. 5). Seventeen of the 18 had diffinitive MS/MS. Only the Omicron-unique 4-mer VCAG co-eluted with sequence isomers that precluded confident MS/MS identification.

Oligonucleotide mapping also reveals subtle differences in primary structure across these variant constructs. Three conspicuous differences are observed between BNT162b2 Original BNT162b2 Delta oligonucleotides in the 16–30 min chromatographic window (Fig. 2C). The first difference is an elevated front shoulder of the 19 min Delta UV peak. This is explained by the oligonucleotide CCVVG, identified in the BNT162b2 Delta map but not the BNT162b2 Original map. It is an expected RNase T_1_ digest product only from the BNT162b2 Delta sequence, and it represents one point of difference between the BNT162b2 Original and BNT162b2 Delta sequences. The UV peak at 21.8 min, identified as VVCCG, is less abundant in the BNT162b2 Delta chromatogram than in the BNT162b2 Original chromatogram. This occurs because it is a sequence-repeat oligonucleotide with two loci in the BNT162b2 Original and one locus in the BNT162b2 Delta sequence. Conversely, the UV peak at 27.0 min, identified as ACCAG, is more abundant in Delta relative to BNT162b2 Original because this oligonucleotide originates from five loci in the former sequence and four loci in the latter.

Importantly, no other differences are apparent in this 16–30 min chromatographic window (Fig. 2C), consistent with the theoretical tabulation of expected RNase T1 digest oligonucleotides. Exact overlap between the chromatograms of these variants shows they have the same stoichiometric number of a single oligonucleotide or set of oligonucleotides. Moreover, comparative analysis of BNT162b2 Original vs Delta by oligonucleotide mapping provides an important visual counterpoint of batch-to-batch comparison, in which the claim of visual comparability by superimposition is appropriate.

### Oligonucleotide mapping of mRNA enables simultaneous characterization of the 5′ and 3′ Termini without affinity purification

**Figure 3:**
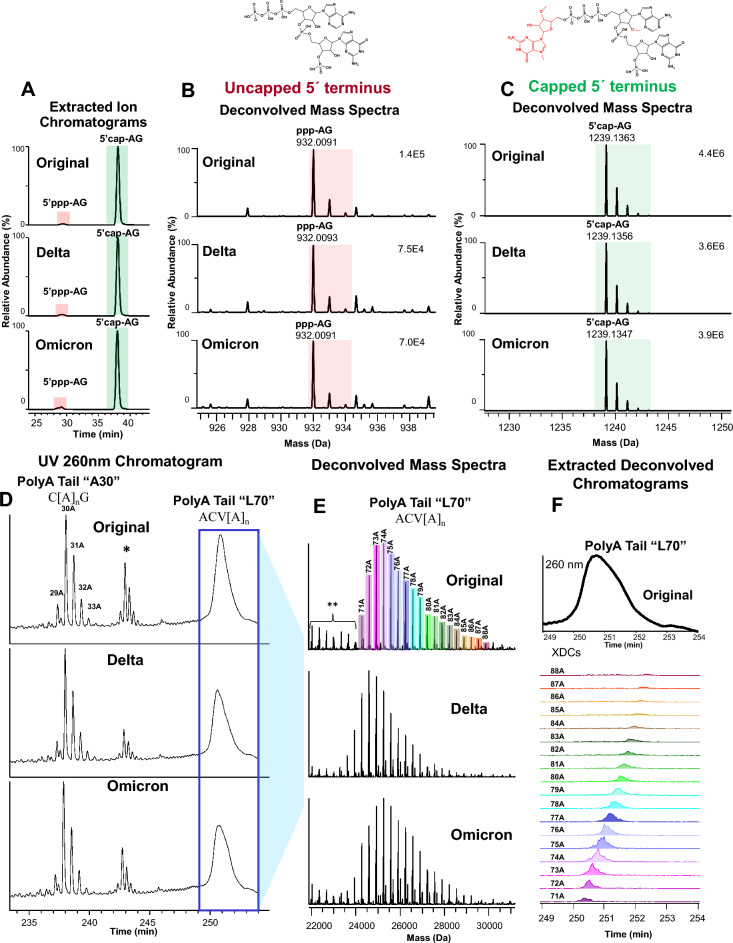
Oligonucleotide Mapping of mRNA Enables Simultaneous Characterization of the 5′ and 3′ Termini Without Affinity Purification. (**A**) Extracted ion chromatograms ([M-1H]^1–^, [M-2H]^2–^) of uncapped (5′ppp-AG) and capped (5′ cap-AG) versions of the 5′ terminal oligonucleotide for BNT162b2 variant constructs Original, Delta, and Omicron. (**B**, **C**) Deconvoluted, zero-charge mass spectra of uncapped (5′ppp-AG) and capped (5′ cap-AG) versions, respectively, of the 5′ terminal oligonucleotide for BNT162b2 variant constructs Original, Delta, and Omicron. The spectra are deconvoluted from the summation of scans within the regions highlighted in panel A, using Byos v4.4 (Protein Metrics). Observed masses (monoisotopic) agree with theoretical masses to within 3 ppm, which is consistent with the accuracy and precision of Orbitrap mass spectrometers. (**D**) UV chromatograms (260 nm) of the poly(A)tail region for BNT162b2 variant constructs Original, Delta, and Omicron: the A30 and L70 poly(A) oligonucleotide regions consisting of the generic formulas C[A]_n_G, and ACV[A]_n_, are labeled accordingly. Chromatographically separated A30 poly(A) peaks are labeled according to the number of adenosines detected in each oligonucleotide. The asterisk (*) denotes a separate A30 poly(A) oligonucleotide distribution resulting from missed cleavage of the A30 poly(A) oligonucleotide, consisting of the generic formula CCACACCCVGGAGCVAGC[A]_n_G. The blue box highlights the chromatographic distribution of L70 poly(A) oligonucleotides (unseparated) which are further described in panels (**E** and **F**). (**E**) Deconvoluted, zero-charge mass spectra of the L70 poly(A) oligonucleotide distribution from the summation of scans within the blue boxed region highlighted in panel (**D**). Mass spectral peaks are labeled according to the number of adenosines detected in each L70 poly(A) oligonucleotide. The double asterisk (**) denotes separate L70 poly(A) oligonucleotide distributions resulting from artefactual degradation of the L70 poly(A) oligonucleotides during sample preparation. Observed masses (monoisotopic) agree with theoretical masses to within 5 ppm, which is consistent with the accuracy and precision of Orbitrap mass spectrometers. ~ 1 and 2 Da mass errors from de-isotoping occurred on certain species in the L70 poly(A) distributions because of the trace-level relative abundance of these species or signal interference from other species; more specifically, insufficient signal-to-noise or interference results in non-statistical isotope distributions leading to errors in the monoisotopic mass determination. (**F**) Extracted deconvolved chromatograms (XDCs) of BNT162b2 Original L70 poly(A) oligonucleotide distribution highlighted in panel (**E**). Colored shading on each oligonucleotide in panel (**E**) corresponds to its XDC.

Proper capping of the 5′ terminus and appropriate length of the poly(A) 3′ end are critical quality attributes for an mRNA vaccine or therapeutic. The oligonucleotide map developed here enables direct characterization of the 5′ cap and 3′ poly(A)tail in a single technique, without the need for isolation and purification of either terminus (Fig. 3). Extracted ion chromatograms of the 5′ terminus (Fig. 3A) and the accompanying deconvolved mass spectra demonstrate unambiguous detection of trace-level uncapped species (5′ppp-AG as denoted in Fig. 3A and B) relative to the properly capped form (5′ cap-AG as denoted in Fig. 3A and C) in BNT162b2 Original, Delta, and Omicron, using high resolution accurate mass. The majority of the 5′ end is properly capped in each construct (this was confirmed by an orthogonal LC-UV-based analysis—data not shown).

The DNA plasmid template-encoded poly(A)tail of BNT162b2 Original, A30L70, consists of a stretch of 30 adenosine residues (A30 segment), followed by a 10-nucleotide linker sequence and 70 additional contiguous adenosine residues (L70 segment). Due to transcriptional slippage of the IVT T7 polymerase^27^, more than one poly(A)-tail species is observed. The A30 poly(A) distribution is chromatographically resolved at single nucleotide resolution by the oligonucleotide map (Fig. 3D) and confirmed by mass spectrometric profiling (data not shown). This confirms the majority of the A30 poly(A) segment lengths in BNT162b2 Original, Delta, and Omicron fall within a range of 29-33 adenosine residues.

The distribution of L70 poly(A) segment elutes as a single broad chromatographic peak. The oligonucleotide map is specifically tuned for proper MS detection of larger RNase T_1_ digestion products in this segment of the chromatogram to characterize the distribution of the L70 poly(A) species (Fig. 3E). The observed monoisotopic masses of the L70 poly(A) species were assigned based on accurate mass agreement with expected theoretical masses. Oligonucleotide mapping confirmed that the majority of L70 poly(A) segment lengths ranged from 71 to 88 in BNT162b2 Original. Extracted zero-charge chromatograms of the BNT162b2 Original L70 poly(A) distribution demonstrates that increasing elution time correlates with increasing poly(A) length (Fig. 3F). Thus, the L70 poly(A) length is a true reflection of the mRNA construct and not artifactual fragmentation induced by electrospray ionization in the mass spectrometer. Furthermore, the oligonucleotide map has the sensitivity and specificity to detect subtle shifts in the L70 poly(A) distribution owing to transcriptional slippage^28^: the L70 poly(A) distributions of Delta and Omicron are slightly shorter than BNT162b2 Original (Fig. 3E).

### MS/MS fragmentation is a critical component of oligonucleotide mapping of mRNA

Oligonucleotide mapping typically requires complete MS/MS fragment ion ladders for proper identification across a diverse array of sequence lengths (2-mers to > 20-mers). Of the 302 unique oligonucleotide sequences generated by an RNase T_1_ in silico digestion of BNT162b2 Original, 220 are sequence isomers. These share the same composition, and therefore mass, with at least one other oligonucleotide, and many differ only by a single nucleotide exchange between two positions. Sequence isomers require high quality MS/MS spectra for identification.

**Figure 4:**
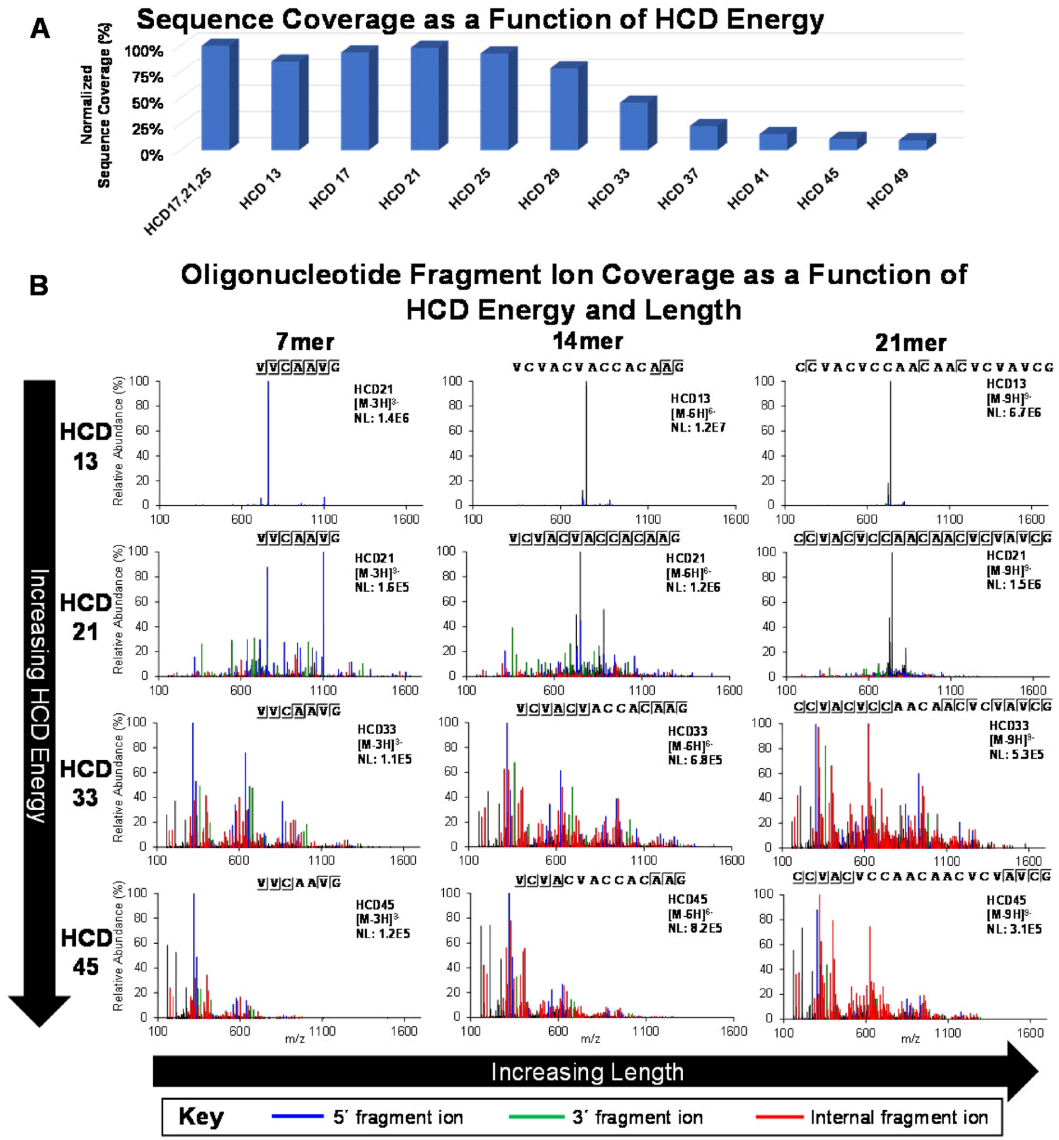

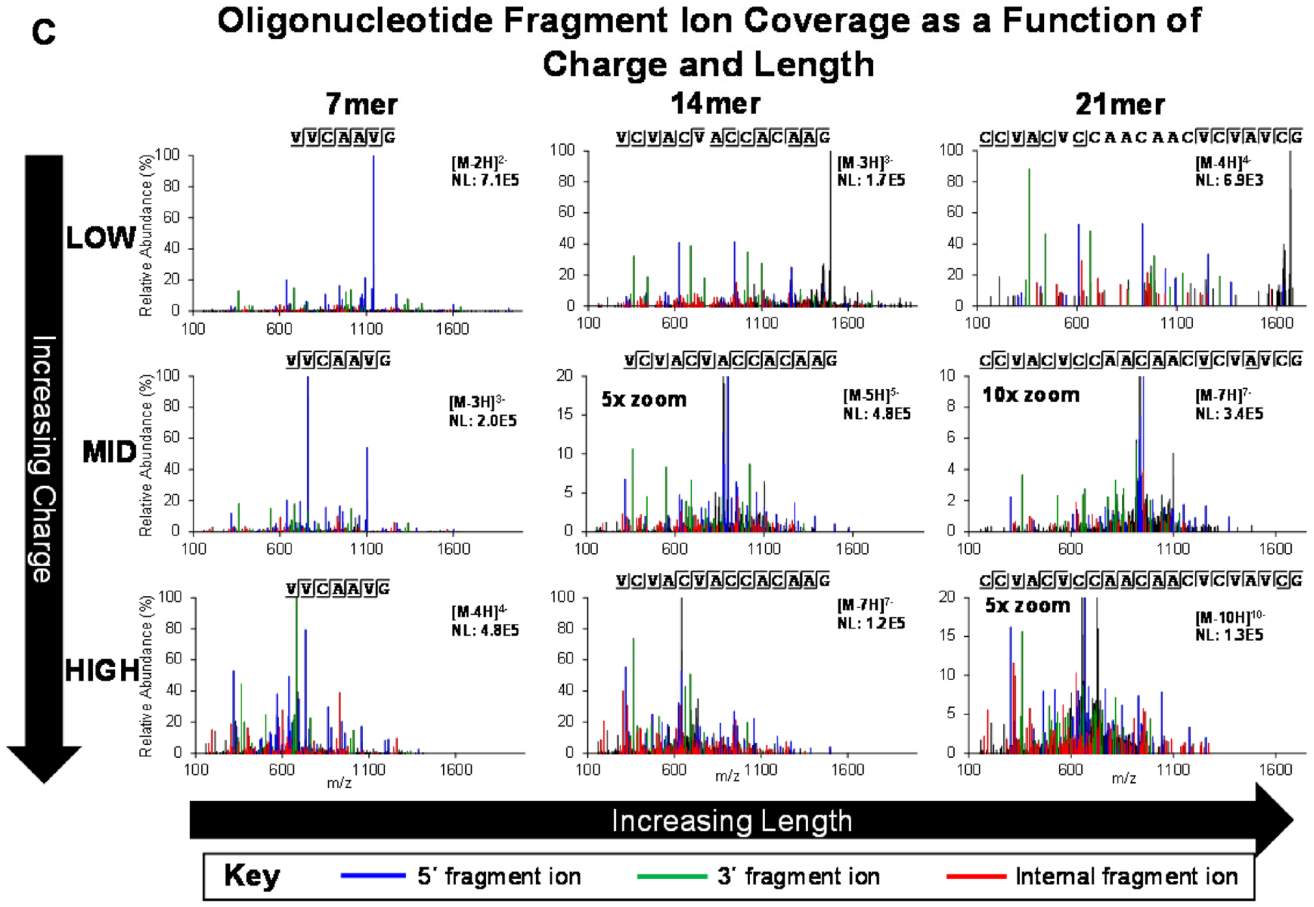
Optimal HCD energy and charge density drive proper fragmentation. (**A**) Relative BNT162b2 Original maximum sequence coverage determined by oligonucleotide mapping as a function of HCD energy. The maximum sequence coverage resulting from each condition is normalized to the final recommended condition (stepped HCD 17, 21, 25). Maximum sequence coverage was restricted only to oligonucleotides identified by BioPharma Finder using a confidence score parameter restriction of 100%. (**B**) MS/MS spectra and fragment ion coverage of 3 oligonucleotides, presented as a function of HCD energy and oligonucleotide length. The MS/MS spectra are generated using HCD energies: 13, 21, 33, and 45, as applied to 7mer, 14mer, and 21mer oligonucleotides. Fragment ions identified as 5′ (a,b,c,d), 3′ (w,x,y,z), and internal fragments are annotated by color-coding as defined in the key. Identification and annotation of fragment ions was performed using a Visual Basic Excel tool developed in-house. (**C**) MS/MS spectra and fragment ion coverage of 3 oligonucleotides, presented as a function of oligonucleotide charge and length. MS/MS spectra are generated using a single stepped energy HCD method (17, 21, 25) on low, middle, and high charge states of the same 7mer, 14mer, and 21mer oligonucleotides presented in panel B. Fragment ions identified as 5′ (a,b,c,d), 3′ (w,x,y,z), and internal fragments are annotated by color-coding as defined in the key. Identification and annotation of fragment ions was performed using a Visual Basic Excel tool developed in-house.

Historically, oligoribonucleotide MS/MS fragmentation has been performed using collision induced dissociation (CID)^29,30^. In this work, we used an updated version of the technique, higher energy collisional dissociation (HCD), for oligonucleotide mapping. Experimental studies were performed to examine the effects of HCD energy, oligonucleotide length, and charge state on oligonucleotide fragment ion types and the extent of contiguous fragmentation along the RNA backbone for optimal sequence coverage. In general, fragmentation patterns were complex, often including all four main types of 5′ (a,b,c,d) and 3′ (w,x,y,z) terminal fragment ions^31^. Some HCD spectra contained fragment ions missing a unique identifying base (-B) and internal fragments born from more than one phosphodiester bond breakage. We observe that internal fragments are of limited use for inferring the sequence of the putative oligonucleotide, and they decrease the quality of the spectra by degrading informative terminal fragments and adding interferant masses. To assess the effect of HCD energy on spectra across the entirety of the oligonucleotide map, mRNA construct sequence coverage was monitored. Fixed HCD energies 17, 21, and 25 were optimal (Fig. 4A) amongst a series of single-energy fixed HCD MS/MS acquisitions. The highest mRNA construct sequence coverage was obtained by combining these into a stepped HCD 17, 21, 25 method.

These results are specifically understood by examining fragment ion coverage produced in MS/MS spectra as a function of HCD energy and oligonucleotide length (Fig. 4B). The oligonucleotide fragment ion charge densities were held constant for all spectra in this example: 2.3 nucleotides per charge. Mass Spectra of shorter oligonucleotides typically contained a full range of discernable fragment ions regardless of HCD energy. HCD energy had a greater effect on longer oligonucleotides; lower energies did not produce adequate levels of productive fragment ions for sequencing. As HCD energy is increased, 5′ (a,b,c,d) and 3′ (w,x,y,z) terminal fragment ions amenable for sequencing increase in abundance. HCD energy is positively correlated with the abundance of internal fragment ions, such that HCD energy over 25 is not recommended. Sequence discernment is lost from the middle of the oligonucleotides when longer terminal fragments are further fragmented to internal fragments. This trend continues as HCD energy increases and only the shortest oligonucleotides remain, some of which are short terminal fragments. An HCD energy of 21 produced an ideal balance of relatively abundant terminal fragment ions for sequencing and mitigation of internal fragmentation.

MS/MS fragment ion coverage was also studied as a function of charge state and oligonucleotide length (Fig. 4C). The HCD energy is held constant at the recommended condition of stepped HCD collision energies: 17, 21, 25. For all oligonucleotide lengths, the lowest charge states generally produced the lowest fragment ion coverage, and the highest charge states generally produced the highest fragment ion coverage. Fragment ions from a lower charge state precursor may enable additional sequence coverage at a specific position in some cases for two main reasons: (1) the higher charge state has a significantly lower abundance than a lower charge state and/or (2) higher-charged fragment ions overlap with lower-charged fragment ions, confounding the identity of both.

We observed that increasing oligonucleotide charge density had a positive effect on sequencing-enabling fragmentation. For example, the [M-4H]^-4^ charge state of the 7-mer (1.75 nucleotides/charge) produces abundant fragment ions and full fragment ion coverage (Fig. 4C). However, the [M-4H]^-4^ charge state of the 21-mer (5.25 nucleotides/charge) produces relatively weak fragment ions, only allowing sequencing of the first few nucleotides on each terminus. This phenomenon is most pronounced as oligonucleotide length increases. MS/MS sequencing of oligonucleotides with charge densities <  ~ 2.5 combined with stepped HCD 17, 21, 25 provided suitable fragmentation across the oligonucleotide map. Fortunately, IP RP-UHPLC-ESI MS conditions generate progressively higher charge states with later eluting larger oligonucleotides; this maintains ideal charge density for MS/MS sequencing. Typically, little value was added by MS/MS sequencing two different charge states of the same oligonucleotide.

### Resolving sequence isomers by MS/MS

**Figure 5:**
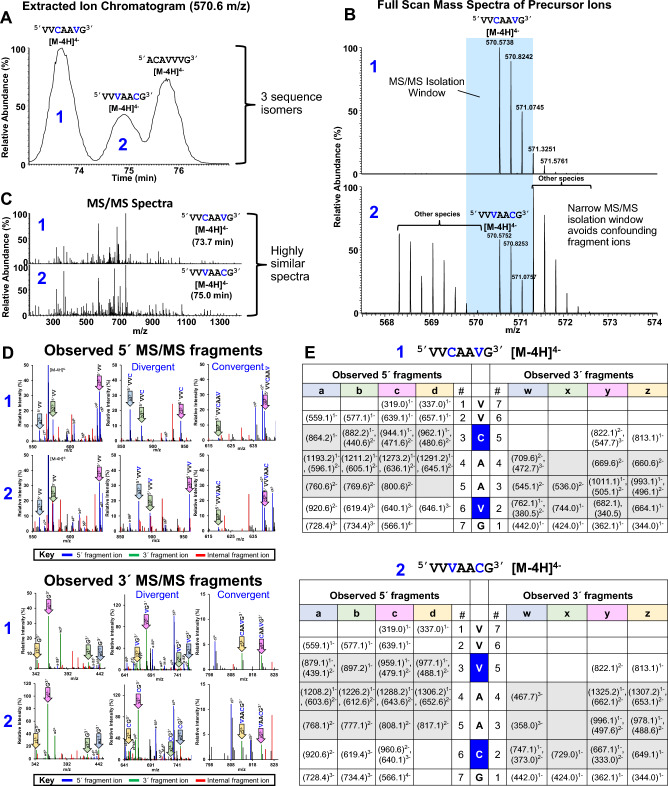
Proper MS/MS fragmentation & interpretation is critical for oligonucleotide mapping. (**A**) Extracted ion chromatogram of 3 oligonucleotide sequence isomers ([M-4H]^4−^). (**B**) Full scan mass spectra of sequence isomers “1” and “2” ([M-4H]^4−^) as denoted in panel (**A**). The blue shading defines the precursor isolation window used for subsequent MS/MS fragmentation. (**C**) MS/MS fragmentation spectra derived from sequence isomers “1” and “2” ([M-4H]^4−^). (**D**) Zoomed sections of sequence isomers “1” and “2” ([M-4H]^4−^) in MS/MS spectra, with annotation of select 5′ and 3′ fragment ions. The 1st, 2nd, and 3rd columns in the observed 5′ MS/MS fragments pane (top) highlight 5′ fragments identified for positions 2, 3, and 6, respectively. The 1st, 2nd, and 3rd columns in the observed 3′ MS/MS fragments pane (bottom) highlight 3′ fragments identified for positions 1, 2, and 5, respectively. For each panel, the “divergent” label denotes that the masses of the same fragment ions between sequences isomers “1” and “2” diverge at that position, indicating they contain different nucleotides at that position. The “convergent” label denotes that the masses of the same fragment ions between sequences isomers “1” and “2” converge at that position, again indicating they contain different nucleotides at that position. Color-coding of the spectral peaks is defined in the key. Unique colored shading of each arrow highlighting fragment ions corresponds to the colors as defined in panel (**E**). (**E**) Observed fragment ion mass tables for sequence isomers “1” and “2” ([M-4H]^4−^). Unique colored shading defines each type of 5′ fragment ion and its 3′ pair and matches the shaded arrows in panel (**D**). The dark blue shading highlights the two bases which change position between the two sequence isomers. The gray shading highlights the fragment ion masses which differentiate the two sequence isomers from each other.

Optimal HCD fragmentation enabled successful differentiation of nearly all sequence isomers in the oligonucleotide map. Figure 5 demonstrates this with a challenging but common scenario. Three sequence isomers are pictured in an extracted ion chromatogram (Fig. 5A). Isomer “2” eluting at 75 min coelutes with other oligonucleotides of highly similar mass (Fig. 4C), which increases the risk of isolating two unrelated oligonucleotides to produce a mixed MS/MS spectrum. The oligonucleotide map employs an MS/MS isolation window (1.5 m/z) which balances the need to minimize incidences of mixed spectra (Fig. 5B and C) and sample the isotopic distribution to enable fragment ion charge state determination. Sequence isomers “1” and “2” are highly similar, differing only by a single exchange of the 3rd and 6th nucleotides (Fig. 5C). Therefore, their MS/MS spectra are highly similar, but a few key fragment ions distinguish each sequence isomer.

Reading from the 5′ end (Fig. 5D and E), terminal fragment ions ending at positions 1 and 2 have identical masses for each sequence isomer. Terminal fragment ions ending at positions 3–5 have unique, divergent masses in each sequence isomer, indicating a difference in sequence at position 3. Terminal fragment ions ending at position 6 converge in mass, indicating another difference in sequence between sequence isomers at position 6.

Reading from the 3′ end (Fig. 5D and E), terminal fragment ions at position 1 have identical masses for each sequence isomer. Terminal fragment ions ending at positions 2–4 have unique masses for each sequence isomer, indicating a difference in sequence at position 2. Terminal fragment ions ending at position 5 converge in mass, indicating another difference between sequence isomers in sequence at position 5.

In both cases, detecting sequence differences are predicated on having a complete fragment ion ladder. MS/MS fragmentation by oligonucleotide mapping produced complete complementary fragment ion ladders for most sequence isomers, enabling their unambiguous identification and testifying to the suitability of the parameters for oligonucleotide mapping.

### Comprehensive, automated, high-fidelity data analysis

Comprehensive, high-fidelity analysis of oligonucleotide mapping data requires automation for practical ease-of-use and efficiency. In-house Excel Visual Basic for Applications (VBA) scripts combined with in-development beta and commercial vendor software, were used to automate data analysis of oligonucleotide mapping. Together, these tools facilitated comprehensive primary structure characterization of BNT162b2 Original via oligonucleotide mapping with complete UV peak annotation and 100% maximum sequence coverage.

A custom Excel VBA script automatically correlated oligonucleotides identified by LC-MS/MS to their corresponding LC-UV feature and automatically annotated the entire UV chromatogram (Supplementary Data Fig. 6 illustrates the entire workflow). Identifications were provided by one of two methods: (1) 280 of 388 (72%) oligonucleotides were identified by BioPharma Finder (Thermo Fisher Scientific), (2) 108 of 388 (28%) were identified using custom Excel VBA scripts. The scripts facilitated semi-automatic oligonucleotide identification by the following procedure: (1) observed precursor masses associated with unidentified LC-UV features were matched to possible theoretical digest oligonucleotides, (2) for each unknown, empirical MS/MS m/z-peak intensity coordinates, observed mass, charge state, and a hypothesized oligonucleotide from the candidate list were input to an MS/MS spectrum analyzer, (3) an annotated MS/MS spectrum and corresponding sequencing table were automatically generated, (4) the MS/MS sequencing match for the hypothesized oligonucleotide was reviewed by an analyst, confirming or rejecting the identification.

**Figure 6:**
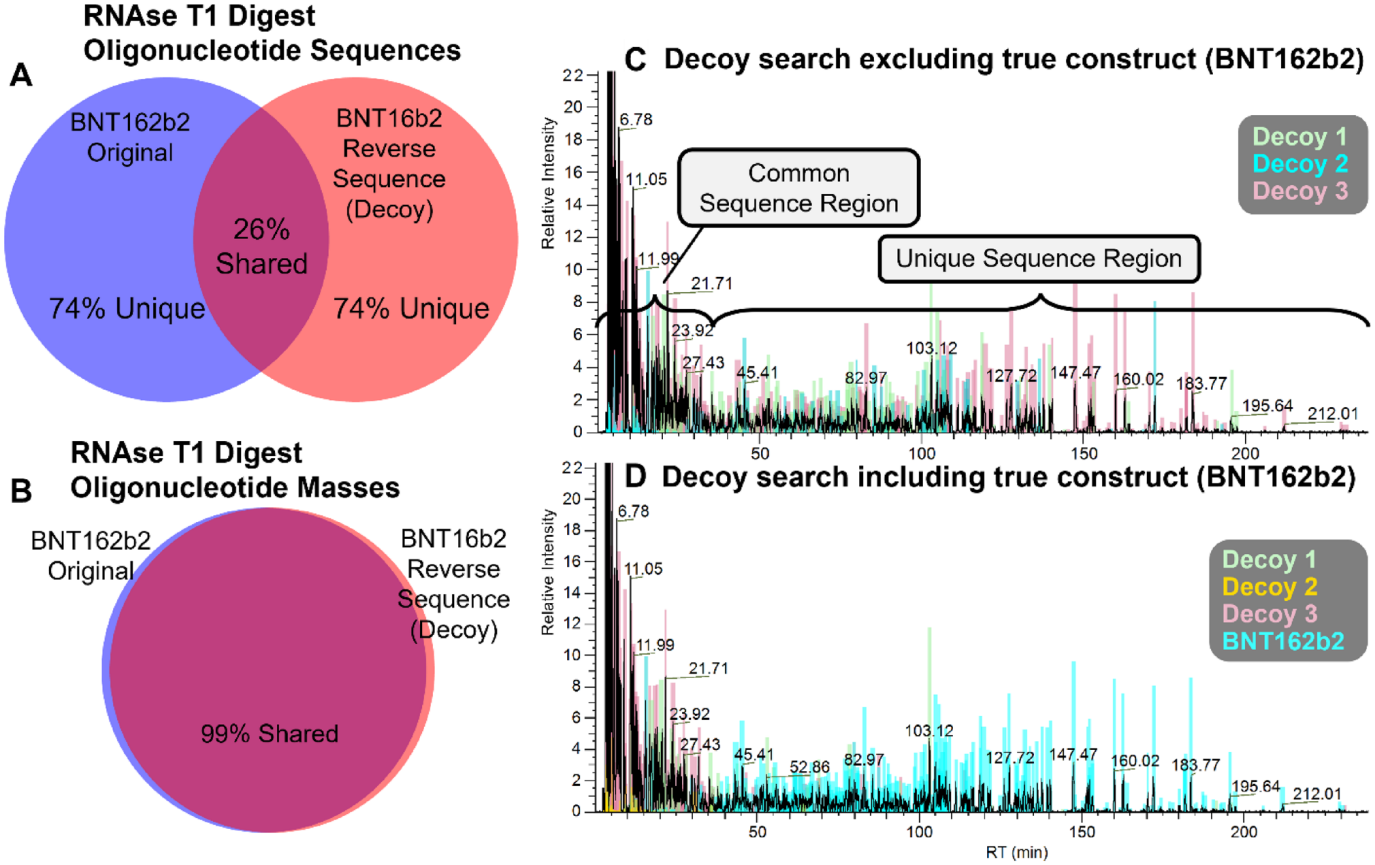
Decoy sequence search technique developed as a suitability assessment for oligonucleotide mapping workflow. (**A**) Venn diagram of the shared and unique oligonucleotide fragments of a theoretical RNase T_1_ digestion of BNT162b2 Original construct and its reverse-sequence construct. This is one example of a decoy construct. The theoretical digestion predicts 302 unique-sequence oligonucleotides (74%) for each and 78 shared oligonucleotides (26%). (**B**) Venn diagram of the shared-mass and unique-mass oligonucleotide fragments of a theoretical RNase T_1_ digestion of BNT162b2 Original construct and of its reserve-sequence construct. The theoretical digestion predicts 147 unique-mass oligonucleotides for each. Of these, 145 oligonucleotides (99%) are shared. (**C**) Decoy construct identification overlay on the base peak ion chromatogram of the BNT162b2 Original RNase T_1_ digest acquisition. Each colored bar marks an oligonucleotide feature identified by the BioPharma Finder automated software as originating from the RNase T_1_ digest of a decoy construct. The decoy constructs are random sequences containing the nucleotide composition of BNT162b2 Original. The common sequence elution region contains shorter oligonucleotides, most of which are common to any decoy constructs as well as the true construct when subjected to RNaseT_1_ digestion. The unique sequence elution region contains longer oligonucleotides, most of which are unique to the true construct. The true construct oligonucleotides are similar enough to decoy sequence oligonucleotides for the automated software to provide decoy oligonucleotide assignments. The identifications in the unique sequence elution region are not preferentially assigned to a single decoy construct, demonstrating that none of the decoy constructs are the true construct. (**D**) Target and decoy construct identification overlay on the base peak ion chromatogram of the BNT162b2 Original RNase T_1_ digest acquisition. Each colored bar marks an oligonucleotide feature identified by the BioPharma Finder automated software as originating from the RNase T_1_ digest of a decoy or target construct. Oligonucleotides in the unique sequence elution region are mostly identified (> 95%) as belonging to the BNT162b2 Original construct. This confirms the identity of the true construct and verifies the oligonucleotide mapping workflow’s ability to correctly identify oligonucleotides via LC-MS/MS.

Oligonucleotide mapping data analysis is challenged by the abundance of sequence isomers that have highly similar MS/MS spectra. There is also a high likelihood that many of the RNase T_1_ digestion products for a large (e.g. ~ 1 + MDa) target construct will have identical masses and high sequence similarity to the digestion products of other constructs with similar size and composition. For example, the reverse sequence of BNT162b2 has the same number of in silico RNase T_1_ digestion products as the forward sequence; but only 26% of them have the same sequence (Fig. 6A). However, 99% have the same mass (Fig. 6B), making high quality MS/MS and high-fidelity interpretation of those spectra critical for their identification, and the identification of the correct construct.

Due to this challenge, a suitability assessment performed on a comprehensive scale is necessary for the automated oligonucleotide data analysis. The first step of the strategy also illustrates the inherent problem. Automated identification of BNT162b2 oligonucleotides is performed against decoy constructs generated by randomly scrambling the sequence (Fig. 6C). Most oligonucleotides eluting in the “unique sequence region” can only be generated through RNase T_1_ digestion of the true construct, as opposed to the “common sequence region” oligonucleotides that are common to in silico digests of the true construct and one or more decoy constructs. Many unique oligonucleotides are similar to in silico RNase T_1_ digestion oligonucleotides of one or more decoy constructs, such that the software dutifully assigns an identification (albeit incorrectly). This occurs because the MS precursor ion match criterion is met and there is sufficient MS/MS evidence to support a reasonable sequence identification. Importantly, omitting the BNT162b2 construct from the decoy search (Fig. 6C) results in oligonucleotides being assigned to a comparable mix of all three decoy constructs. In contrast, most oligonucleotides in the “unique sequence region” are assigned to the BNT162b2 construct when searched in tandem with the decoy constructs, revealing it to be the true construct (Fig. 6D). This readout is a validation that the combined workflow of (1) one-pot one-enzyme enzymatic digestion, (2) chromatographic separation, (3) MS/MS HCD fragmentation, and (4) semi-automated data analysis is suitable for mRNA primary structure characterization.

While the automated software used in this study, BioPharma Finder, enables overall fidelity checking by decoy searching, for individual MS/MS matches it does not provide a ranking of best and next-best matches, nor a matched probability derived from its confidence scoring. To cross-check individual MS/MS assignments we have also used the publically available Pytheas software package^32^. In this comparison it was not possible to match single fragment spectrum matches between software; instead, the retention times of same-oligonucleotide identifications were compared. The retention times do not perfectly match because Pytheas only interprets MS/MS, such that the retention time of an identification is based on its MS/MS scan event time, whereas BioPharma Finder extracts precursor-ion chromatograms and associates an MS/MS identification with the precursor extracted ion apex retention time. Nevertheless, the 0.9997 correlation coefficient and 0.9993 slope of the linear fit to the plot of retention times (Pytheas, BioPharma Finder) of the 280 BioPharma Finder-identified oligonucleotides proves both software agree with nearly all oligonucleotide identifications (Supplementary Data Fig. 7). This analysis raises an important observation: when LC features are not comprised of sequence isomers, BioPharma Finder and Pytheas automated software identify oligonucleotides equally well. When LC features are mixture peaks of sequence isomers, neither software identify oligonucleotides well (the feature is either unidentified (BioPharma Finder) or improperly identified (Pytheas)), and the analyst must rescue the identification by careful spectrum interpretation using individual spectrum matching software such as the Excel macro-enabled spreadsheets provided in this study.

## Discussion

LC-MS/MS-oligonucleotide mapping was developed to provide direct, comprehensive characterization of mRNA primary structure for the Comirnaty BNT162b2 vaccine against SARS-CoV-2. Using one enzyme, RNase T_1_, the method achieved 100% maximum sequence coverage and sensitive detection of the 5′ and 3′ terminal forms, thereby confirming structural integrity of the intended full-length molecule in a single method. The number of oligonucleotide sequence repeats in mRNA is prevalent after RNase T_1_ digestion, but the method stoichiometrically digested and detected these species, augmenting the 56% unique sequence coverage. Systematic evaluation of the MS/MS parameters led to reliable differentiation of sequence isomers, which are also prevalent in digested mRNA, for a further increase in maximum sequence coverage. Lastly, a decoy sequence data analysis search technique was developed to ensure confidence in automated oligonucleotide assignment. Taken together, the LC-MS/MS-oligonucleotide mapping method described here improves upon existing methods by (1) providing a robust, one-pot single commercially-available nuclease digestion method; (2) ensuring that the most sequencing-informative MS/MS are acquired using optimized HCD fragmentation; (3) providing a simple decoy search suitability assessment to ensure automated software properly interprets MS/MS; (4) providing a tool to enable the proper annotation of the “fingerprint” complex LC-UV chromatogram and (5) providing MS and MS/MS spectrum interpretation tools to check automated software identifications and to identify unknown and sequence isomer mixture peaks.

We recommend a practical suitability assessment to evaluate the entire LC-MS/MS workflow, including automated data analysis (Fig. 6C and D). This decoy search strategy is analogous to what has long been performed for protein inference in proteomic analyses^33^. This oligonucleotide mapping method is not an “-omics” method; irrespective of the 3′ and 5′ end heterogeneity, DS is a single construct, not a complex mixture of thousands of RNA molecules. Nevertheless, decoy searching that enables assessment of MS/MS match quality through relative spectral comparison is appropriate, because sequence isomers from the same construct can have very similar fragmentation patterns—and there are many sequence isomers: 220 of the 302 BNT162b2 mRNA theoretical RNase T_1_ digest oligonucleotides.

Another promising aspect of this MS approach is that by directly characterizing RNA, phosphodiester hydrolysis degradation sites and incomplete transcription sites may also be cataloged and subsequently monitored to understand DS degradation pathways. In addition, it is possible to detect oligonucleotides arising from the transcription of the non-target region of the DNA plasmid (though this was not observed in this study). Lastly, this oligonucleotide mapping method could be further optimized and applied to characterize site-specific modifications including mRNA-lipid adducts^34^ and other possible effect.

There are other good strategies to increase the number of unique oligonocleotides in the RNA map, by either promoting RNase T_1_ missed cleavages in a limited digestion or using endonucleases with low-frequency substrate sites, such as MazF and RNase 4^18,19,35^. The data analysis methodolgy detailed here should work equally well, and moreover may be necessary as the identification of mixture peak components is a problem independent from oligonucleotide uniqueness (though there should be fewer mixture peaks).

Oligonucleotide mapping and Next-Generation Sequencing (NGS) are powerful, orthogonal characterization methods for determination of mRNA primary structure. Both techniques have distinct advantages. NGS can effectively determine the contiguous nucleotide sequence with multiple reads (Supplementary Data Fig. 8), as well as detect and identify any contaminating DNA/RNA. Oligonucleotide mapping is used to confirm structural integrity of the entire mRNA molecule including the nucleotide sequence, degree of capped and uncapped 5′ terminus, and the microheterogeneity of the poly(A)tail region. It also adds a vital capability to assess the comparability of batches after manufacturing process and site changes. It may be use to distinguish different mRNA constructs as an identity assay. No method-specific controls need to be synthesized and maintained (such as heavy isotope-labeled controls).

The oligonucleotide mapping method described here involving 1.5 h RNase T_1_ digestion and IP-RP-UHPLC-UV-MS/MS was used in the development and commercialization of the Comirnaty BNT162b2 vaccine against SARS-CoV-2. The process and product understanding gleaned from oligonucleotide mapping supported both the emergency use authorization (EUA) and biologics license application (BLA) regulatory submissions and contributed to the overall assessment of product quality, safety, and efficacy. Likewise, oligonucleotide mapping has been part of many comparability exercises helping to demonstrate that the highest product quality was maintained as production scales were increased and new manufacturing sites were brought online to meet the critical supply challenge of the COVID-19 pandemic. It is our intention for this method to accelerate the development and regulatory submissions of well-characterized mRNA vaccines and genetic therapies and to advance the science of RNA structural understanding more broadly.

## Supplementary Information

Supplementary Table 1.

Supplementary Information 2.

Supplementary Figures.

## Data Availability

Raw data and a detailed method document are provided in the Dryad Data Platform: https://datadryad.org/stash/share/38WoZ944MVcISX-VQpGNoaXn_4Pwe5nv_ipD707TZF8.

## Code availability

Four Excel VBA software mapping and identification tools used to check and augment Thermo BioPharma Finder identifications and provide an annotated chromatogram are provided in the Dryad Data Platform: https://datadryad.org/stash/share/38WoZ944MVcISX-VQpGNoaXn_4Pwe5nv_ipD707TZF8. The method document provided also describes the click-by-click instructions for each.xlsm files’ use.

## Material availability

The mRNA constructs analyzed were manufactured at Pfizer. Though the sequences are provided in this manuscript, it is not possible to make available any of the mRNA material presented here.
