# Supplementary material for: Oligonucleotide mapping via mass spectrometry to enable comprehensive primary structure characterization of an mRNA vaccine against SARS-CoV-2
Source: Sci Rep. 2023 Jun 3;13:9038. doi: 10.1038/s41598-023-36193-2 (PMC10239040; doi:10.1038/s41598-023-36193-2)
Supplement: Supplementary file 2 — Supplementary Information 2. [file 41598_2023_36193_MOESM2_ESM.docx]

# Purpose

## The purpose of this method is to describe a procedure for the heightened characterization of macromolecular mRNA (e.g. 1000 – 15000 nucleotides) primary structure, including the 5′- and terminus and 3′ Poly(A)-tail, by liquid chromatography tandem mass spectrometry (LC/MS/MS) mapping. This procedure describes the enzymatic treatment of the sample, separation, detection of oligonucleotides by ultra-high-performance liquid chromatography-ultraviolet absorbance (UHPLC/UV), and oligonucleotide identification by high resolution mass spectrometry (HRMS). It also describes in detail how to use multiple software to achieve a heightened characterization of the mRNA digest.

# principle

Heightened characterization by UHPLC/UV/HRMS mRNA mapping is used to confirm the identity of an mRNA structure and assess its comparability across multiple batches. The mRNA drug substance for mRNA vaccines is produced by in vitro transcription using a linear DNA template that codes for the intended RNA primary structure (construct). The intended species is capped at its 5′ end, terminates at the 3′ end with a poly-A tail region of varying length depending on the construct, and typically totals over 1000 nucleotides in length.

Enzymatic digestion of mRNA using RNase T1 occurs at the 3′ end of guanosine nucleotides in a two-step process. In the first reaction, the phosphodiester backbone is cleaved at G and a 2′,3′-cyclic phosphate species is formed on the 3′-G ribose; the 5′-carbon of the next nucleotide ribose is not phosphorylated (it is hydroxylated). The enzyme also catalyzes the hydrolysis of ribose 2′,3′-cyclic phosphate to 3′-phosphate. Thus, the predominant RNase T1 products are oligonucleotides with a 5′-hydroxyl on the 5′-end nucleotide, a 3′-end guanosine nucleotide with a 3′-phosphate, and no intervening G nucleotides.

The resulting oligonucleotide fragments are separated by ion-paired reversed-phase UHPLC and detected using ultraviolet absorbance, yielding a characteristic chromatogram, or oligonucleotide map. A visual comparison of reference material and sample oligonucleotide maps may be performed to assess the identity and comparability of samples. The mass spectrometer is plumbed in-line with the UHPLC-UV system. Oligonucleotides are identified by high-resolution LC/MS/MS sequencing, enabling mRNA primary structure mapping.

# Safety

The etiological properties of the mRNA products may be unknown. Avoid ingestion, inhalation, and skin contact with samples and reagents. Consult Safety Data Sheets (SDS), when available, for additional safety information.

# Definitions

| **Table 1. Definitions** | |
| --- | --- |
| **TERM** | **DEFINITION** |
| mRNA | Messenger ribonucleic acid |
| RPLC/UV | Reversed phase liquid chromatography coupled to ultraviolet absorbance detection |
| HPLC | High-performance liquid chromatography |
| UHPLC | Ultra-high-performance liquid chromatography |
| HEPES | 2-[4-(2-hydroxyethyl)piperazin-1-yl]ethanesulfonic acid |
| EDTA | Ethylenediaminetetraacetic acid |
| DS | Drug substance |
| DNA | Deoxyribonucleic acid |
| RNase T_1_ | Ribonuclease T_1_ |
| G | Guanine, Guanosine, Guanosine monophosphate, Guanosine monophosphate as a biopolymer residue |
| m/z | Mass over charge |
| MS | Mass spectrometry, mass spectrometer, m/z spectrum, mass spectrum |
| HRMS | High resolution mass spectrometry, high resolution mass spectrometer |
| Like-for-like replacement | Replacement item can be used from another source without equivalency testing prior to use |
| Equivalent | Replacement item can be used from another source if equivalency is demonstrated prior to use |
| TIC | Total Ion Chromatogram |

# Equipment and Reagents

## Equipment

| **Table 2. Equipment** | | | |
| --- | --- | --- | --- |
| **Equipment** | **Manufacturer/Vendor** | **Description** | **Part Number** |
| Fume hood |  |  |  |
| Pipettes |  | Capable of delivering 2.0 µL to 1000 µL |  |
| Incubating Shaker |  | Capable of heating to 37 °C |  |
| UHPLC system with UV detection |  | HPLC system with quaternary or binary pumps and capable of delivering a gradient and handling pressure of ≥ 600 bar (9000 psi) |  |
| High Resolution Mass Spectrometer |  | e.g. Thermo Orbitrap Eclipse |  |
| Bath Sonicator |  | e.g. Branson 5510 |  |

## Materials

| **Table 3. Materials** | | | |
| --- | --- | --- | --- |
| **Material** | **Manufacturer/Vendor** | **Description** | **Part Number** |
| Isopropanol wipes^a^ | ThermoFisher Scientific | Cleanroom Wipes, Poly-cellulose, 70% IPA | 17444005 |
| Autosampler vial (AS) ^a^ | Waters | Clear Glass 12 x 32 mm Screw Neck Total Recovery Vial, preassembled with PTFE/Silicone Septum 100 pack | 186004631 |
| Pipette tips |  | DNase and RNase free |  |
| Solvent bottles |  | glass |  |
| Graduated cylinders |  | glass |  |
| Greiner Bio-One 0.5mL Polypropylene Microcentrifuge Tubes ^a^ | Fisher | DNase- RNase-free Microcentrifuge Tubes, 0.5 mL | 07-000-693 |
| Reversed phase column | Waters | ACQUITY PREMIER Oligonucleotide C18 Column, 130Å, 1.7 µm, 2.1 x 150 mm | 186009486 |

^a^ Like-for-like replacement materials from another source is acceptable

## Reagents

| **Table 4. Reagents** | | | |
| --- | --- | --- | --- |
| **Reagent** | **Manufacturer/Vendor** | **Description** | **Part Number** |
| Tris(hydroxymethyl)aminomethane Hydrochloride, 1 M, pH 7.5, DNase & RNase free (Tris-HCl, 1 M, pH 7.5) ^a^ | ThermoFisher Scientific | UltraPure™ 1 M Tris-HCI Buffer, pH 7.5 | 15567027 |
| Ethylenediaminetetraacetic acid, 0.5 M (EDTA)^a^ | ThermoFisher Scientific | UltraPure™ 0.5M EDTA, pH 8.0 | 15575020 |
| Nuclease-free water (such as DEPC-treated water) ^a^ | ThermoFisher Scientific | DEPC-Treated Water | AM9906 |
| Purified water with a resistivity of ≥18 mOhms and treated for LC/MS use^a^ | Cartrige for LC-MS use: MilliporeSigma | MilliQ / Sartorius; Cartridge for LC-MS use: LC-Pak® Polisher | Cartridge for LC-MS use: LCPAK0001 |
| 1,1,1,3,3,3-Hexafluoro-2-propanol, LCMS Grade (HFIP) ^a^ | MilliporeSigma | 1,1,1,3,3,3-Hexafluoro-2-propanol, LC-MS Grade | 18127-50ML |
| Triethylamine, LCMS grade (TEA) ^a^ | ThermoFisher Scientific | Covachem LCMS grade Triethylamine-25ML | NC1417016 |
| Methanol, LCMS grade (MeOH) ^a^ | ThermoFisher Scientific | Methanol, Optima™ LC/MS Grade, Fisher Chemical | A456 |
| Thermo RNase T1, 1000 U/µL, 100 kU, Mw 11.2 KDa monomer (Storage buffer: 50 mM Tris, pH 7.4 and 50% v/v glycerol) | ThermoFisher | RNase T1 (Ribonuclease T1) is an endoribonuclease that specifically hydrolyzes RNA phosphodiester bonds at the 3′ end of G bases | EN0541 |
| ^a^ Like-for-like replacement of reagents from another source is acceptable | | | |

# Procedure

## Preparation of Solutions

NOTE: The preparations described here are scalable, provided that changes are proportional. Preparations outside this range may be appropriate but have not been tested.

### **Mobile Phase A (1% HFIP, 0.1% TEA in LC/MS Grade Water)**

#### Prepare in a fume hood.

#### Prepare a 1 L bottle: triple-rinse with LC/MS grade methanol and then triple-rinse with LC/MS grade water.

#### Add 989 mL of purified water to the l L bottle.

#### Using a pipette, add 1 mL TEA to the l L bottle.

#### Using a 10 mL graduated cylinder or a pipette, add 10 mL of HFIP to the l L bottle.

##### For smaller preparations, pipetting is advisable. Given the low surface tension and high density of HFIP, this will drip from the pipette tip; therefore, keep the HFIP bottle close to the top of the cylinder when aspirating/dispensing.

#### Mix by inverting several times. Sonicate the solvent in a bath sonicator for 5 minutes.

#### Mobile Phase A can be stored at room temperature for up to 6 months.

### **Mobile Phase B (1% HFIP, 0.1% TEA in 50% LC/MS Grade MeOH)**

#### Prepare in a fume hood.

#### Prepare a 0.5 L bottle: triple-rinse with LC/MS grade methanol and then triple-rinse with LC/MS grade water.

#### Add 244.5 mL of purified water to the 0.5 L bottle.

#### Add 250 mL of MeOH to the 0.5 L bottle.

#### Using a pipette, add 0.5 mL TEA to the l L bottle.

#### Using a 10 mL graduated cylinder or a pipette, add 5 mL of HFIP to the l L bottle.

##### For smaller preparations, pipetting is advisable. Given the low surface tension and high density of HFIP, this will drip from the pipette tip; therefore, keep the HFIP bottle close to the top of the cylinder when aspirating/dispensing.

#### Mix by inverting several times. Sonicate the solvent in a bath sonicator for 5 minutes.

#### Mobile Phase B can be stored at room temperature for up to 6 months.

### **Needle wash and seal wash solution (10% IPA)**

#### Prepare in a fume hood.

#### Prepare a 1 L bottle: triple-rinse with LC/MS grade methanol and then triple-rinse with LC/MS grade water.

#### Add 900 mL of purified water to the l L bottle.

#### Add 100 mL of IPA to the bottle.

#### Mix by inverting several times.

#### Needle wash and seal wash can be stored at room temperature for up to 6 months.

### **Spike solution (11% HFIP, 1.1% TEA in LC/MS Grade Water)**

#### Prepare in a fume hood.

#### Prepare a 1.5 mL microcentrifuge tube or similar-sized glass vial.

#### Add 1 mL of purified water to the tube.

#### Add 12.51 µL TEA to the tube.

#### Add 125.1 µL of HFIP to the tube.

#### Vigorously vortex before use. The mixture is an emulsion.

#### Spike solution can be stored at room temperature for up to 1 month.

## Preparation of Reference Material and Samples

NOTE: The preparations described here are scalable from 10 ug to 200 ug mRNA, provided that changes are proportional. Preparations outside this range may be appropriate but have not been tested.

### Preparing working surface: wipe down counter with isopropanol wipes.

### The reaction volume is 35 µL for the digestion of 50 µg of RNA. The digest reaction concentrations are:

| **Table 5. Digestion Concentrations and Quantities** | |
| --- | --- |
| **Thing** | **Concentration or Quantity** |
| RNA | 50 µg |
| Tris pH 7.5 | 50 mM |
| EDTA | 20 mM |
| RNase T1 | 2500 U |

### Determine the sample multiplication factor *f*:

### *f* = # of samples (incl. RM) + 0.5

### For example, if set of samples to be analyzed is 3, *f* = 3.5

### Prepare reaction solution:

#### Volume in µL of 1 M Tris pH 7.5 = 1.75 × *f*

#### For example, for a set 3 samples, add 6.13 µL of 1 M Tris.

#### Volume in µL of 0.5 M EDTA = 1.4 × *f*

#### For example, for a set of 3 samples, add is 4.90 µL of 0.5 M EDTA.

#### Volume in µL of 1000 U/µL RNase T_1_ = 2.5 × *f*

#### For example, in a set of 3 samples, add 8.75 µL of 1000 U/µL RNase T_1_.

#### Aliquot 5.65 µL of the reaction solution to glass autosampler vials, one for each sample.

### Prepare each sample as follows:

#### Determine the volume *V_s_* (in µL) of sample equivalent to 50 µg of RNA:

#### *V_s_* = 50 ÷ *C*

#### *C* = the concentration in mg/mL (µg/µL) of the sample

#### For example, if *C* = 2 mg/mL then *V_s_* = 25 µL

#### Add a volume *V_w_* (in µL) of nuclease-free water to the glass autosampler vial designated for the sample:

#### *V_w_* = 29.35 - *V_s_*

#### For example, if *C* = 2 mg/mL, then *V_s_* = 25 µL and *V_w_* = 4.35 µL

### Digest samples.

#### Add the volume *V_s_* determined in 6.2.5.1 of sample to the appropriate autosampler vial. Mix the reaction solution, water, and sample by pipette action.

#### Incubate 1 h at 37 °C with shaking at 300 rpm.

#### If the samples are not to be analyzed immediately, store them at -80 °C for up to 2 months.

### Spike samples just prior to analysis.

#### Vigorously vortex the spike solution before use. The mixture is an emulsion.

#### Add 3.5 µL of spike solution to each sample. Mix by pipette.

#### Do not inject samples stored in the chilled autosampler beyond 72 hours.

## Instrument Parameters

### Set up the Agilent 1290 Infinity II Bio-Inert UHPLC system. Purge solvent lines with appropriate mobile phases. Recommend 3 min each, 3 mL/min for each line, increasing time and flow rate if switching solvents or lines contain significant air.

### Turn on the UV lamp.

### Install the column and set the column heater to 75 °C.

### Set Mobile Phase A and Mobile Phase B to 50% each and flow rate to starting conditions (0.2 mL/min).

### Set pump conditioning for 15 min to remove any microbubbles in the pump heads.

### After pump conditioning, equilibrate column at starting conditions (0.2 mL/min, mobile phase B at 1%) for 15 minutes.

#### **NOTE: To avoid over-pressurizing, ensure the column has come to temperature prior to equilibrating to initial conditions.**

#### Table 6 lists the chromatographic conditions for the HPLC system. Table 7 lists the gradient conditions for the HPLC system.

| **Table 6. Chromatographic Conditions** | |
| --- | --- |
| **Parameter** | **Materials and Conditions** |
| Column | ACQUITY PREMIER Oligonucleotide C18 Column, 130Å, 1.7 µm, 2.1 x 150 mm |
| Mobile Phase A | 1% HFIP, 0.1% TEA in water |
| Mobile Phase B | 1% HFIP, 0.1% TEA in 50% MeOH |
| Column Temperature | 75 ± 2 °C |
| Sample Temperature | 5 ± 3 ºC |
| UV Detection / Bandwidth | 260 nm / 4 nm |
| Reference Wavelength / Bandwidth | 360 nm / 20 nm |
| Peak Width | > 0.1 min (2 s response time) (2.5 Hz) |
| Post Time | Off |
| DAD Spectrum (if available) | 190 to 400 nm, step 2 nm |
| Margin for Negative Absorbance | 100 mAU |
| Slit | 4 nm |
| Run Time | 280 minutes |
| Injection Volume | 3.1 μL (4 µg) |
| Elution | Gradient |

| Table 7: Binary Pump System Gradient | | | |
| --- | --- | --- | --- |
| **Time (minutes)** | **Flow Rate**  **(mL/min)** | **% Mobile Phase A** | **% Mobile Phase B** |
| Initial | 0.20 | 99 | 1 |
| 5 | 0.20 | 99 | 1 |
| 200 | 0.20 | 83 | 17 |
| 260 | 0.20 | 65 | 35 |
| 265 | 0.20 | 0 | 100 |
| 267 | 0.20 | 0 | 100 |
| 268 | 0.20 | 100 | 0 |
| 269 | 0.20 | 0 | 100 |
| 270 | 0.20 | 99 | 1 |
| 290 | 0.20 | 99 | 1 |

### It is recommended to plumb flow to the mass spectrometer using a flow splitter.

#### The 0.2 mL/min flow is split using a PEEK low dead-volume splitter: One-quarter of the flow is directed to the UV detector and three-quarters is directed to the divert valve and then to the MS. The flow rate is adjusted by changing the length of the 0.005” PEEK tubing connecting the UV detector to solvent waste.

#### Exemplary Global Parameters, including source conditions, for the MS will vary depending on the flow rate; Table 8 provides the source conditions for a 0.15 mL/min flow.

### At least one hour prior to acquisition, switch the mass spectrometer to operate with the polarity set to negative mode.

### After one hour of thermal equilibration, verify the mass spectrometer is calibrated using tune mix solution as specified by the instrument operation method. Calibrate the instrument if needed.

### Exemplary Scan Parameters for the Thermo Orbitrap Eclipse are given in Table 9. Other HRMS instruments will require different parameters, which can be optimized by tuning for negative mode acquisition using the appropriate tune mix. The three-segment acquisition has been optimized for the best fragmentation and spectrometry of oligonucleotides based on their size.

| **Table 8. Thermo Orbitrap Eclipse Mass Spectrometer Global Settings** | | |
| --- | --- | --- |
| Use Ion Source Settings from Tune | False | |
| Method Duration (min) | 280 | |
| Ion Source Type | H-ESI | |
| Spray Voltage: Positive Ion (V) | 3400 | |
| Spray Voltage: Negative Ion (V) | 2700 | |
| Sheath Gas (Arb) | 40 | |
| Aux Gas (Arb) | 10 | |
| Infusion Mode (LC) | False | |
| Sweep Gas (Arb) | 0 | |
| Ion Transfer Tube Temp (°C) | 320 | |
| Vaporizer Temp (°C) | 300 | |
| APPI Lamp | Not in use | |
| FAIMS Mode | Not Installed | |
| Divert Valve A | Time (min) | Position |
|  | 0 | Waste |
|  | 3 | MS |
|  | 255 | Waste |
| Application Mode | Peptide | |
| Pressure Mode | Standard | |
| Default Charge State | 2 | |
| Advanced Peak Determination | True | |
| Xcalibur AcquireX enabled for method modifications | False | |

| **Table 9. Thermo Orbitrap Eclipse Mass Spectrometer MS and MS/MS Settings** | | | |
| --- | --- | --- | --- |
|  | **Experiment (Segment) 1** | **Experiment (Segment) 2** | **Experiment (Segment) 3** |
| Experiment Name | MS | | |
| Start Time (min) | 0 | 10 | 240 |
| End Time (min) | 10 | 240 | 260 |
| Cycle Time (sec) | 2 | | N/A |
| Desired minimum points across the peak | 9 | | N/A |
| MSn Level | 1 | | |
| Use Wide Quad Isolation | True | | |
| Detector Type | Orbitrap | | |
| Orbitrap Resolution | 120K | | |
| Mass Range | Normal | | |
| Scan Range (m/z) | 100-1000 | 400-2000 | 700-2000 |
| Maximum Injection Time (ms) | 50 | | 300 |
| AGC Target | 400000 | | 1000000 |
| Normalized AGC Target | 100% | | 250% |
| Microscans | 1 | | 5 |
| RF Lens (%) | 30 | | 50 |
| Maximum Injection Time Type | Auto | | Custom |
| Use ETD Internal Calibration | False | | |
| DataType | Profile | | |
| Polarity | Negative | | |
| Source Fragmentation | False | | |
| Enhanced Resolution Mode | Off | | |
|  | **Experiment (Segment) 1** | **Experiment (Segment) 2** |
| MS/MS Selection | | |
| Filter IntensityThreshold | | |
| Relative Intensity Threshold | 0 | |
| Intensity Filter Type | IntensityThreshold | |
| Minimum Intensity | 50000 | |
| Maximum Intensity | 1E+20 | |
| Filter ChargeState | | |
| Include charge state(s) | 2-20 | |
| Include undetermined charge states | False | |
| Filter DynamicExclusion | | |
| Exclude after n times | 1 | |
| Exclusion duration (s) | 6 | |
| Mass Tolerance | ppm | |
| Mass tolerance low | 8 | |
| Mass tolerance high | 8 | |
| Use Common Settings | False | |
| Exclude isotopes | True | |
| Perform dependent scan on single charge state per precursor only | False | |
| Data Dependent Properties | | |
| Data Dependent Mode | Cycle Time | |
| Scan Event 1: MS/MS | | |
| Scan | ddMSnScan | |
| Desired minimum points across the peak | 9 | |
| MSn Level | 2 | |
| Isolation Mode | Quadrupole | |
| Isolation Offset | Off | |
| Isolation Window | 1.5 | |
| Enable Auto PTR Windows | False | |
| Reported Mass | Original Mass | |
| Multi-notch Isolation | False | |
| Scan Range Mode | Auto | |
| FirstMass | 100 | |
| Scan Priority | 1 | |
| ActivationType | HCD | |
| Collision Energy Mode | Stepped | |
| Collision Energies (%) | 17,21,25 | |
| Detector Type | Orbitrap | |
| Orbitrap Resolution | 30K | |
| Scan Range (m/z) | 150-2000 | |
| Maximum Injection Time (ms) | 300 | |
| AGC Target | 125000 | |
| Inject ions for all available parallelizable time | False | |
| Normalized AGC Target | 250% | |
| Microscans | 1 | |
| Maximum Injection Time Type | Custom | |
| Use ETD Internal Calibration | False | |
| DataType | Centroid | |
| Polarity | Negative | |
| Source Fragmentation | False | |
| Enhanced Resolution Mode | Off | |

## Sample Sequence

### Prior to beginning a sample sequence, equilibrate per 6.3.6.

### Analysts may make 1 intermediate reference material injection after every 10 or fewer sample injections, if desired.

#### Do not inject samples stored in the autosampler >48 hours.

#### The injection volume delivers 4 µg RNA on column.

## HPLC System Shutdown

### After the injection sequence is complete, turn off the column heater and UV lamp.

### Flow 100% solvent B (50% MeOH) onto the column for 10 minutes at 0.1 mL/min.

### Column can be stored in the column wash solution between analyses. For long-term storage, store the column in 90% LC/MS grade methanol or 90% LC/MS grade acetonitrile with no TEA nor HFIP.

### Prior to beginning the next sample set, ensure sufficient column re-equilibration time at initial conditions.

# Analysis of LC/MS/MS Data

## Characterize the 3´ Poly(A)-Tail using Protein Metrics Byos (see Appendix Attachments F1-F5 for detailed guidance)

### Load .raw data file(s) into Byos

### Select processing parameters

### Run processing method

### As necessary to characterize each region of interest (e.g. the A30 and L70 region for platform modRNA constructs), add peaks, adjust retention times, and deconvolve mass spectra

### Export observed zero-charge neutral mass spectra for each peak and create appropriate figure(s) for report

### Export observed zero-charge neutral mass tables for each peak and create appropriate mass tables(s) for report

## Oligonucleotide Mapping Readout

### An annotated LC/UV chromatogram, will all conspicuous peaks labeled up to and including the poly(A) tail features.

#### Use discretion as to what is a “conspicuous” LC/UV peak. Any LC/UV feature can be identified and annotated using the method described here. Whether small features should be is a fit-for-purpose decision for the analyst and project team.

#### In a comparability experiment, only the top chromatogram should be annotated. It is up to the project team to decide which batch is the appropriate top chromatogram.

### A table of identified observed masses, based on MS and MS/MS fragmentation data matched to theoretical data.

#### MS matching requires a ≤ 5 ppm error for identification.

#### A good MS/MS fragmentation match is one in which all major fragments are identified, and the complete sequence may be inferred from fragment ions containing the 5ꞌ or 3ꞌ ends (not internal fragments).

#### The poly(A)-tail-containing oligonucleotides can be identified by MS matching.

#### The guanosine nucleotide may be identified by MS matching but can also be identified by retention time and comparison to historical data.

### Sequence coverage calculation is based on the table of identified observed masses.

### Other software may be used to achieve heightened characterization; the sections below detail the use of Thermo BioPharma Finder and Excel macro spreadsheet written to augment and check the BioPharma Finder results.

### Data analysis is a multistep process using several software, as illustrated in the flowchart below. Software tools are marked by blue nodes. Orange nodes are the input and outputs of software tools. The MasterList.xlsx node is marked yellow because it is both a software output and a means to update the observed sequence coverage.


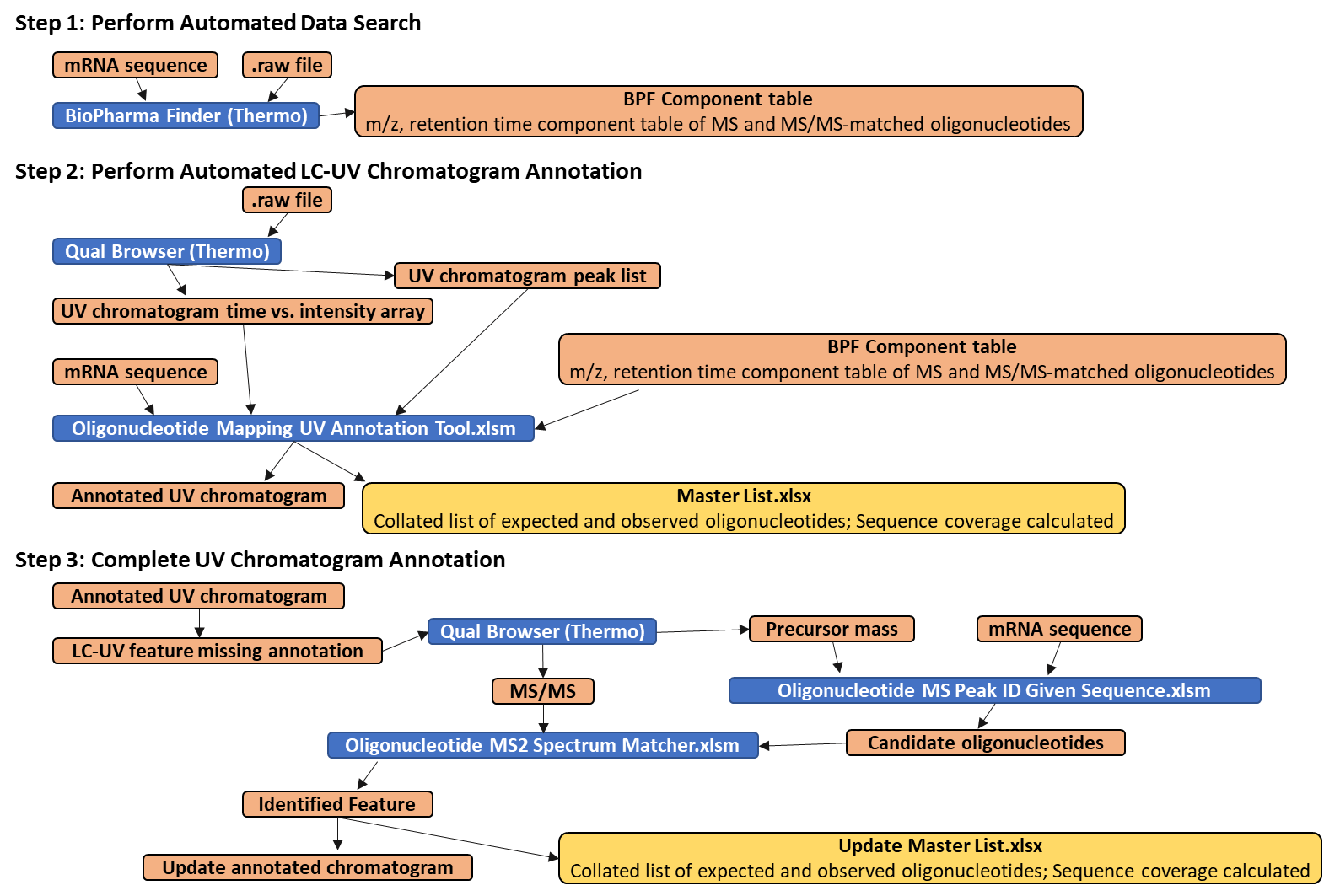

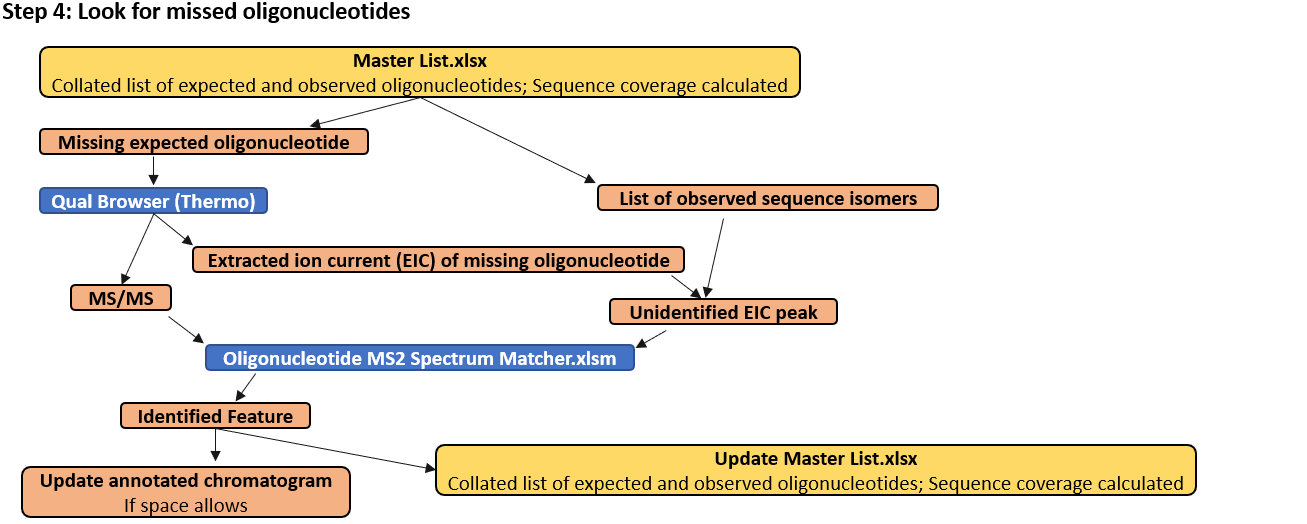

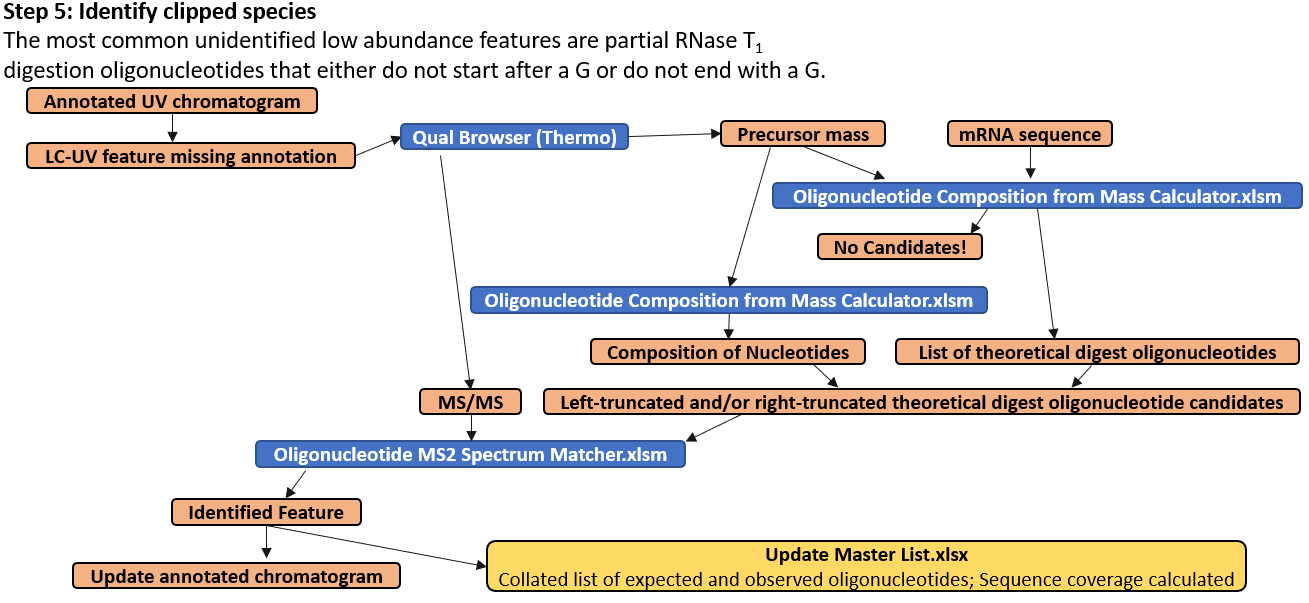


## Step 1: Automated Data Search

### Use Thermo BioPharma Finder Version 5.1 or later with the Oligonucleotide Module.

### Enter a new theoretical oligonucleotide sequence into Biopharma Finder.

#### Select Sequence Manager.

#### Select New Oligonucleotide.

#### Select Import Fasta File and find the appropriate sequence.

##### For RNA, use a “U” in place of “T” for uridine.

##### For modRNA, use a “V” or other non-DNA base to designate N1-methylpseudouridine.

#### The Category for the Target Oligonucleotide is “Sequencing”.

#### Use the Building Block and Variable Modification Editor as needed to properly construct the sequence.

##### For modRNA: Add a “Base” Oligo Building block using the symbol specified in 7.3.2.3.2; its formula is C_5_H_6_N_2_O_2_ with monoisotopic and average masses of 126.043 and 126.11, respectively.

##### Add the 5ꞌ-cap 5ꞌ Terminal modification if appropriate (some RNA molecules aren’t capped). For a 5ꞌ-cap Cap-1 structure, its formula is C_13_H_22_N_5_O_14_P_3_ with monoisotopic and average masses of 565.038 and 565.26, respectively.

##### Add the cyclic phosphate variable modification as a 3ꞌ Terminal modification. Its formula is -H_2_O with monoisotopic and average masses of -18.011 and -18.02, respectively.

#### Use the Edit Sequence section to add the appropriate 5ꞌ Terminal modification using the modification defined in 7.3.2.5.2. First select chain 1, then the modification in the 5ꞌ Terminal drop-down field.

#### Assign Variable Modifications:

##### 5ꞌ Terminal: Phosphorylation. This might arrive from a non-specific cleavage, incomplete transcription, or degradation event.

##### 3ꞌ Terminal: Cyclic phosphate as defined in 7.3.2.5.3.

##### There is no recommendation for Oligo Variable Modification. Losses of bases and depurination/depyrimidination are detectable by mass spectrometry, but typically are of very low abundance are not part of the RNA mapping readout scope. Add modifications as desired.

#### Set the Max. Num. of Modifications = 1

### Perform Oligonucleotide Analysis.

#### Select one or multiple .raw files. For multiple files, make the Reference Condition the raw file that is to be the top chromatogram.

##### For multiple files - select “Multiconsensus” to group components and identifications from multiple batches into a single result file (recommended for comparability analyses)

##### For multiple files – select “Batch Process” to batch process using a single method but with individual result files, independent of each other.

#### Select the Oligonucleotide Sequence.

#### Select the Basic Default Method.

##### If a uniquely-named platform method has already been created, using parameters as specified in this protocol, user may select that method instead.

#### Name the experiment in “Experiment Name”.

#### Select “Start Processing” to begin search

##### Uncheck the “Enable Automatic Parameter Values” to disable Biopharma Finder from reading result file(s) and recommending parameters (recommended when running the instrument method as specified).

##### Check the “Enable Automatic Parameter Values” to enable Biopharma Finder to read result file(s) and recommend parameters (recommended if performing significant alternations to the instrument method).

##### Select “Yes” when prompted that “The component detection interface now includes an improved visualization feature for viewing the Absolute MS Signal Threshold value. We recommend that you optimize this threshold value in the method before processing the experiment. Do you want to review and optimize the method settings?”

##### Alternatively, a user may “Start Processing” with “Enable Automatic Parameter Values” unchecked, which will run the exact parameters specified by the method without an option to review the parameters.

#### See Tables 11 and 12 for recommended Biopharma Finder method search parameters.

| Table 11: Recommended BioPharma Finder RNase T_1_ Digest Sequencing Method Parameters | |
| --- | --- |
| **Component Detection Parameters** | |
| **Parameters Name** | **Selected Parameter(s)** |
| Task to Perform | Find All Ions in the Run |
| Absolute MS Signal Threshold (MS Noise Level * S/N Threshold) | 1E5 |
| MS Noise Level | 4000 |
| S/N Threshold | 25 |
| Beginning Peak Width (min) | 1.34 |
| Typical Chromatographic Peak Width (min) | 1.34 |
| Ending Peak Width (min) | 1.34 |
| Maximum Chromatographic Peak Width (min) | 13.19 |
| Use Restricted Time | No |
| Time Limits | 0 to 260 |
| Relative MS Signal Threshold (% of base peak) | 1 |
| Relative Analog Threshold (% of highest peak) | 1 |
| Width of Gaussian Filter (represented as 1/n of chromatographic peak width) | 3 |
| Minimum Valley to be Considered as Two Chromatographic Peaks (%) | 80.00% |
| Minimum MS Peak Width (Da) | 1.2 |
| Maximum MS Peak Width (Da) | 4.2 |
| Mass Tolerance (ppm for high-res or Da for low-res) | 4 |
| Maximum Retention Time Shift (min) | 0.1 (Note – look at your data) |
| Maximum Mass (Da) | 30000 |
| Mass Centroiding Cutoff (% from base) | 15 |
| **Sequence Parameters (set in Sequence Manager – see Appendix E1-4)** | |
| **Parameters Name** | **Selected Parameter(s)** |
| Static Modifications | (5'Term) Cap (e.g. C13H22N5O14P3 for modRNA) – See Appendix |
| Variable Modifications | Phosphorylation(5'Term), Dephosphorylation(5'Term), Cyclic_P(3'Term) |
| **Identification Parameters** | |
| **Parameters Name** | **Selected Parameter(s)** |
| Search by Full MS Only | No |
| Use MS/MS | Use All MS/MS |
| Maximum Oligonucleotide Mass | 30000 |
| Mass Accuracy (ppm) | 5 |
| Minimum Confidence | 0.8 |
| Maximum Number of Modifications for an Oligonucleotide | 1 |
| Enable Mass Changes for Unspecified Modifications | False |
| Enable Residue Deletion | False |
| RNase | RNase T1 (G-) |
| Custom Specificity | False |
| Phosphate Location | 3'-linear |
| Specificity Level | High |

| Table 12: Recommended Supplemental BioPharma Finder Search Parameters | |
| --- | --- |
| **Component Detection Parameters** | |
| **Parameters Name** | **Selected Parameter(s)** |
| All parameters | Same as in Table 11 |
| **Sequence Parameters (set in Sequence Manager – see Appendix E1-4)** | |
| **Parameters Name** | **Selected Parameter(s)** |
| All parameters | Same as in Table 11 |
| **Identification Parameters** | |
| **Parameters Name** | **Selected Parameter(s)** |
| RNase | Nonspecific |
| Specificity | N/A |
| Custom Specificity | False |
| Phosphate Location | 3'-cyclic (supplemental search 1), 3'-linear (supplemental search 2) |
| Specificity Level | High |
| All other parameters | Same as in Table 11 |

### Upon saving the method the BioPharma Finder analysis commences; it should complete within 1 h. If it doesn’t stop the queue and adjust the MS Noise Level and S/N Threshold to increase the Absolute MS Signal Threshold, then re-execute.

### In the Process and Review tab, right-click on the Results table and Export All Components to an Excel Workbook. This is the BPF Component Table.

## Step 2: Automated LC/UV Chromatogram Annotation

### Open the Oligonucleotide Mapping UV Annotation Tool v15.xlsm spreadsheet. In the Xcalibur Peak List worksheet of this spreadsheet, highlight columns A-G and press the delete key. This will clear previous data without deleting columns. On the BPF Component Table worksheet of the spreadsheet, select all by clicking on the indicated select-all box: . Delete all contents with the delete key. On the Oligo Input worksheet of the spreadsheet, select column J and delete its contents with the delete key. On the Chromatogram XY Input worksheet of the spreadsheet, select columns A and B and delete their contents with the delete key.


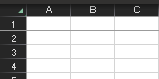


### Open the BPF Component Table. Select all contents by Ctrl-A or by clicking the select-all box as above. Copy all contents. Paste in cell A1 of the BPF Component Table worksheet of the Oligonucleotide Mapping UV Annotation Tool v12.xlsm spreadsheet:
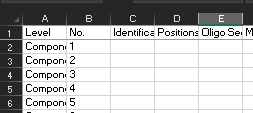


### Open the .raw file with Thermo Qual Browser (Freestyle may also be used).

### Export the UV chromatogram vs. intensity array: right-click on the UV chromatogram and Export Clipboard (Chromatogram). Paste this on the Chromatogram XY Input worksheet of the Oligonucleotide Mapping UV Annotation Tool v12.xlsm spreadsheet, in columns A and B:
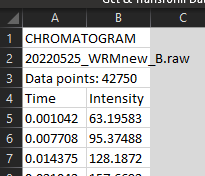


### In Qual Browser, set the Peak algorithm to ICIS using the dropdown Actions🡪 Peak Detection🡪Set Peak Detection Algorithm and Detect in this Plot. Display the Info Bar, if it is not displayed, using the dropdown View. In the Info Bar, the
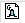
tab displays the ICIS peak detection settings. Set the Peak Parameters Baseline window to 10, Area noise factor to 1, and Peak noise factor to 1. These settings ensure that every UV peak and partial peak is tabulated, though the integrated areas will not be accurate, as this example shows:
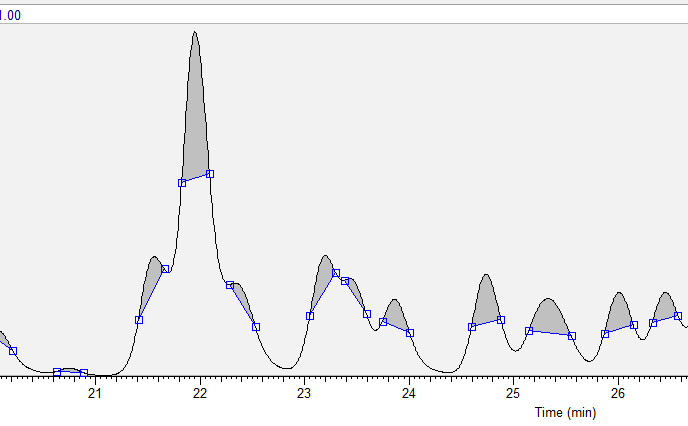


### Export the peak list: right-click on the UV chromatogram and Export Clipboard (Peak List). Paste this on the Xcalibur Peak List worksheet of the Oligonucleotide Mapping UV Annotation Tool v12.xlsm spreadsheet, in columns A-G:


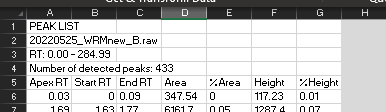


### Open the .fasta file used in 7.3.2.3. Copy the header and sequence and paste in the Oligo Input worksheet of the Oligonucleotide Mapping UV Annotation Tool v12.xlsm spreadsheet, in column J:


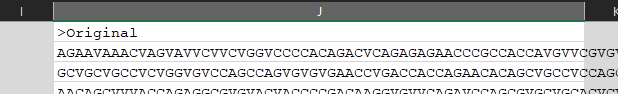


### Fill in the appropriate nucleotide residue information on the Oligo Input worksheet. The masses account for the base, ribose, and phosphate minus water. The absence of water conveys the bio-polymerization of the nucleotide. For example:


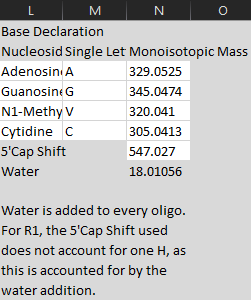


### In the Oligo Input worksheet, fill the any modifications types BioPharma Finder reported in the BPF Component table that are relevant to the analysis. Any putative modications that do not explain the observed masses to within 5 ppm or that do not have supporting MS/MS information are worthless and should not be included on this list. A typical modificaiton list:


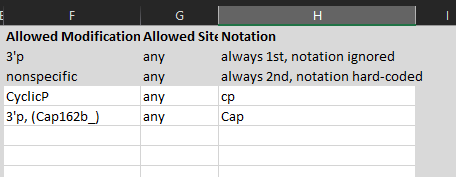


### Fill in the desired settings for the chromatogram segment to be annotated in the Xcalibur Peak List worksheet. The *Start Time* and *End Time* values define the segment. The *UV base peak area % threshold in window* can be used to cull small LC/UV peaks from annotation, but note that the integration settings above are not accurate (0 is thus appropriate in this case). The *TIC base peak intensity % threshold in window* can be used to cull any BPF Component Table ions that do not have sufficient intensity, no matter their identification. The *Other Thresholds* present MS and MS/MS quality parameters that may be used to pass identifications to LC/UV annotation. *Use nonspecifics for coverage?* toggles inclusion of accepted IDs that don’t end with or didn’t start after a G residue in the calculation of sequence coverage (“clip” species). *% base peak threshold in UV peak* sets the lowest accepted ion intensity for the annotation of an associated UV peak. *% base peak threshold in UV peak, but for small things* sets the the lowest accepted ion intensity for the annotation of an associated UV peak for small UV peaks, which is defined by *What is a small UV peak (%)*. *Disable Annotation of Un-ID’d Peaks* toggles off the annotation of UV peaks with observed masses when an ID is not found for the UV peak. *Major Time unit*, *Minor Time unit*, *Baseline shift %*, *Plot height (inches)*, and *Plot width (inches)* parameterize the annotated chromatogram plotting in PowerPoint. *Only Calculate Coverage?* may be toggled to only calculate sequence coverage without creating an annotated plot. *The Peak alignment time sift to add to BPF peaks to match UV apex* is discussed below. A 17.6-56 min segment example:


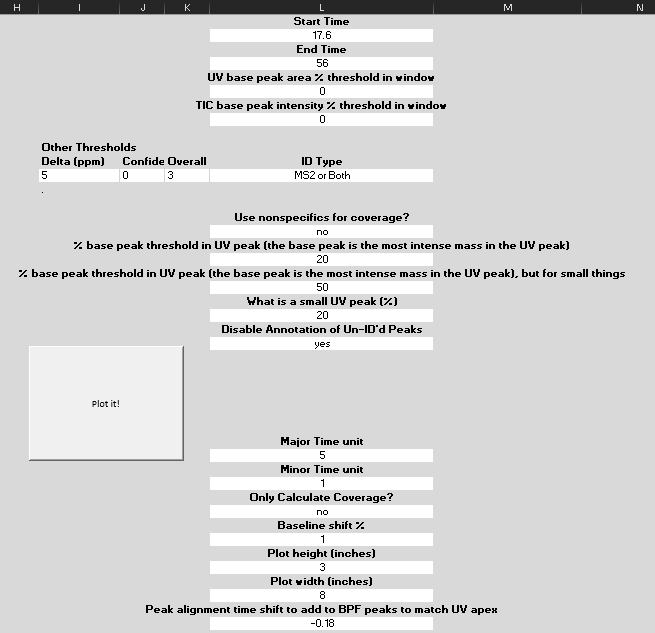


### The *The Peak alignment time sift to add to BPF peaks to match UV apex* is found by noting the major m/z ion in the spectrum summed across a conspicuous LC/UV peak, looking this ion up in the BPF Component Table, and subtracting the BPF Component Table retention time of the ion from the UV peak retention time. Any LC/UV peak suffices. An example:


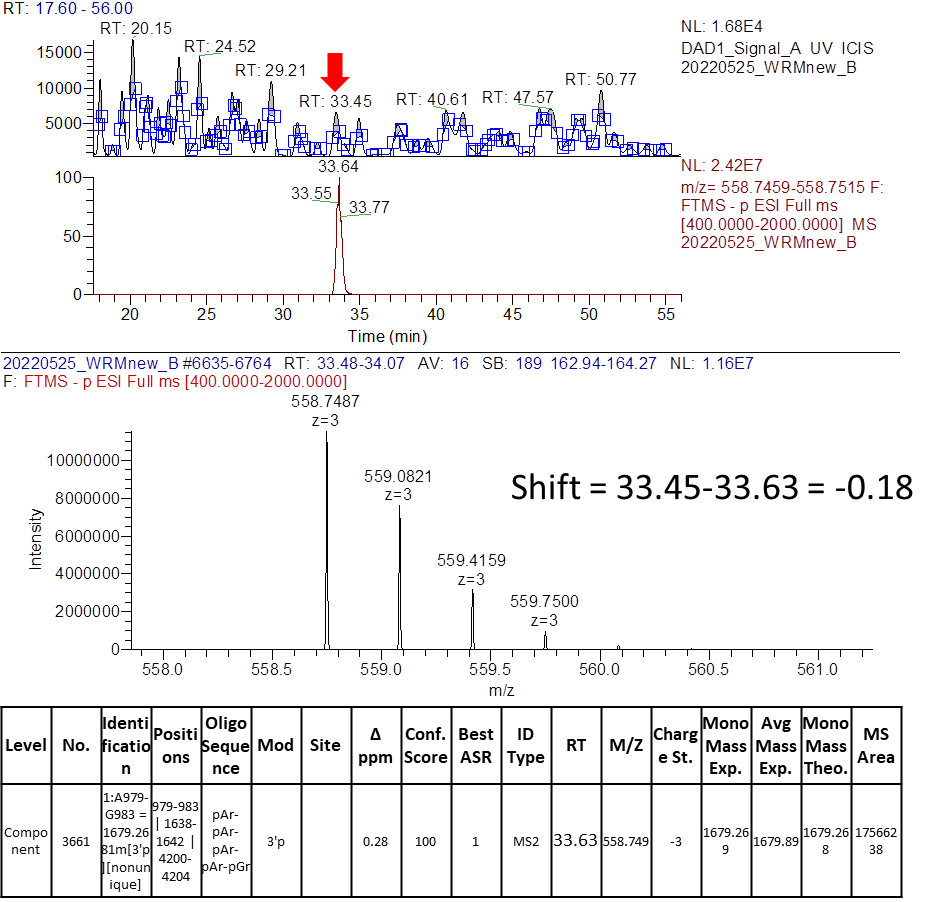


### Execute the IDUVPeaksMakePeptideTable macro by clicking the *Plot it!* button on the Xcalibur Peak List worksheet. Several things are created.

#### The Reference Sequence Coverage worksheet shows which portions of the construct sequence are observed in the time window specified. The sequence coverage in this figure is also calculated:


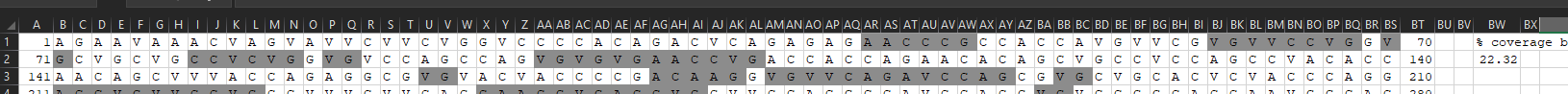


#### The Master List worksheet tabulates oberved oligonucleotides in the context of a theoretical list of all possible RNase T_1_ digest product oligonucleotides of the given construct; is is discussed below.

#### The Xcalibur UV Peaks BPF Matched worksheet documents which observed ions were mapped to UV peaks.

#### The BPF Comp Table UV Matched worksheet documents which UV peaks were mapped to observed ions.

#### The Output Plot worksheet contains the annotated chromatogram, generated from the data on the Plot Data worksheet:


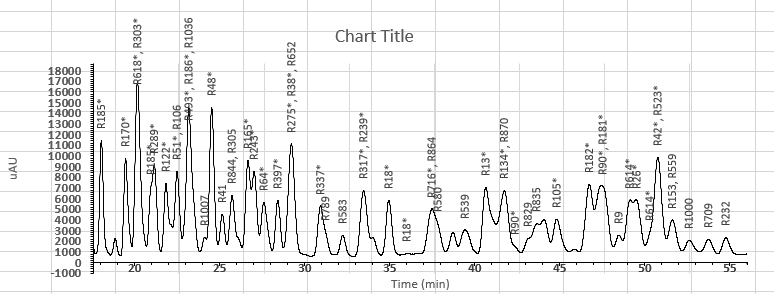


#### The Output Plot worksheet plot was by the macro copied to a new PowerPoint file, with the UV peak annotations placed as text objects on top of the chromatogram figure:


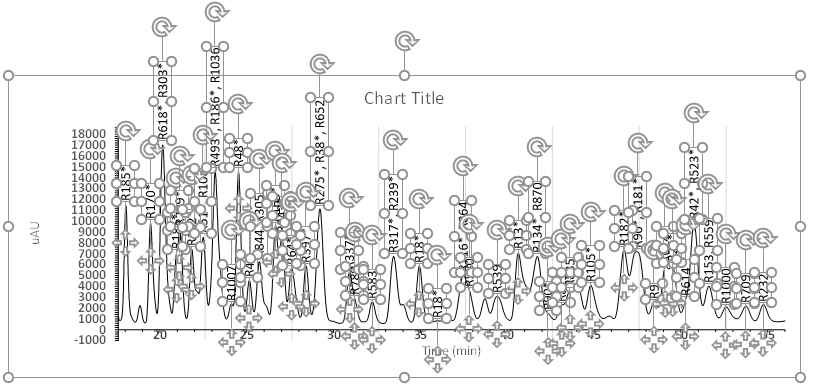


### Extract a high-resolution figure of the LC/UV chromatogram. In Qual Browser, turn off the retention time and peak integration annotations. In the dropdown Edit🡪Copy Special, select Current Cell and an appropriate Output Size in Inches:


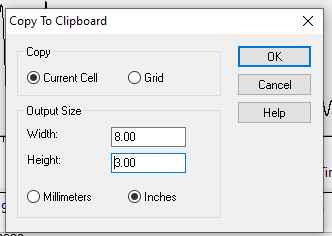


### Paste in a new PowerPoint presentation. Resize the object to the desired dimention. Right click on the imported picture and Ungroup it. A warning pops up; yes, you do want to convert it to a Microsoft Office drawing object. Ungroup the objects again, up to 3 times, to ensure object is free of association. Delete the invisible box overlay objection, which is the largest object in the figure: click on white space and press the Delete key. Other things that can be deleted for the final image are selected in this figure:


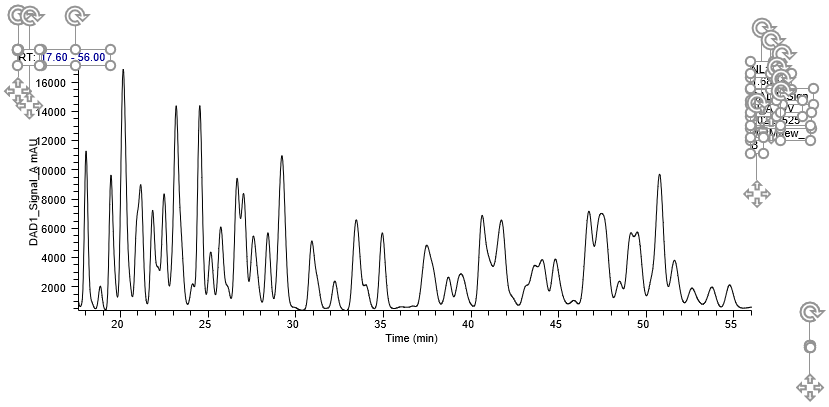


### Right click on the UV trace object and select Format Shape. Change the color to red (or something not black) and the width to 0.75 pt:


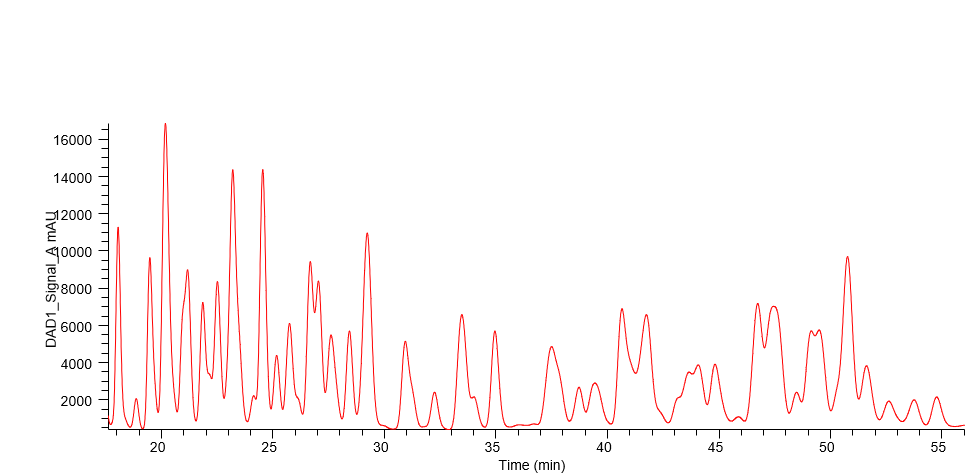


### In the PowerPoint presentation created by Oligonucleotide Mapping UV Annotation Tool v12.xlsm , group the chromatogram picture and text objects. Copy the grouped image and past it over the Qual Browser-exported chromatogram figure:


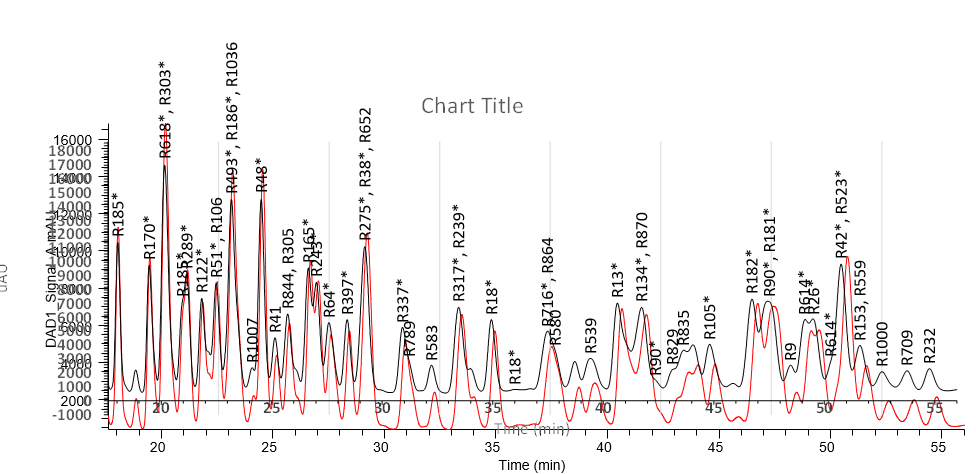


### Adjust the location, width, and height of the Excel macro-generated grouped image to overlay the black trace with the red trace and align the peak annotations over the appropriate red LC/UV feature. In this example, the starting Format Picture Size Height and Width settings were 3.34” and 8”, respectively; the ending settings are 4.5” and 8.08”. The resultant properly overlayed figure:


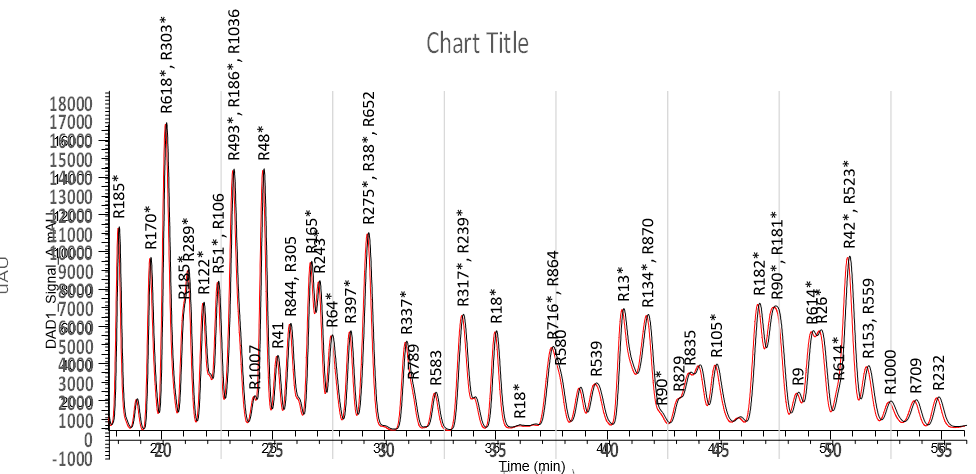


### Ungroup the overlayed Excel macro-generated grouped image. Select the Excel macro-generated chromatogram image and delete it. Right click on the red chromatogram trace and change its color to black. The resulting automated annotation figure:


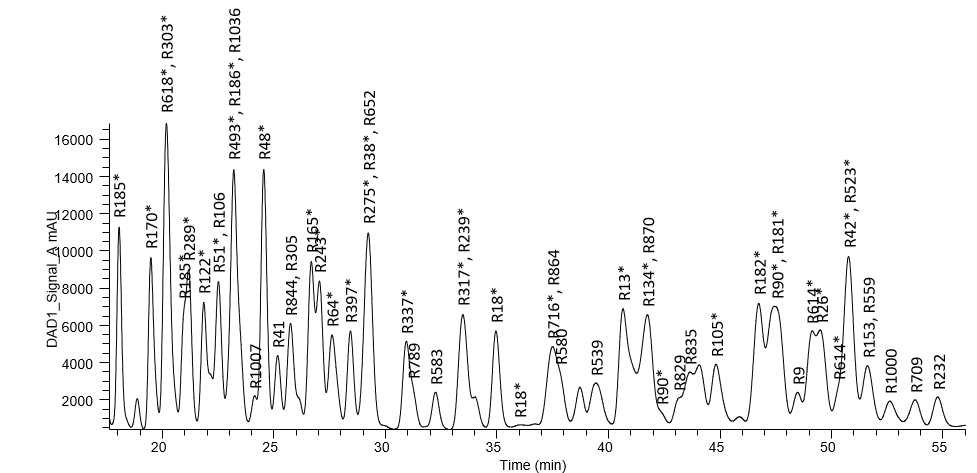


### Arrange annotation text boxes in PowerPoint as desired for clarity.

### Sometimes, especially for shorter sequences, an oligonucleotide originates from more than one instance in the construct sequence. That is, there are more than one loci in the construct that will produce the identical product oligonucleotide from RNase T_1_ digestion. In this case the first instance from the 5′ end is annotated in the UV chromatogram with an asterisk.

## Step 2b: Repeat Step 2 for all desired time segments

## Step 2c: Create the Master List of oberved oligonucleotides

### Re-execute the Oligonucleotide Mapping UV Annotation Tool v12.xlsm Plot it! macro with the *Start Time* set to 0 and *End Time* to 1000 (or time exceeding the LC method), and *Only Calculate Coverage?* as yes. A “Master List” worksheet is created. It is the 10^th^ worksheet of the spreadsheet; click the indicated bottom-left-corner triangle to advance to this worksheet if it is not observed in the available worksheet tabs:
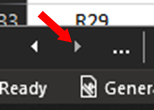
. The Master List worksheet lists all BioPharma Finder-observed oligonucleotides meeting the criteria specified in the Xcalibur Peak List worksheet, and also lists all theoretical RNase T_1_ digest oligonucleotides that were not observed (“missed” oligonucleotides). Observed and missed oligonucleotides are presented in a 5′-to-3′ construct context but can be sorted or filtered as desired; to return the sorting to the original context simply Sort on the “Sort" column from low to high. If desired, to fill in the “Annotated in UV?” column re-execute the Plot it! macro with *Only Calculate Coverage?* set as no.

### The best practice is to copy the Filing Mass Table worksheet data to a new Excel spreadsheet, so that this worksheet is not overwritten by re-execution of the Plot it! macro. Right-click on the Master List tab; select Move or Copy, assign it to a new book (new spreadsheet), click on Create a copy, and click OK:


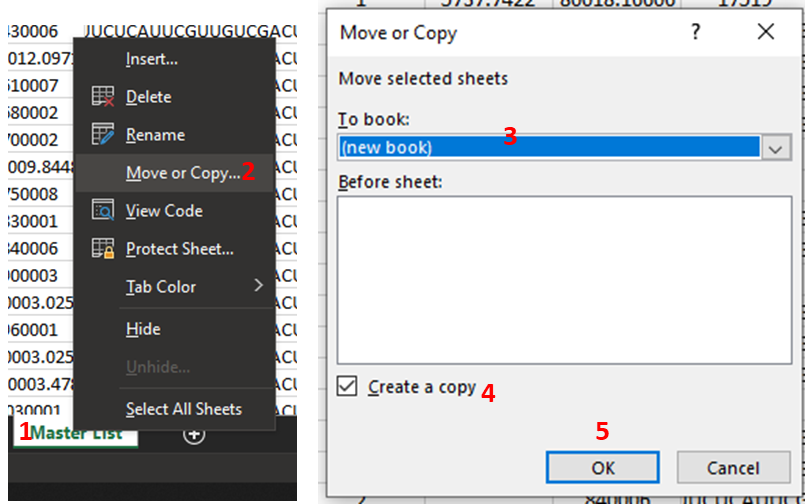


### In the following steps this Master List will be augmented with new observed masses. The MasterList spreadsheet should be used to calculate sequence coverage as new observations are added, and to determine the final sequence coverage. The final sequence coverage, displayed to the right of the Master List table, is dynamically calculated by the formulae in columns G, H, I, the right-most-contiguous column (for one sample, column M), and the presence/absence of number entries in the Observed Mass column of the 1^st^ sample (column J):


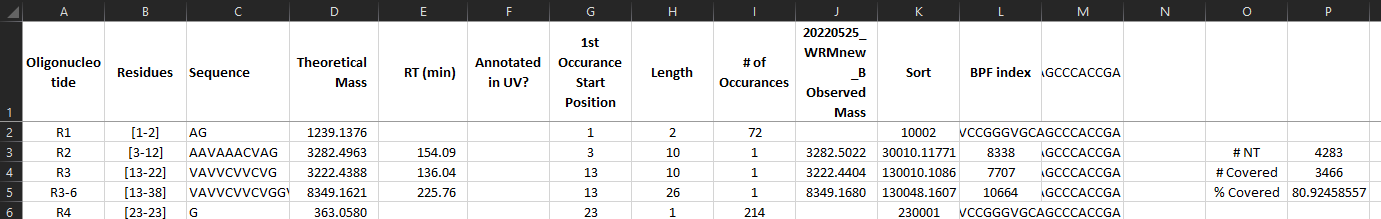


## Step 3: Complete UV chromatogram annotation

### For each time segment, mark un-annotated LC-UV features. Five conspicuous features are noted in this example:


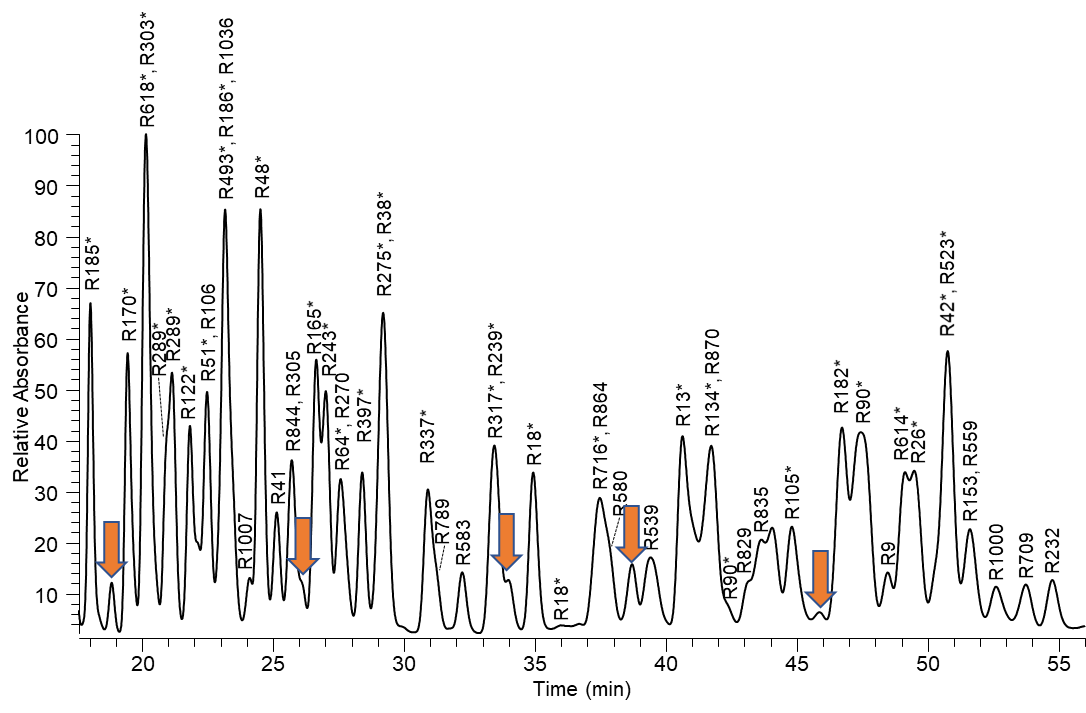


### For each un-annotated LC-UV feature, sum across the LC/MS and determine the ions associated with the UV peak by extracted ion chromatogram. In the following and subsequent workup Thermo Qual Browser is used. Thermo Freestyle or other software may be similarly used though the means for summation and spectrum extraction may be differ.


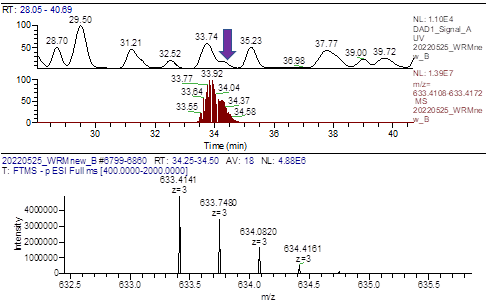


### Open the Oligonucleotide MS Peak ID Given Sequence v9.xlsm spreadsheet. On the Choose Sequence worksheet, enter and select the appropriate sequence. The sequence must be in a single cell, in column D. Fill in appropriate meta information in columns A & B as desired. Select the sequence by marking it in the same row, in column E, with any character. In this way more than one sequence can be stored to prime the spreadsheet for multiple applications:


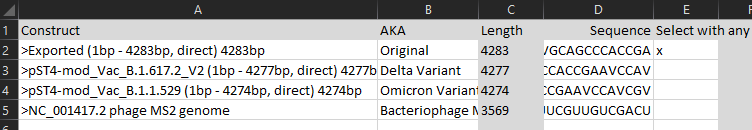


### In the Input worksheet, columns I-L, ensure the same symbol and appropriate masses are entered for the four nucleotides of the sequence. The nucleotide-as-residue mass is the nucleotide monophosphate mass minus water. In columns N-Q, declare the possible 5′ and 3′ construct end masses that may be considered. Internal oligonucleotide digestion products are coded to have a 5′-end mass of 17 Da (HO-) and a 3′- mass of 1 Da (-H):


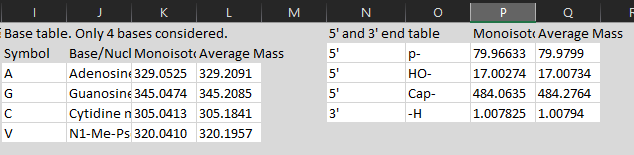


### The screenshot above shows masses to 4 digits past the decimal, but the values in Excel are stored with more accuracy. For the monoisotopic mass, in all cases use the full NIST mass (<https://physics.nist.gov/cgi-bin/Compositions/stand_alone.pl?ele=&all=all>) for each element. For example, adenosine monophosphate as a residue has the elemental composition C_10_H_12_N_5_O_6_P. Its monoisotopic mass is 10×12 + 12×1.00782503223 + 5×14.00307400443 + 6×15.99491461957 + 30.9737619984 = 329.05252012475.

### In the Input worksheet, use the calculator in cells N11 and N12 to de-charge the observed ion. “CS” is negative charge state:
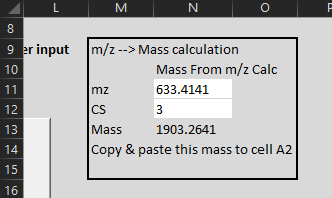
. Copy and paste the mass in cell N13 into cell A2.

### On the Input worksheet, in columns D-F, parameterize the search for mass tolerance and type of endonuclease digestion. Only single base motifs may be considered. A 5 ppm search of RNase T_1_ digest products is thus:


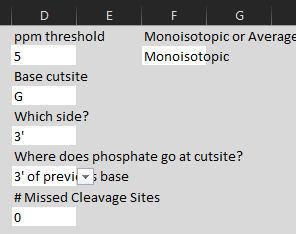


### On the Input worksheet, click the Execute Mass Match Macro button. Four output worksheets are populated. The Theoretical Masses worksheet lists all possible RNase T_1_ digest oligonucleotides:


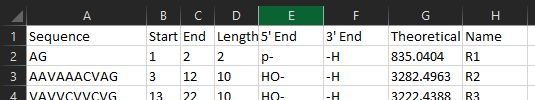


### The Matches worksheet provides a detailed list of matches:
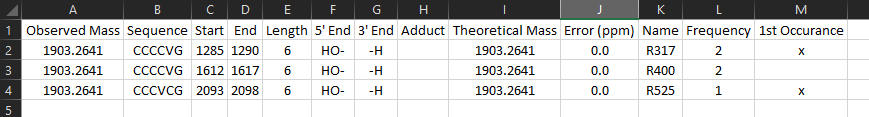


### The Matched Input List worksheet provides a simplified list of matches:
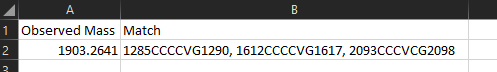


### The Sequence Coverage worksheet shows the location(s) of the matches in the construct sequence:


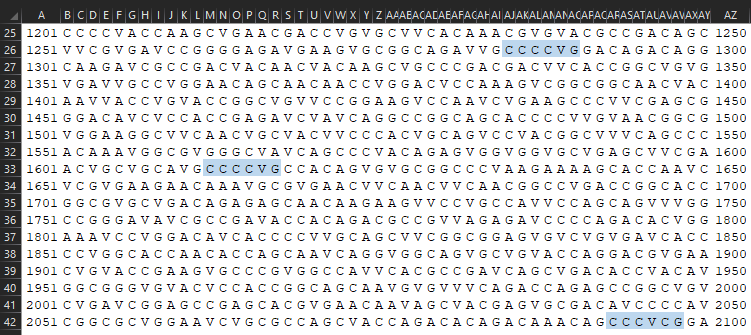


### Oligonucleotide MS Peak ID Given Sequence v9.xlsm can analyze multiple observed masses at once. Matches are putative. If more than one possibility is listed, MS/MS is required for sequence confirmation. Note that it is sometimes the case that the observed LC feature is a mixture of isobaric sequence isomers.

### For MS/MS sequence confirmation, open the Oligonucleotide MS2 Spectrum Matcher v11.xlsm spreadsheet. On the Input Oligonucleotide worksheet, in columns X-AF the elemental composition of bases, ribose, backbone, and oligonucleotide 5′ end and 3′ end can be modified as desired. The following screenshot captures the appropriate settings for N1-methylpseudouridine RNA. The appropriate composition for uracil is C_4_H_3_N_2_O_2_. One hydrogen is absent to account for the bond between the base and ribose.


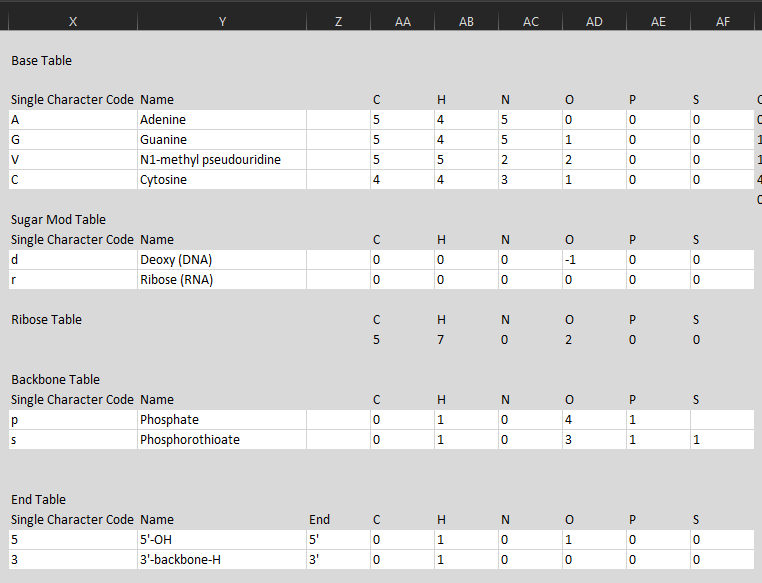


### On the Input Oligonucleotide worksheet, in column AG, the allowed fragmentation ions may be toggled. Usually all are allowed. Fragmentation naming and accounting of McLucky et al (McLuckey, S.A., Van Berkel, G.J. & Glish, G.L. Tandem Mass Spectrometry of Small, Multiply Charged Oligonucleotides. *Journal of the American Society for Mass Spectrometry* 3, 60-70 (1992)):


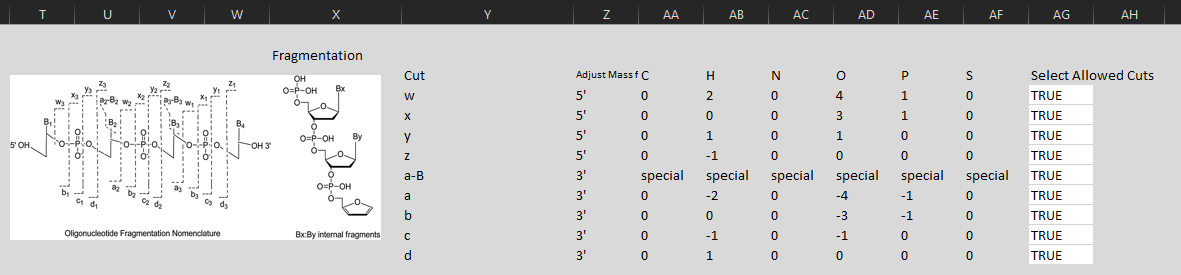


### On the Input Oligonucleotide worksheet enter in the oligonucleotide hypothesis. The *1-Letter Sequence* is entered in cell A5. Modifications to one or more nucleotides can be entered at each nucleotide sequence site in column D. For sequence confirmation of unmodified oligonucleotides column D will be empty. Enter in the *Precursor Charge State* in cell A13. Set the *Peak Annotation Threshold (Minimum % Base Peak*) as desired. A higher percent will de-clutter the Spectrum Match Plot. This has no effect on tabulation of matched ions. Set the *Match Tolerance (ppm)* as desired. For Orbitrap-scanned fragments, a 20 ppm or lower tolerance is appropriate. Toggling *Ignore internal fragment matches in plot* parameter to TRUE simplifies the Spectrum Match Plot by removing the overlay of theoretical matches to fragment ions originating from more than one fragmentation event. Such ions are less informative when inferring sequence confirmation. Exemplary settings:


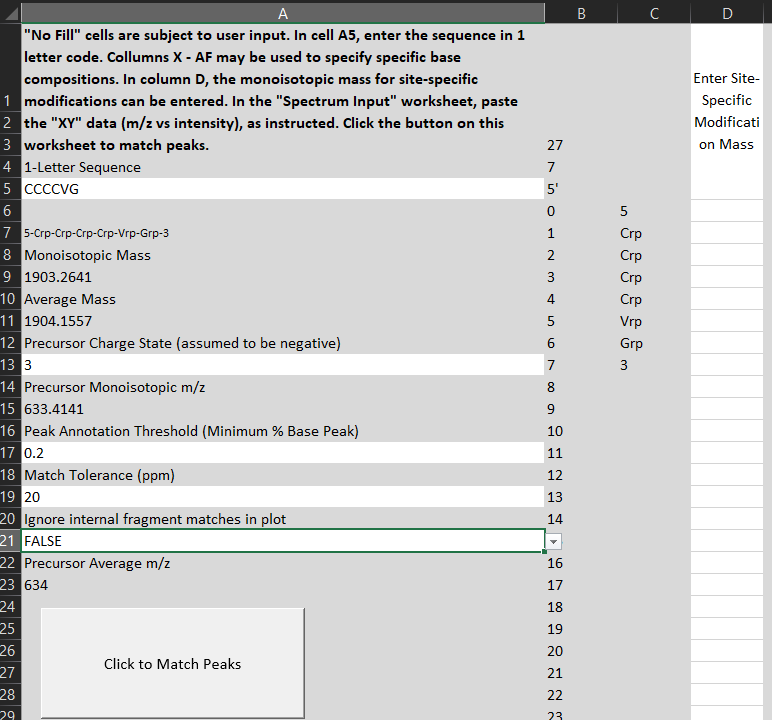


### On the Spectrum Input worksheet, highlight columns A & B and delete their contents using the Delete key.

### Find a representative MS/MS coinciding with the elution of the precursor ion at the unidentified LC/UV feature. In the figure below, the 3^rd^ chromatogram is of MS/MS ion current for precursors with a m/z of 633.4. The 34.37 min spectrum is appropriate for association with the late shoulder feature marked in the top UV chromatogram:


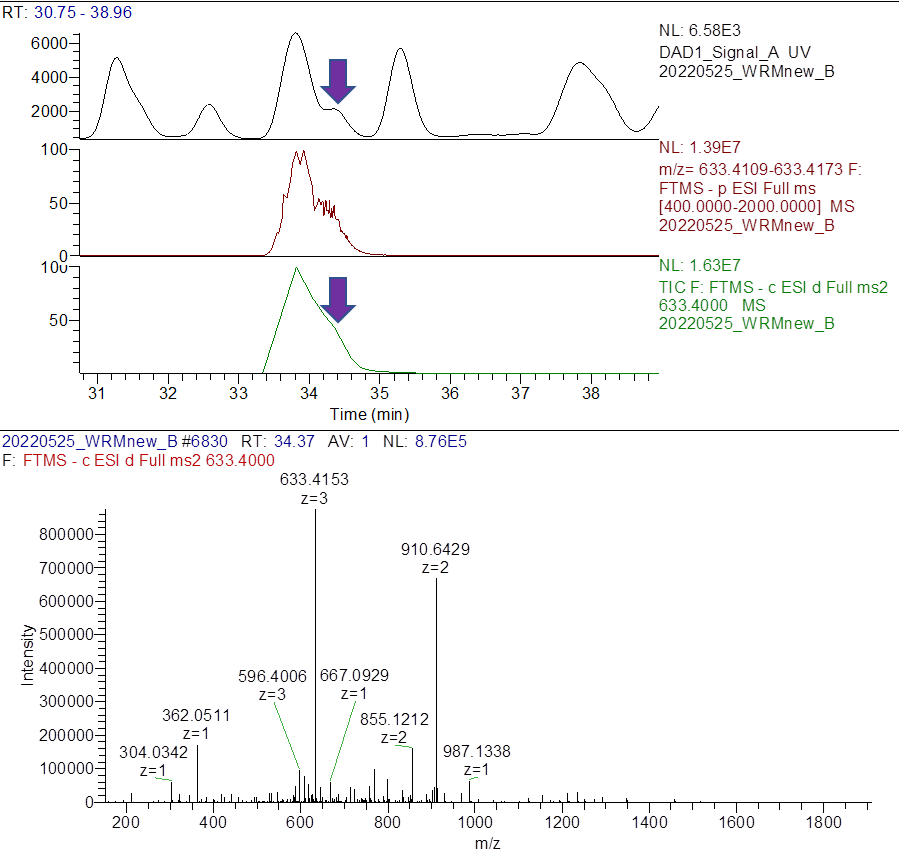


### Right click on the MS/MS and Export to the Clipboard (Exact Mass). Paste the spectrum in cell A1 of the Spectrum Input worksheet.

### Specify the starting row of the “XY” (Mass vs Intensity) data, usually row 8 (1 scan) or 9 (2 or more scans summed). Specify whether the data is Centroid or Profile:


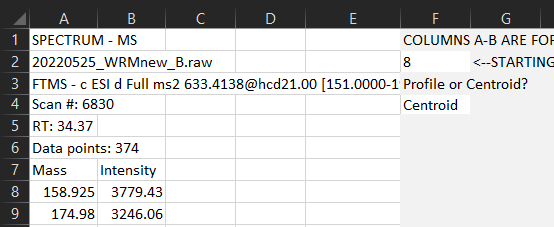


### On the Input Oligonucleotide worksheet, click the Click to Match Peaks macro button. Several outputs are generated. The Theoretical Masses presents de-charged fragment masses in a matrix format. These are compiled in a single list in the Theoretical Masses 1 Colum worksheet. The Type of ion are “Internal” (originating from more than one fragmentation), “5′ ladder” (originating from one phosphodiester fragmentation and containing the 5′-end nucleotide), and “3′ ladder” (originating from one fragmentation and containing the 3′-end nucleotide). The Observed Masses worksheet tabulates each ion, its de-charged mass, and the fragment ions matched to it:


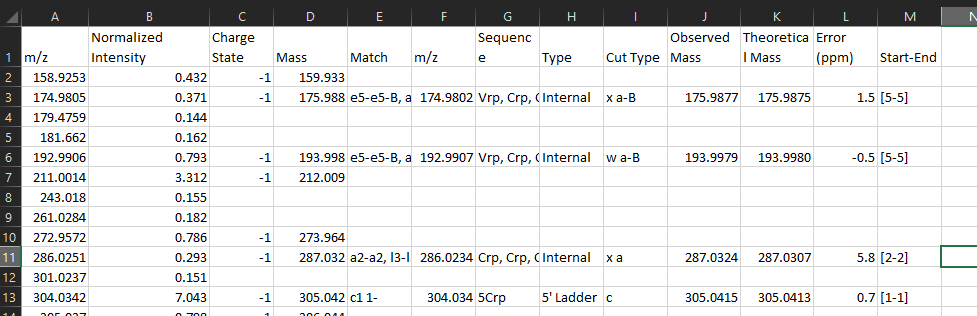


### The Spectrum Match Plot worksheet is the plot of the MS/MS overlayed and annotated with theoretical fragment ion matches. It is an Excel scatter chart that reads columns from the Plot worksheet. As an Excel scatter chart, the plot can be zoomed to specific regions by adjusting the Relative Intensity and m/z axis bounds in Excel (right click on the axis and Format Axis):


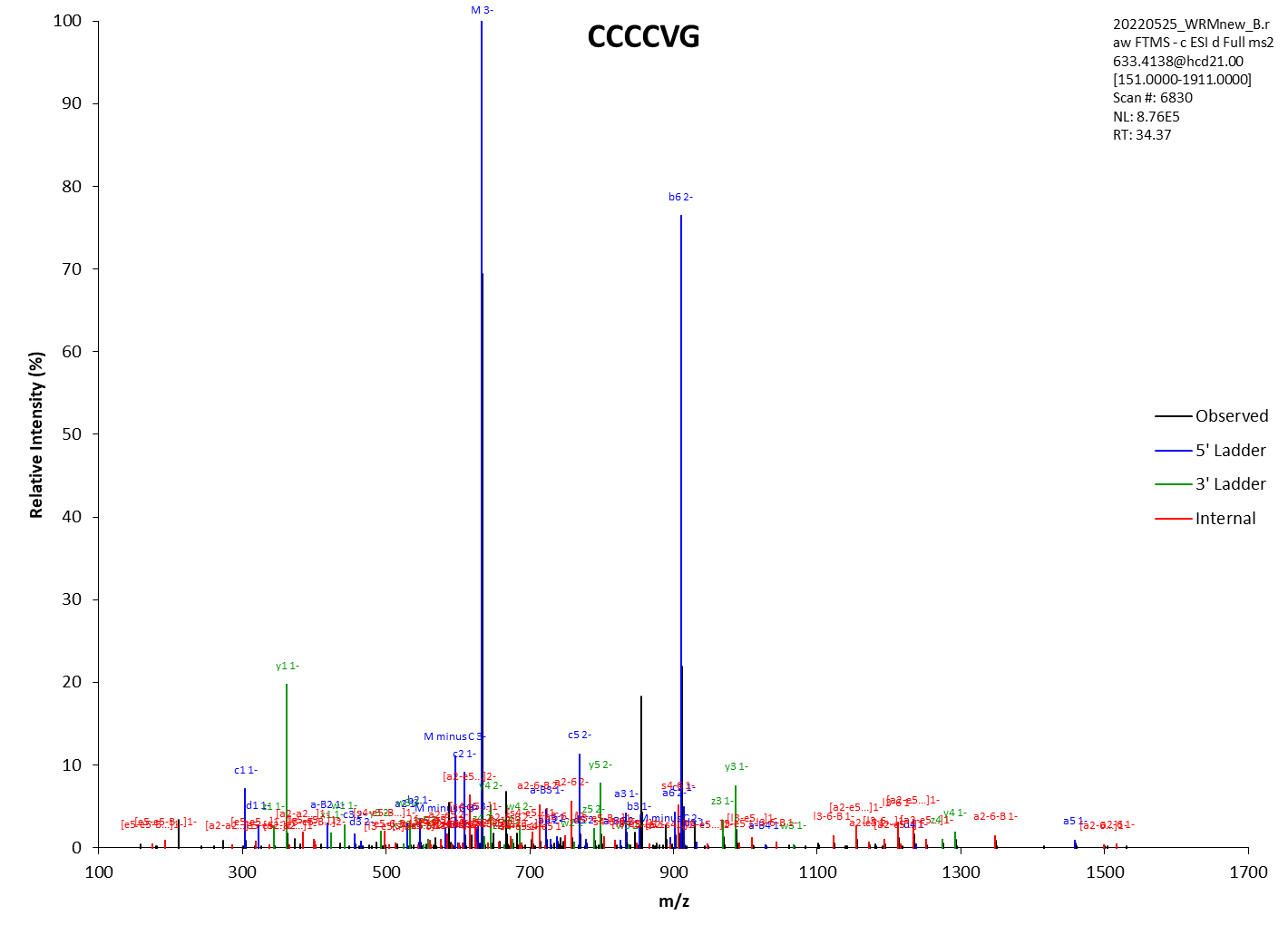


### The Matched Theory worksheet provides a heat-map-colored table of observed ions matched to 5′ and 3′ ladder ions. The heat is based on the ion intensity or sum of intensities for more than one charge state, so that the precursor ion (categorized as the 5′-d ion containing all nucleotides) is often > 100% the base peak and thus is not colored. The ionization conditions in this protocol will often leave substantial ions in the precursor ion channel to minizize internal fragmentation and maximize ladder ion coverage. Here is the CCCCVG match:


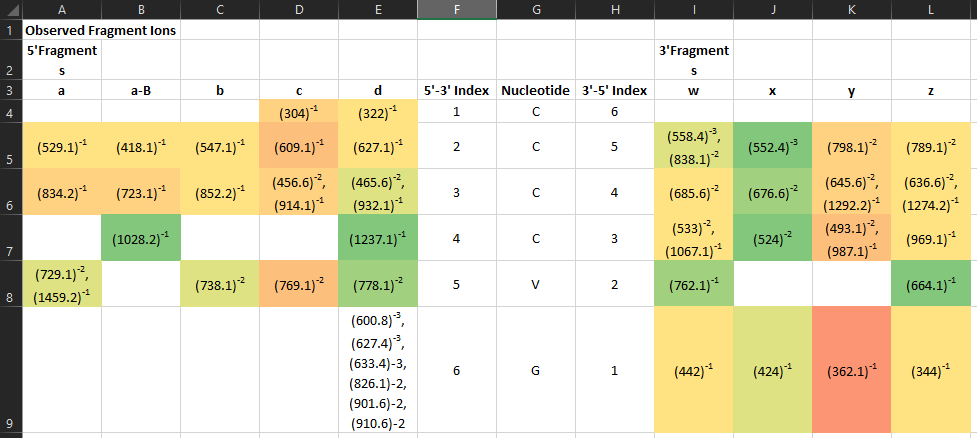


### Consider all likely hypotheses. In this instance, there are two: CCCCVG and CCCVCG, per section 7.7.8. Here is the Oligonucleotide MS Peak ID Given Sequence v9.xlsm Matches worksheet table:


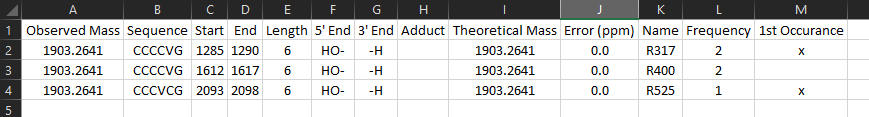
Note that R317 is already annotated in the UV spectrum:


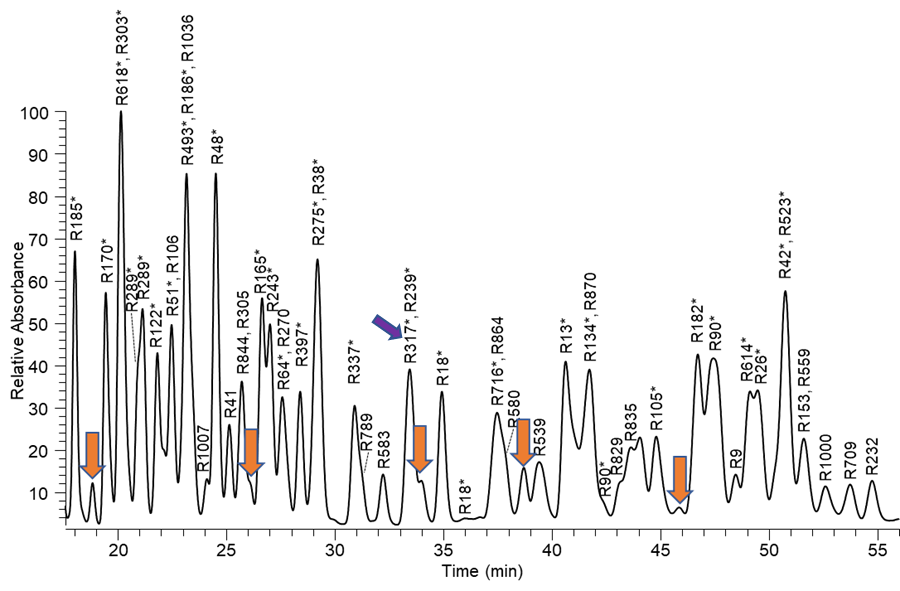


Note that CCCCVG originates from 2 loci in the construct, while CCCVCG originates from 1. Note that the extracted ion intensity of the preceding peak is twice as high as the later peak shoulder:


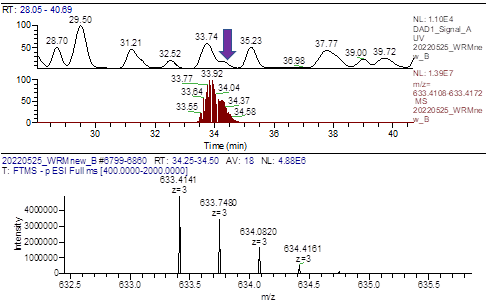


It follows that CCCVCG is the more likely ID. Re-run Oligonucleotide MS2 Spectrum Matcher v11.xlsm changing only the hypothesis: CCCCVG 🡪 CCCVCG.

### Compare the overlaid MS/MS to the spectrum above. Compare the Matched Theory table to the table above:


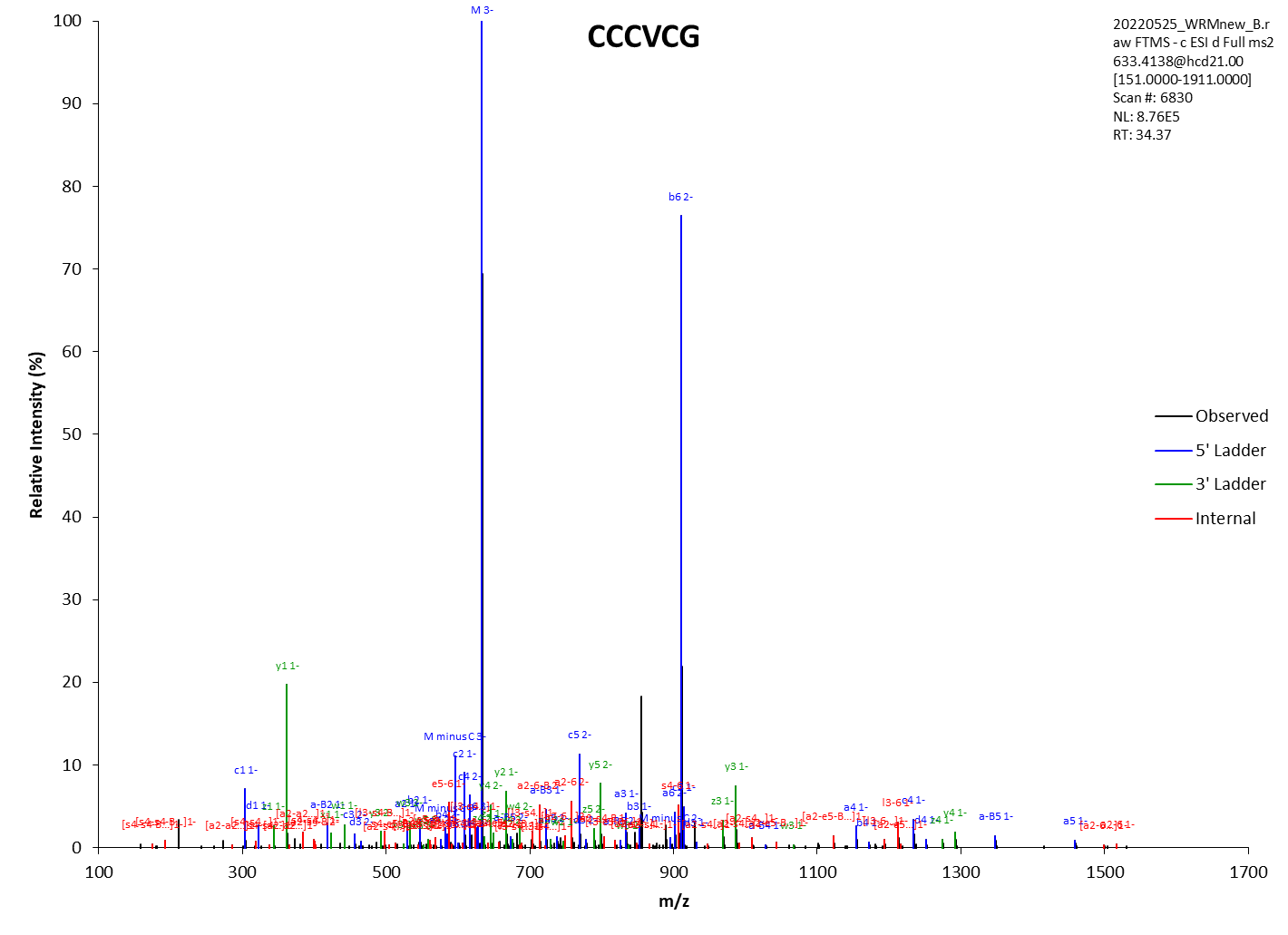

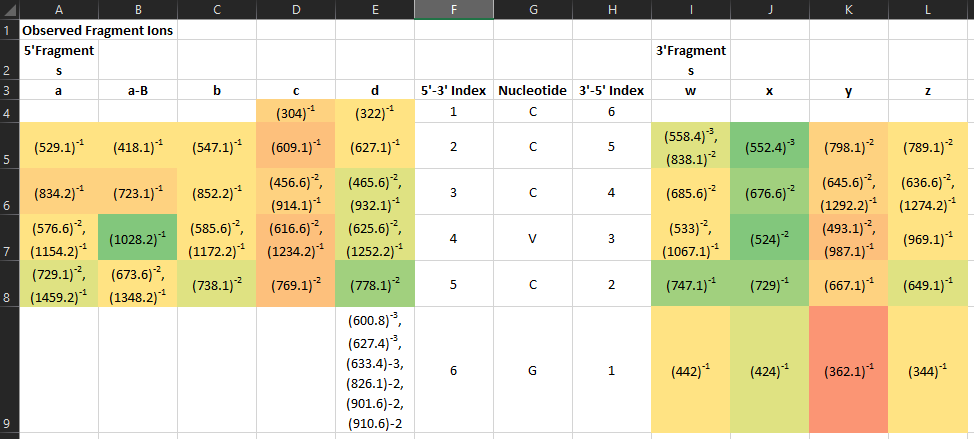
. Note that the CCCVCG offers more conspicuous ladder fragments in the MS/MS overlay. Note that ions of the 5′ ladder fragment a4 are observed in the CCCVCG check but not the CCCCVG check. These all confirm with certainty that the LC/UV unknown feature is the oligonucleotide CCCVCG, with the digest name R525. In short, this is a good match: almost all conspicuous peaks are identified and their identifies are as ladder fragment ions (the 855 ion is the -2 charge state precursor minus cytosine and minus phosphate)

### Update the LC/UV annotated chromatogram in PowerPoint with a text box marking the identified feature.

### Update the Master List spreadsheet with the observation. This is the relevant table section prior to updating:


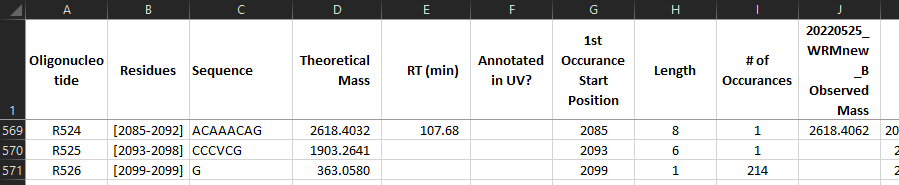
 Update the RT(min) and Observed Mass with the “manual” observations:


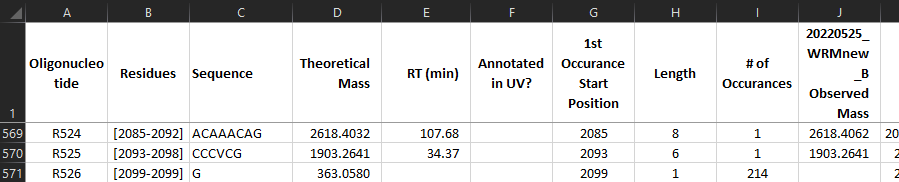


The sequence coverage will be updated automatically. The coverage before updating:
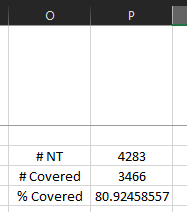
. The coverage after updating:
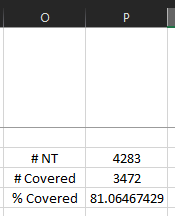


### Proceed with identifying other un-annotated LC/UV features. Some oligonucleotides may contain one or mored missed cleavages (they have internal G residues); in this case the *# Missed Cleavage Sites* in Oligonucleotide MS Peak ID Given Sequence v9.xlsm should be increased to consider this possibility. Some un-identified LC/UV features may not be RNase T_1_ digestion products, so that the methodology in this section fails; the next most likely candidates to try are partial digestion “clipped species”. A strategy to test for clipped species is outlined in section 7.9.

## Step 4: Look for missed oligonucleotides

### The Master List tabulates oberved oligonucleotides in the context of a theoretical list of all possible RNase T_1_ digest product oligonucleotides of the given construct. Rows in which a RT(min) and Observed Mass are not listed indicate expected RNase T_1_ digest products that were not identified by the automated BioPharma Finder software. This can occur when the un-identified product oligonucleotide coelutes or partially coelutes with sequence isomer(s). In addition, poly(A)-tail containing oligonucleotides may not be identified by the BioPharma Finder method presented here, though the Xtract deconvolution algorithm in the Intact Mass module of BioPharma Finder or FreeStyle works well for MS confirmation of poly(A) tail-containing oligonucleotides (such olignucleotides do not require MS/MS for sequence confirmation). There may be other reasons for non-identification, though the chromatography and digestion conditions presented in this method are optimized for the detection of every possible oligonucleotide—if after searching the oligonucleotide is not found, the construct sequence may be at fault!

### For the purpose of heightened characterization, a sequence coverage of 97.0% is considered adequate. This means that some expected oligonucleotides, as yet un-identified, may excape tabulation. It is incorrect to interpret an un-identified olignucleotide in the Master List as not observed. The absence of the oligonucleotide’s observed mass listing simply means its inclusion was not needed to achieve 97.0% sequence coverage.

### Select a candidate un-identified oligonucleotide. To meet the coverage criterion as quickly as possible, start with oligonucleotides with many repeat occurances. Use Excel filtering. The most frequent is G:


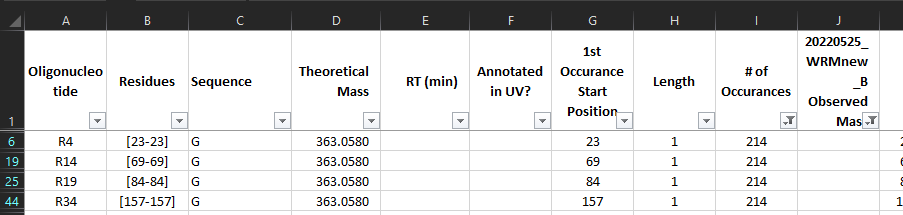


This nucleotide is the first conspicuous peak in the UV chromatogram. This can be observed between 2 and 3 min (R4*):


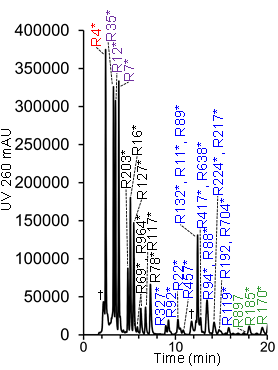


The divert valve setting listed in Table 8 (section 6.3.10) means a normal acquisition misses the MS data for this feature. A single injection of sample can be made with a divert valve at 2 min to confirm the presence of G. This injection serves as a reference for all future oligonucleotide mapping acquisitions on the system, in which the divert valve is set to 3 min (or even later), to protect the spectrometer from deleterious ions. If the LC/UV chromatogram of the current data matches the early LC/UV chromatogram of historical data in which the G nucleotide was observed by MS, sequence coverage should be augmented with the G digestion product observation. Update the table with the RT (min) and a character in the Observed Mass that can footnote the historical reference:


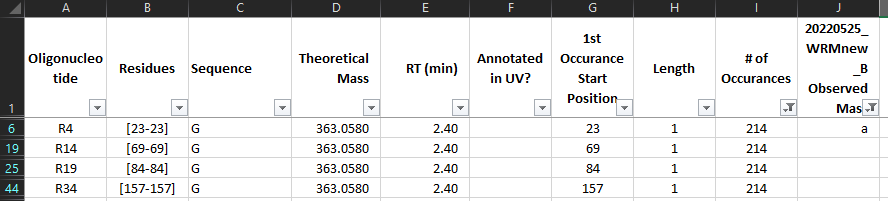


Autofill the RT (min) and Observed Mass entries to fill every “G” occurance: select the small bottom right square of the selected cell
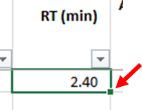
and drag to the bottom of the filtered table (do not double-click because the table is filtered):


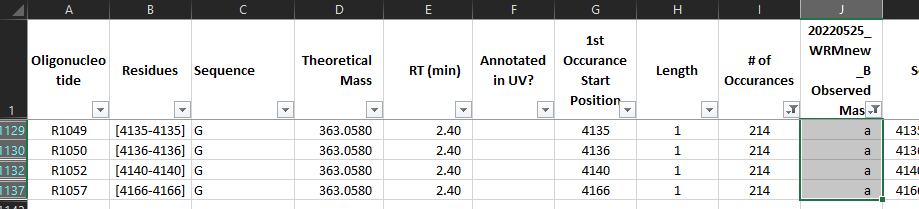


Unfilter the table and confirm that the sequence coverage is updated: .

### Select the next candidate using Excel filtering of the Master List. Two-mers are often missed by the automated software, but their elution and spectrometry are straightforward. Be sure to fill in all rows marking different loci of the same oligonucleotide. Focus on long-sequence candidates once short oligonucleotides originating from many loci have been examined. A long-sequence example is presented to illustrate how to identify any missing expected digestion product oligonucleotide:


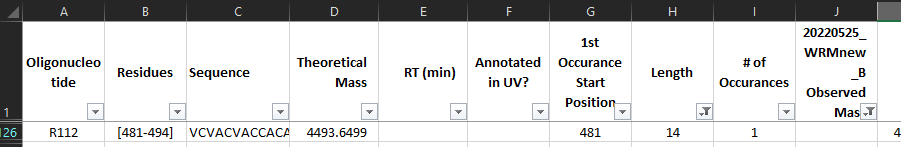


### Extract the ion current for the candidate using one or several likely charge state ions. The Oligonucleotide MS2 Spectrum Matcher v11.xlsm Input Oligonucleotide worksheet provides a m/z calculator for a given sequence. Enter the *1-Letter Sequence* and a *Precursor Charge State (assumed to be negative)* that gives a value near 700 m/z:


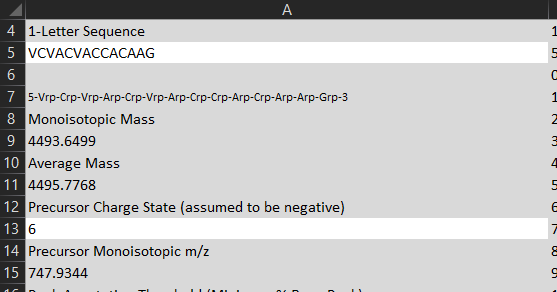


In Qual Browser, extract the ion current. Sum across the peak to confirm the monoisotopic mass matches the theoretical value:


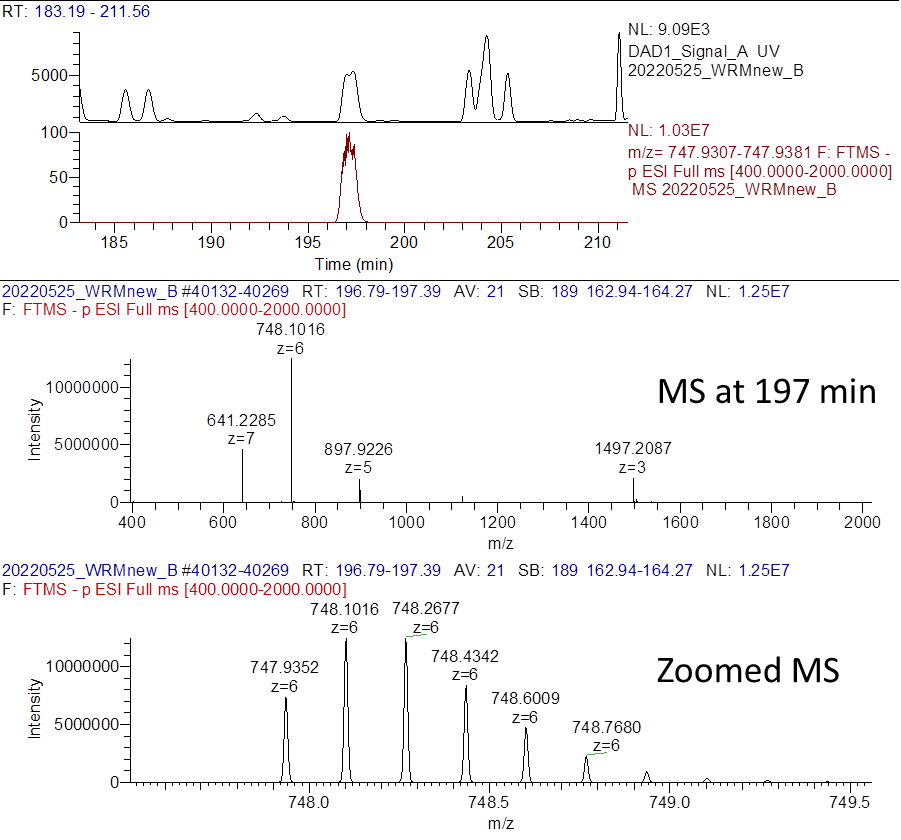


### Proceed as direct in sections 7.7.13 - 7.7.26: Find an MS/MS, consider all likely sequences, confirm the top match is a good match, update the LC/UV chromatogram (if space allows), and update the Master List table. Here is the MS/MS match for a 14-mer.


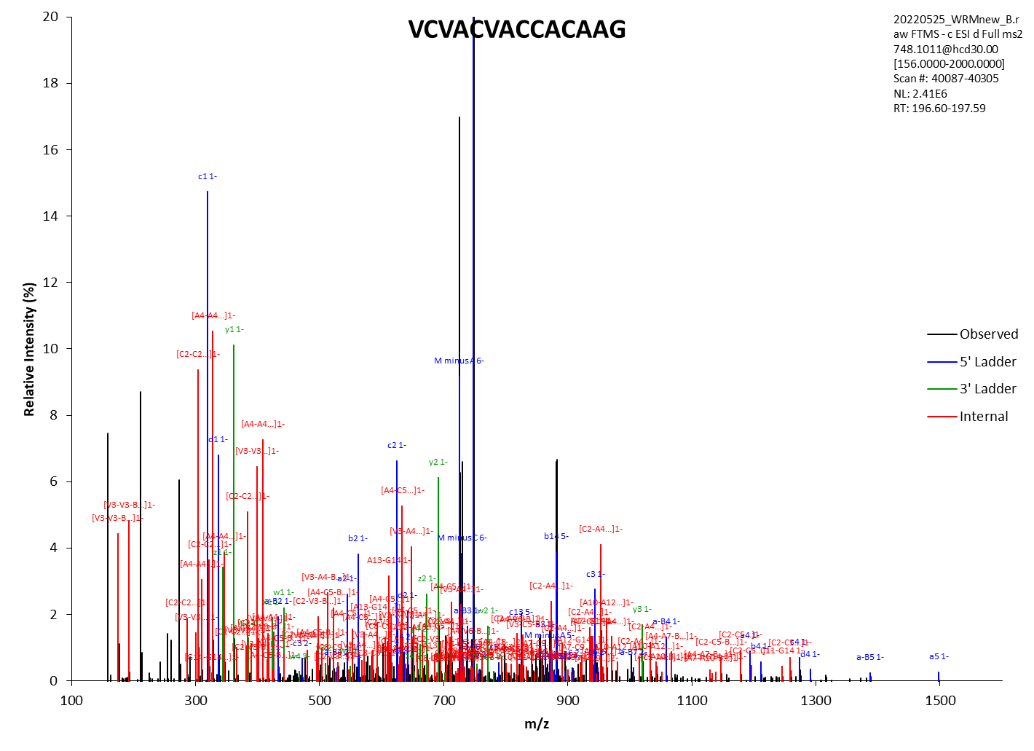


As expected, with a higher charge state and longer oligonucleotide, more internal fragmentation is observed than with the smaller oligonucleotide example above. Toggling the *Ignore internal fragment matches* in plot to TRUE gives a cleaner ladder fragment overlay:
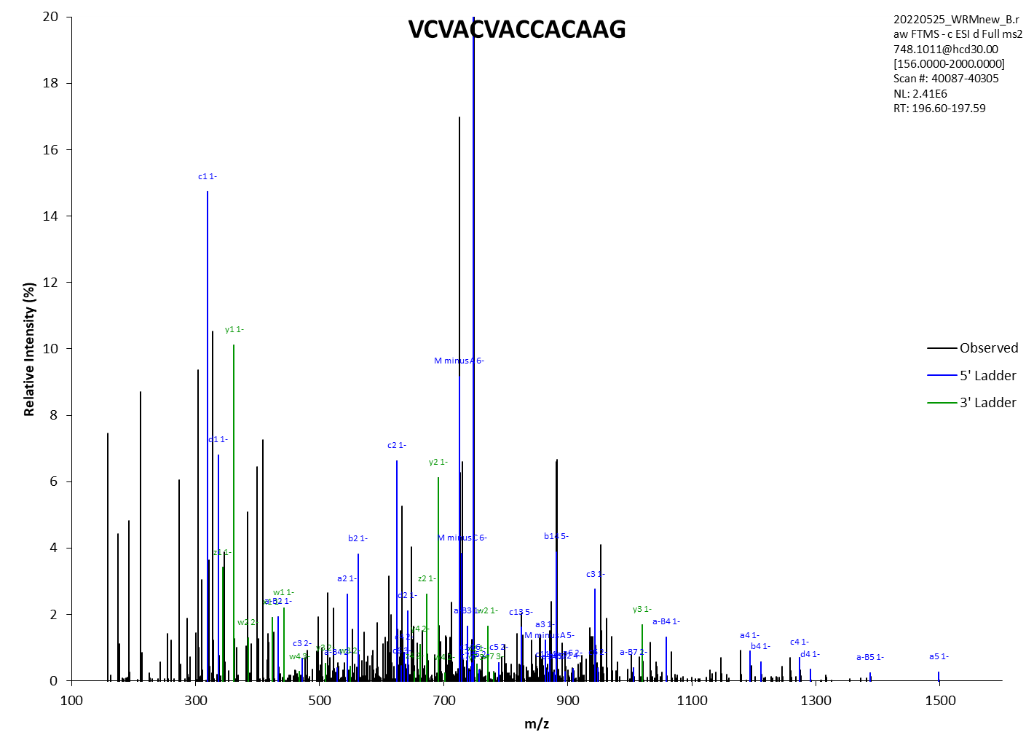


This is a good MS/MS match because the full sequence can be inferred from ladder ions, and because most observed ions are explained (though many are internal fragments).

## Step 5: Identify clipped species

### The following example illustrates a clipped species. The LC/UV, LC/extracted ion current MS, and MS data for the unknown LC/UV feature at 192.3 min:


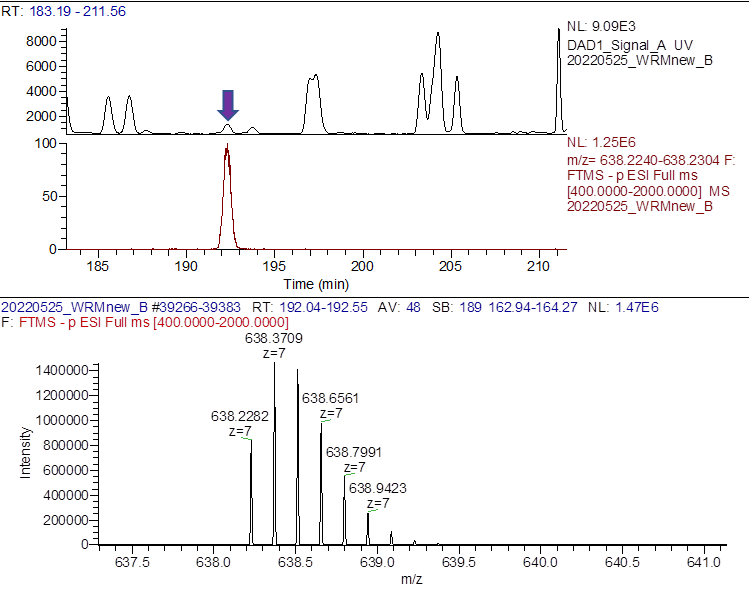


A search using the Oligonucleotide MS Peak ID Given Sequence v9.xlsm spreadsheet with *# Missed Cleavage Site* set to 10 did not yield any results for the de-charged 4474.6483 mass:
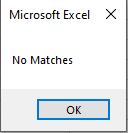


### Open the Oligonucleotide Composition from Mass Calculator v2.xlsm spreadsheet. In the Input worksheet, enter the de-charged mass in cell A2. Set the *ppm threshold* to 5 ppm and *Monoisotopic or Average Mass?* to Monoisotopic. In columns I-L, define the nucleotide residue masses as was done in the Oligonucleotide MS Peak ID Given Sequence v9.xlsm spreadsheet. In columns O-Q, enter up to 3 possible modifications to consider, such as 3′-end ribose cyclic phosphate (cp) or loss or gain of phosphate:


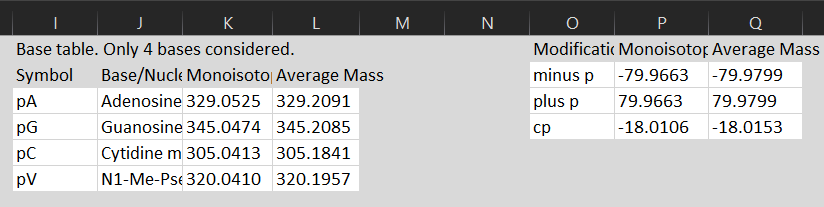


### Click the Execute the FindCompositions Macro button. The Match worksheet lists the possible combinations of nucleotides:


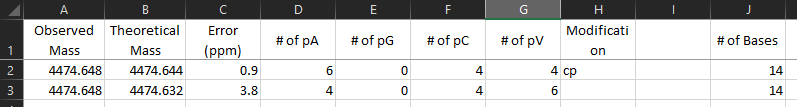


### Use the criteria in 7.9.3 as filters in the 5′-side Clip List and/or 3′-side Clip List worksheets of Oligonucleotide MS Peak ID Given Sequence v9.xlsm spreadsheet.

#### 5′-side Clips begin after an expected RNase T_1_ G cut, but do not end at a G and often have a cyclic phosphate bridging their 3′-end ribose’s 2′ and 3′ carbons (a “cp” modification).

#### 3′-side Clips end at expected RNase T_1_ G cut, but do not begin after a G. Usually their 5′-end ribose 5′ carbon is hydroxylated, just as with an RNase T_1_ product (it is not phosphorylated).

#### Set the # of Bases in Cell O2 of the 5′-side Clips or 3′-side Clips worksheet equal to the value in the Oligonucleotide Composition from Mass Calculator v2.xlsm spreadsheet (14 in Cell O2 of the 5′-side Clips worksheet in this example). The result:


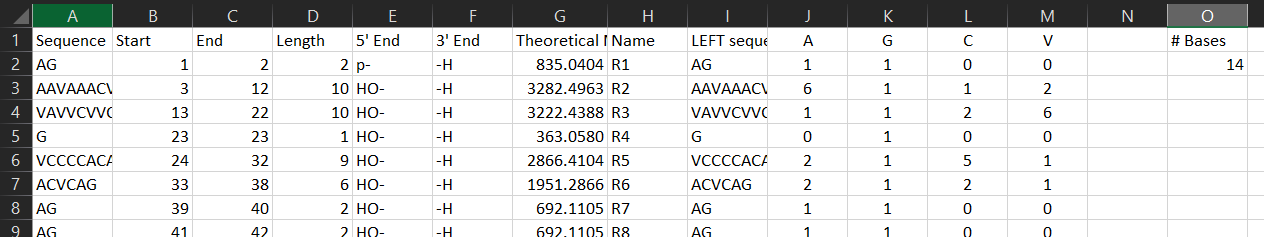


#### Because the # of pG is zero in the two possibilities listed (see the figure in Click the Execute the FindCompositions Macro button. The Match worksheet lists the possible combinations of nucleotides:7.9.3), the hypothesis is that the unknown species is an oligonucleotide originating from an RNase T_1_ digestion cut on its 5′-end and a non-specific degradation cut or incomplete transcription termination on its 3′-end. Historical data has shown that in this case, a cyclic phosphate connecting the 3′ and 2′ carbons on the 3′ ribose is the more likely species. Filter to find candidates that have 6 pA nucleotides, 4 pC nucleotides, and 4 pV nucleotides (V in this example, as above, is for N1-methylpseudouridine). There is only one possibility:


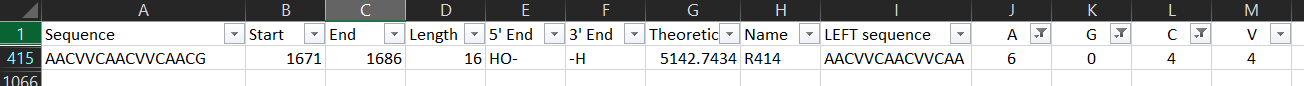


### Check the mass of the hypothesis using the Oligonucleotide MS2 Spectrum Matcher v11.xlsm Input Oligonucleotide worksheet. Enter in the truncated sequence and update the modification column for cyclic phosphate (in this example). Cyclic phosphate is accounted for by a loss of water on the last nucleotide:


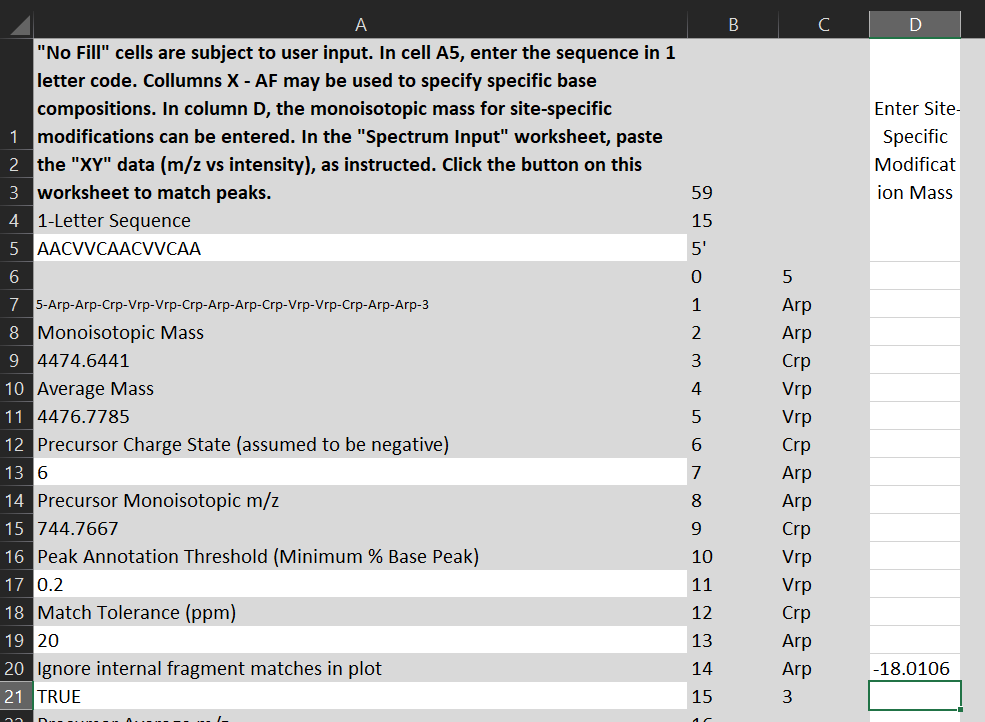


In this example the 4474.6483 observed mass agrees well with the theoretical *Monoisotopic Mass* (cell A9), which the spreadsheet calculates based on the sequence and accounts for the cyclic phosphate modification: (4474.6483 ̶ 4474.6441) ÷ 4474.6441 × 1000000 = 0.94 ppm.

### Check the MS/MS match. As before, find an MS/MS at the apex of the peak, paste into the Spectrum Input worksheet, and click the Click to Match Peaks button on the Input Oligonucleotide worksheet. Sometimes for larger oligonucleotides a lower charge state provides a simpler spectrum; here is the matched MS/MS for the -3 charge state clipped species (1490.5 m/z) with internal fragments toggled off:


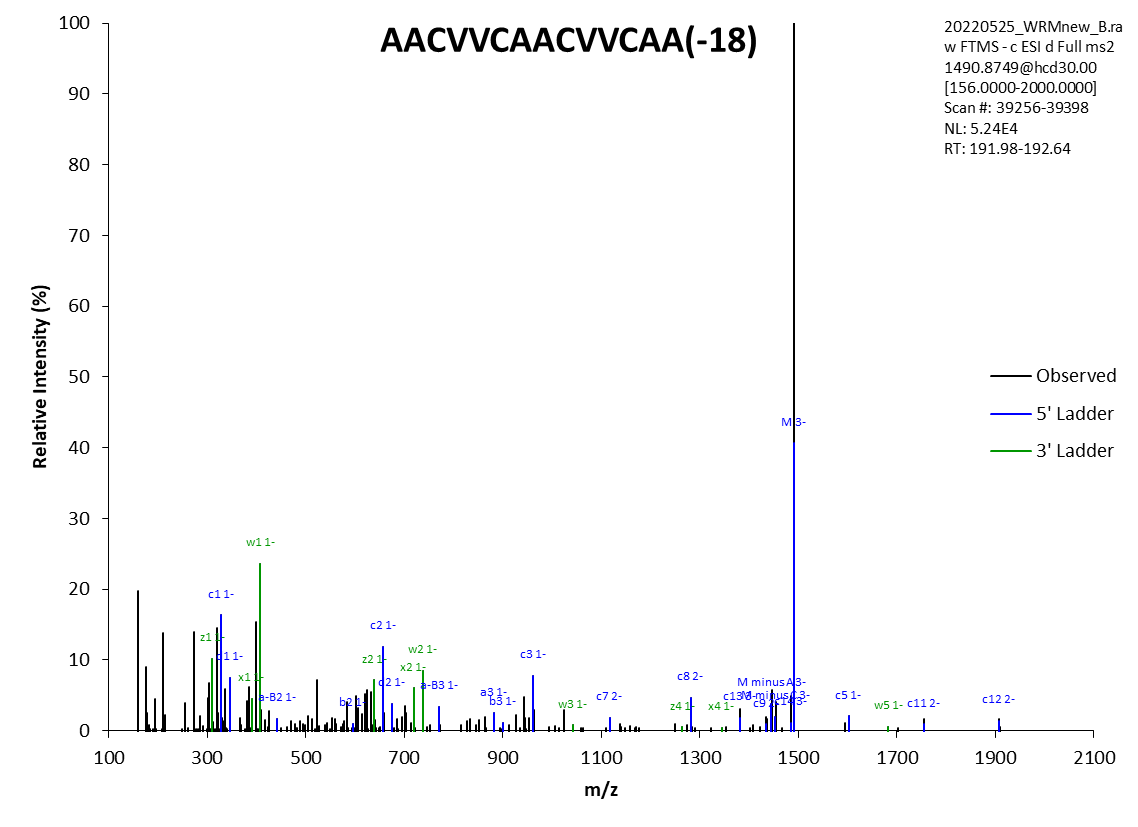


Taken together, the MS and MS/MS provide definitive identification of this feature.

### Update the LC/UV chromatogram and Master List with the identification. The convention for clipped oligonucleotides is to list the RNase T_1_ oligonucleotide from which the originate and specify the start and ending nucleotide residue #s in paratheses. The cyclic peptide ID is signified by a “cp” suffix. Refer to the filtered theoretical table above, here repeated:


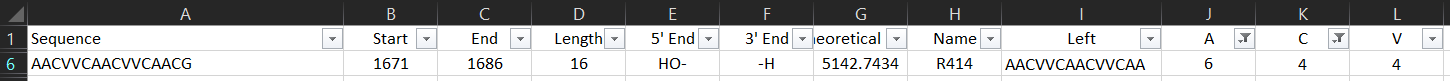


Thus the ID is R414 (1671-1684) cp. Note that it is not R414 (1671-1686) cp.

### Update the Master List. Insert a row under or above the listed RNase T_1_ digestion product (R414 in this case). If it is a 5′ clip species, insert the row above the R414 entry row. If it is a 3′ clip species, insert the row below the R414 entry row. Fill in the Oligonucleotide, Residues, Sequence, Theoretical Mass, RT(min), and Observed Mass data:


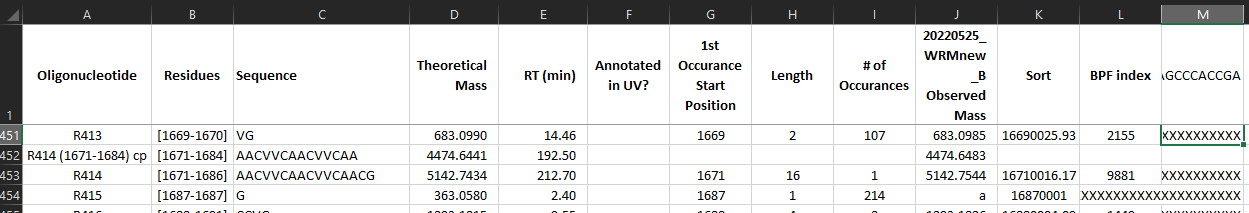


Autofill columns G-I starting above the inserted row through the new row:


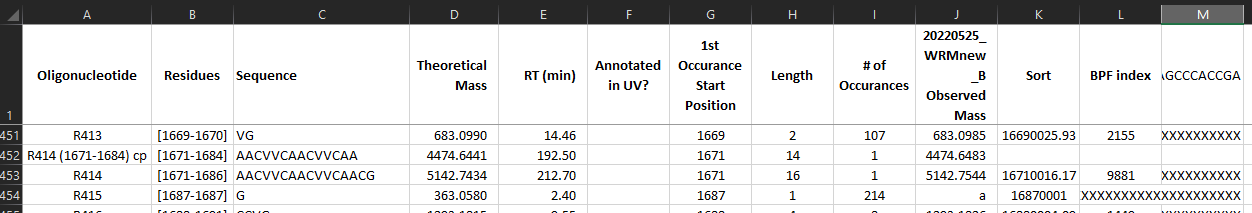


Autofill the last contiguous column on the right (column M for one sample) through the new row:


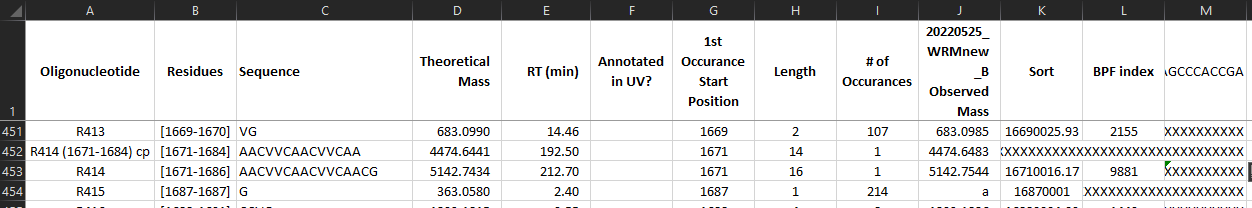


These autofilled formulae allows for the proper sequence coverage calculation. The Sort and BPF index meta data are not needed (though sorting the table on the Sort field works best with an entry present—simply subtract or add 0.001 to the R414 Sort value).

# Calculations and Formulae

## Calculation of % sequence coverage:

$\% Sequence Coverage= 100 x \left( \frac{\# mRNA bases detected}{\#mRNA bases} \right)$

## It is recommended to utilize Biopharma Finder’s calculation of mass accuracy when available, but mass accuracy may be manually calculated and reported in parts per million (ppm) by the following formula:

$ppm error =\left\lceil\frac{Observed mass-Theoretical mass}{Theoretical mass}\times1,000,000 \right\rceil$

# System Suitability CRITERIA

## The mass spectrometer calibration check passed.

## The total ion chromatogram is smooth.

## **Mass accuracies for species present at >5% base peak chromatogram height are within 5 ppm.**

## The chromatographic profile of the system suitability control (reference material) at the beginning of the sequence should be grossly comparable to historical system suitability data; that is, they resemble each other in most respect.

### **When a new mRNA molecule is mapped, the analyst should establish suitability with an already-estabilished mRNA molecule. The new map can then serve as the chromatographic benchmark for future analyses of the same mRNA molecule in suitability assessment.**

# Assay Acceptance CRITERIA

## All applicable system suitability criteria should be met.

## Biopharma Finder successfully assigns unique oligonucleotides as belonging to the target theoretical mRNA in the decoy sequence search (see Appendix Attachment F).

### Repeat the instructions of Step 1: Automated Data Search (section 7.3), using the target sequence and at least three other decoy sequences. This can be enabled in BioPharma Finder version 5.1. Only one .raw needs to be searched. If using an earlier version consult with the assay SMEs for a tool to create a .fasta file with the target sequence and decoy sequences. Each decoy sequence is a random arrangement of nucleotides having the same composition of nucleotides as the target sequence.

### The mRNA sequences from different mRNA molecules are expected to contain significant overlap in the masses of oligonucleotides generated by an RNAse T1 digest, because only four nucleotides are available to create unique sequence structures. The decoy sequence search simultaneously tests the quality of MS/MS fragmentation and ability for Biopharma Finder’s MS/MS sequencing module to successfully call a correct identification based on the MS/MS spectra, even amongst the presence of sequence isomer decoys with the exact same theoretical monoisotopic mass and highly similar sequence and fragmentation spectra as the true oligonucleotide.

### Successful identification of the true oligonucleotides in the decoy search provides validation of the overall sequence identification as true (Attachment D provides an example). It also validates Biopharma Finder’s ability to differentiate sequence isomers from each other using MS/MS, when they occur within the true oligonucleotide sequence.

# Reporting OF RESULTS

## Oligonucleotide Mapping Readout (Section 7.2) describes the heightened characterization reporting guidelines.

## Data, calculations, and laboratory records are to be collected and stored in laboratory notebooks or according to laboratory guidelines.

## Visually assess comparability of chromatograms if performing a comparability analysis of multiple drug substance batches. See Appendix Attachment C for an example of representative mRNA mapping chromatograms that are comparable.

# List of Attachments

## Attachment A: Guidance for Customizing Bases, Linkers, Ribose Sugars, or Modifications for Theoretical Oligonucleotide Sequences within Biopharma Finder 4.1

## Attachment B1-6: Representative RNase T1 Oligonucleotide Mapping Profiles of 3 Batches of BNT162b2 modRNA DS, also demonstrating batch comparability.

## Attachment C: Example Excerpt of Mass Table Suitable for Oligonucleotide Mapping Report

## Attachment D: Exemplary Biopharma Finder Decoy Search Results

## Attachment E: Setting up mRNA sequence in Biopharma Finder

## Attachment F: ProteinMetrics Byos deconvolution settings for the analysis of Poly(A)-tail spectra

**-END OF PROCEDURE-**

# Appendix

**Attachment A**

## Guidance for Customizing Bases, Linkers, Ribose Sugars, or Modifications for Theoretical Oligonucleotide Sequences within Biopharma Finder 5.1


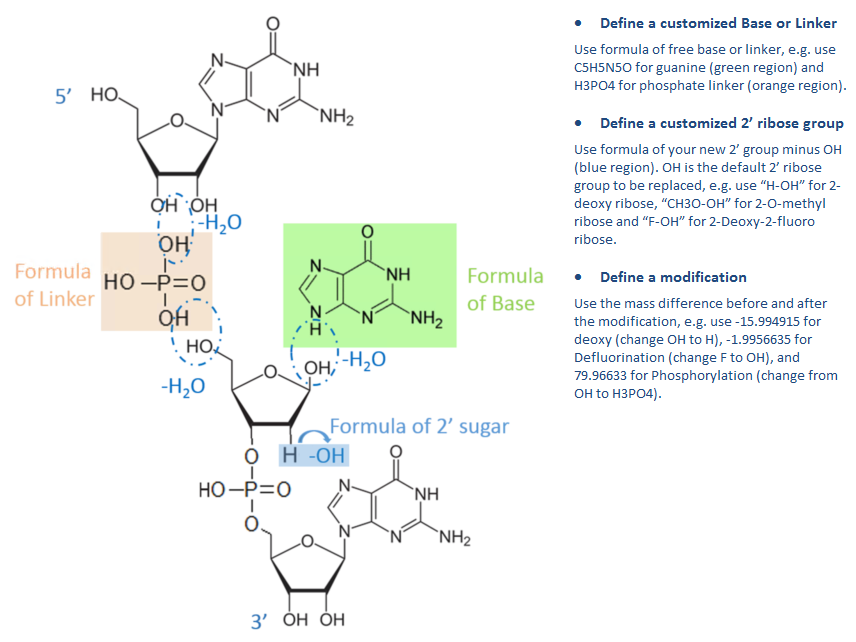


**Attachment B1**

## Representative RNase T_1_ Oligonucleotide Mapping Profiles of 3 Batches of BNT162b2 modRNA DS, 0-18 min

IP RP-UHPLC/UV/MS/MS RNase T_1_ oligonucleotide map of BNT162b2 Original DS in six time segments: 0-18 min, 18-56 min, 56-107 min, 107-157 min, 157-206 min, 207-254 min. “R” represents oligonucleotide RNase T1 digestion products indexed from the 5′ to 3′ end. “*” denotes a sequence-repeat oligonucleotide, where the single peak assignment represents all identical oligonucleotides in the sequence. Oligonucleotides named as R[#-#] are missed cleavage oligonucleotides. The index numbers refer to the 5'-end R[#] and 3'-end R[#] making up the missed-cleavage oligonucleotide. Oligonucleotides named as R[#](#-#) are oligonucleotides in which the 5′-side or 3′-side does not orignate from an RNase T_1_ cleavage. The #s in the parenthesis (#-#) indicate the locus start and end positions in the sequence for these ‘clip’ species. "cp" designates a cyclic phosphate (connecting the 3' and 2' ribose carbons) at the 3'-end ribose of the oligonucleotide.

**Attachment B2**

## Representative RNase T_1_ Oligonucleotide Mapping Profiles of 3 Batches of BNT162b2 modRNA DS, 18-56 min

**Attachment B3**

## Representative RNase T_1_ Oligonucleotide Mapping Profiles of 3 Batches of BNT162b2 modRNA DS, 56-107 min

## Attachment B4 - Representative RNase T_1_ Oligonucleotide Mapping Profiles of 3 Batches of BNT162b2 modRNA DS, 107-157 min

## Attachment B5 - Representative RNase T_1_ Oligonucleotide Mapping Profiles of 3 Batches of BNT162b2 modRNA DS, 157-207 min

## Attachment B6 - Representative RNase T_1_ Oligonucleotide Mapping Profiles of 3 Batches of BNT162b2 modRNA DS, 207-254 min

## Attachment C - (Partial) Table of Observed and Theoretical RNase T_1_ Digest Oligonucleotide Masses

| **Oligonucleotide** | **Residues** | **Sequence** | **Theoretical Mass** | **RT (min)** | **UV Peak Annotation^b^** | **1st Occurance Start Position** | **Length** | **# of Occurances** | **Observed Mass^c^** |
| --- | --- | --- | --- | --- | --- | --- | --- | --- | --- |
| R1^a^ | [1-2] | AG | 1239.1376 | 37.99 | y, manual | 1 | 2 | 1 | 1239.1380 |
| R2 | [3-12] | AAVAAACVAG | 3282.4963 | 154.09 | y | 3 | 10 | 1 | 3282.5022 |
| R3 | [13-22] | VAVVCVVCVG | 3222.4388 | 136.04 | y | 13 | 10 | 1 | 3222.4404 |
| R3-6 | [13-38] | VAVVCVVCVGGVCCCCACAGACVCAG | 8349.1621 | 225.76 |  | 13 | 26 | 1 | 8349.1680 |
| R4 | [23-23] | G | 363.0580 | 2.45 | y, manual | 23 | 1 | 214 | 363.0584 |
| R5 | [24-32] | VCCCCACAG | 2866.4104 | 114.46 | y | 24 | 9 | 1 | 2866.4194 |
| R6 | [33-38] | ACVCAG | 1951.2866 |  |  | 33 | 6 | 1 |  |
| R7 | [39-40] | AG | 692.1105 | 4.15 | y, manual | 39 | 2 | 71 | 692.1123 |
| R8 | [41-42] | AG | 692.1105 | 4.15 | y, manual | 41 | 2 | 71 | 692.1123 |
| R9 | [43-48] | AACCCG | 1936.2869 | 48.71 | y | 43 | 6 | 1 | 1936.2881 |
| R10 | [49-56] | CCACCAVG | 2561.3691 | 88.95 | y | 49 | 8 | 1 | 2561.3704 |
| a. R1 is capped on its 5′-end nucleotide, which adds 547 Da to the given sequence’s mass.  b. “y”: the oligonucleotide was identified by BioPharma Finder software and was annotated on the UV chromatogram; “y, manual”: the oligonucleotide was identifed by the analyst and was annotated on the UV chromatogram.  c. Identified oligonucleotides are signified with an observed mass. Blank entries signify expected RNase T_1_ digest product that were not identified in the data. Not every identified oligonucleotide may be annotated in the UV chromatogram in order to simplify the chromatogram presentation. | | | | | | | | | |

## Attachment D - Exemplary Biopharma Finder Decoy Search Results

##

The top pane in this figure is illustrative but is not a requirment of the suitability assessment. A proper suitability readout is the bottom pane, which here shows that the majority of peaks after 30 min are mapped to the true sequence and not decoy sequences. A few peaks in this region may map to decoy because of the coincidence of identical oligonucleotides; this is expected and acceptable.

## Attachment E1 –Setting up mRNA Sequence in Biopharma Finder
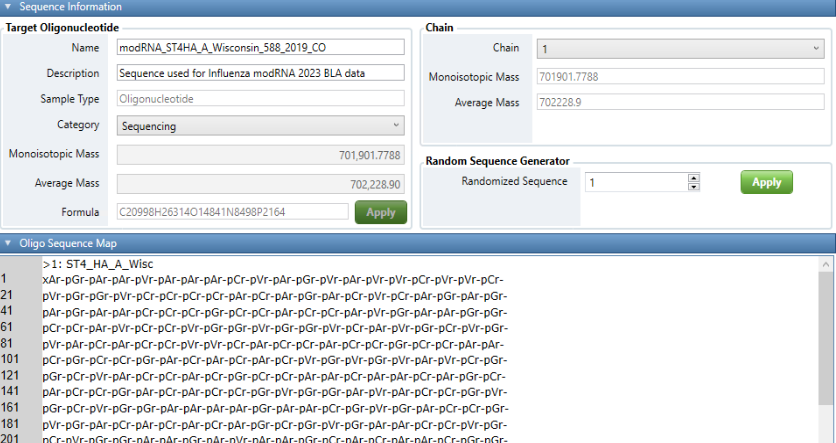


## Attachment E2 - Setting up mRNA Sequence in Biopharma Finder


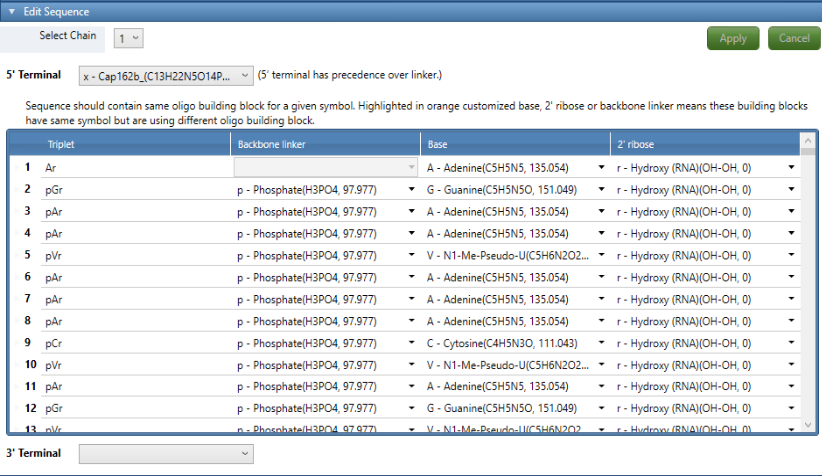


## Attachment E3 - Setting up mRNA Sequence in Biopharma Finder


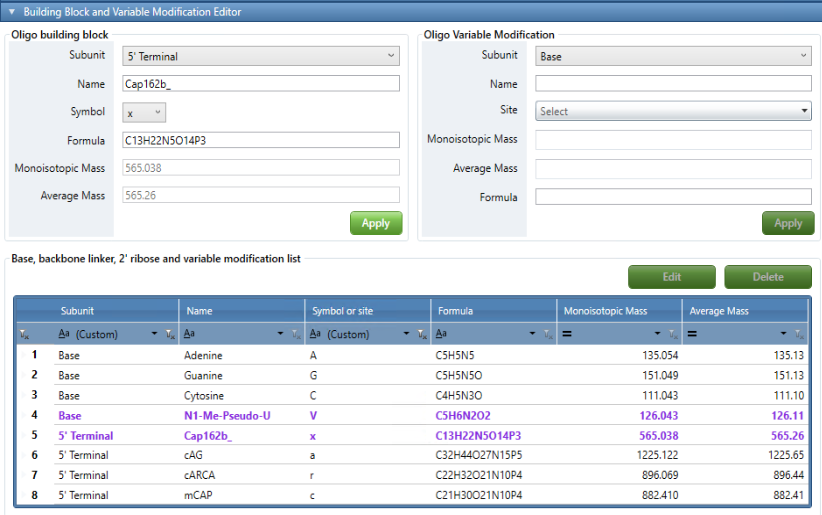


## Attachment E4 - Setting up mRNA Sequence in Biopharma Finder


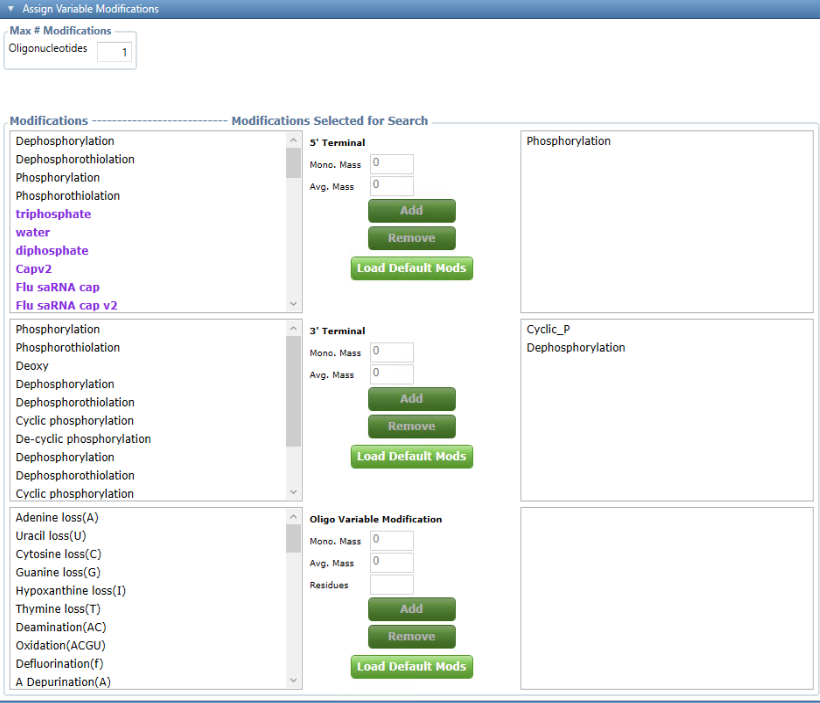


## Attachment F1 – Setting up Byos Oligonucleotide Mapping Module

##
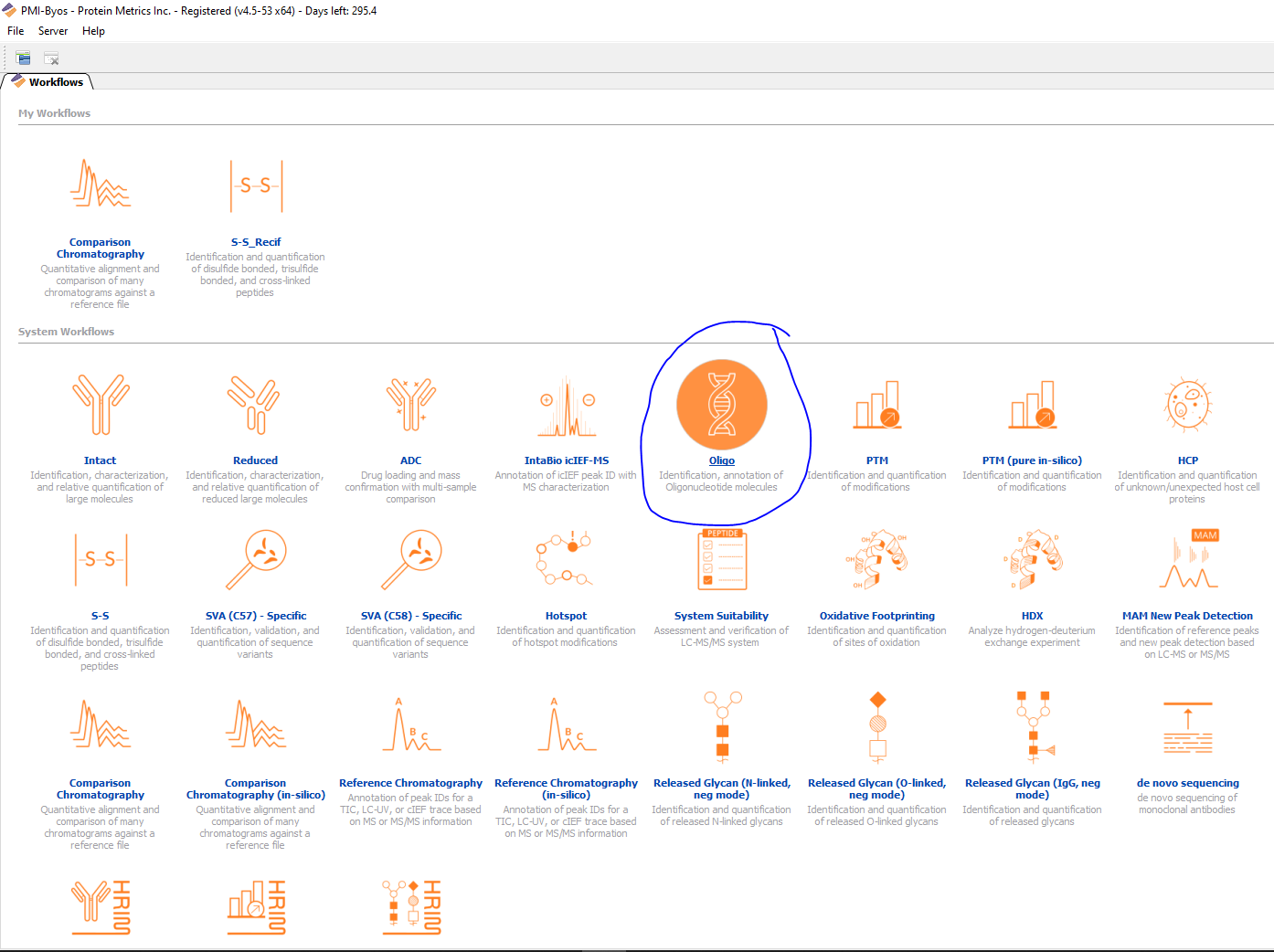


##

## Attachment F2 – Exemplary Parameters for Deconvolution of A30 Region

## Attachment F3 – Exemplary Parameters for Deconvolution of L70 Region
