# Supplementary material for: Oligonucleotide mapping via mass spectrometry to enable comprehensive primary structure characterization of an mRNA vaccine against SARS-CoV-2
Source: Sci Rep. 2023 Jun 3;13:9038. doi: 10.1038/s41598-023-36193-2 (PMC10239040; doi:10.1038/s41598-023-36193-2)

## Slide 1
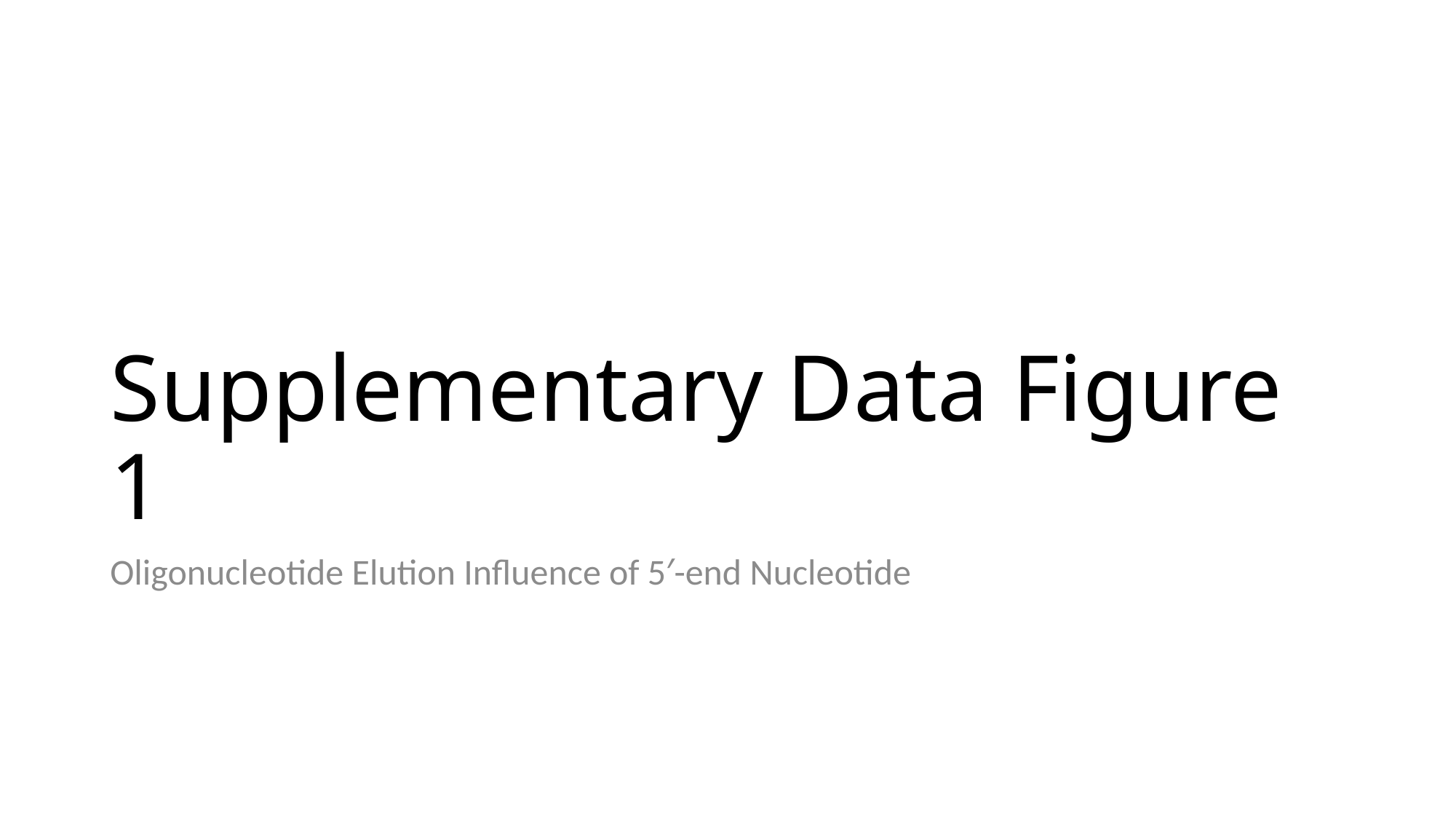

# Supplementary Data Figure 1
Oligonucleotide Elution Influence of 5′-end Nucleotide

## Slide 2
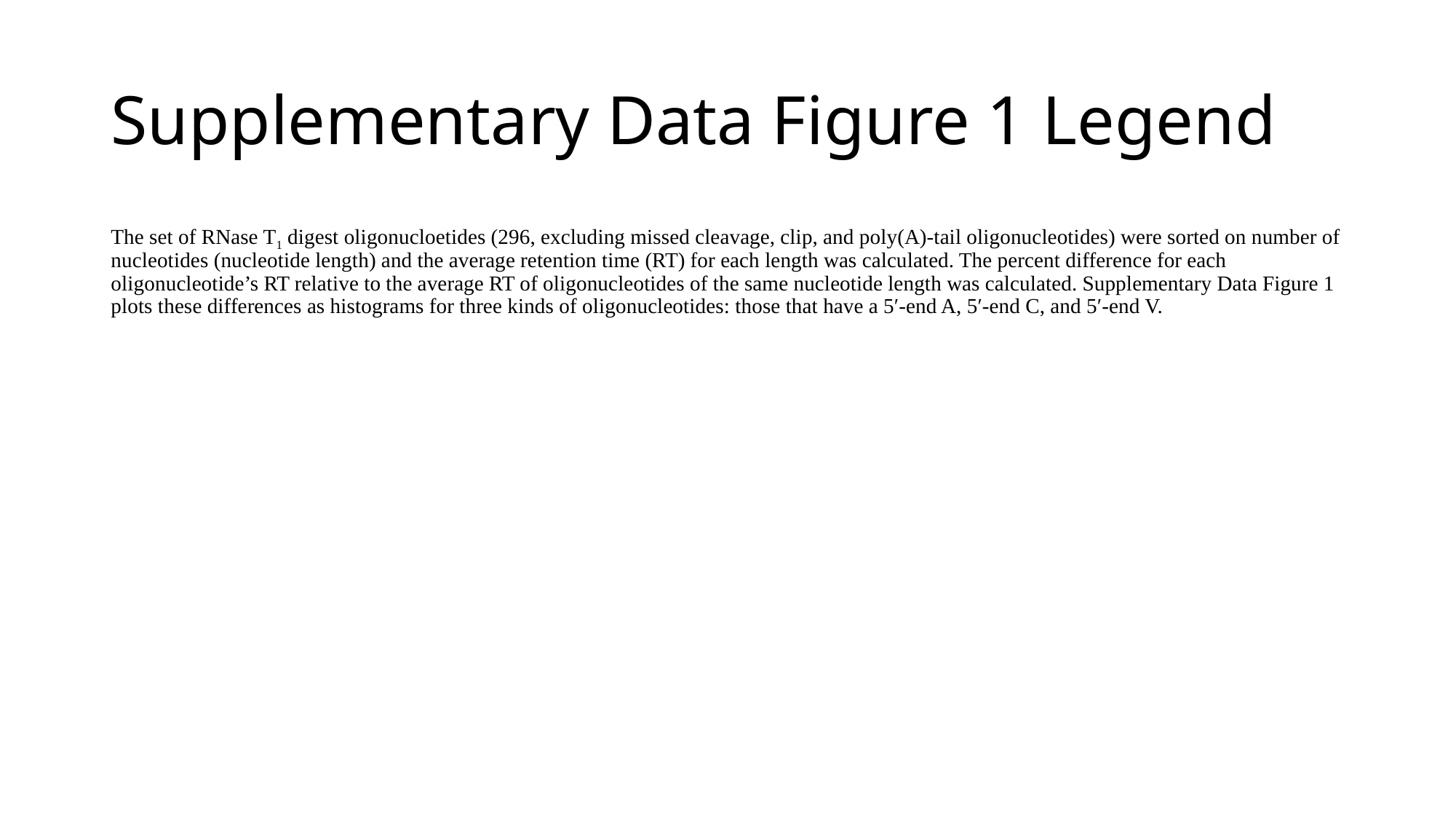

# Supplementary Data Figure 1 Legend
The set of RNase T1 digest oligonucloetides (296, excluding missed cleavage, clip, and poly(A)-tail oligonucleotides) were sorted on number of nucleotides (nucleotide length) and the average retention time (RT) for each length was calculated. The percent difference for each oligonucleotide’s RT relative to the average RT of oligonucleotides of the same nucleotide length was calculated. Supplementary Data Figure 1 plots these differences as histograms for three kinds of oligonucleotides: those that have a 5′-end A, 5′-end C, and 5′-end V.

## Slide 3
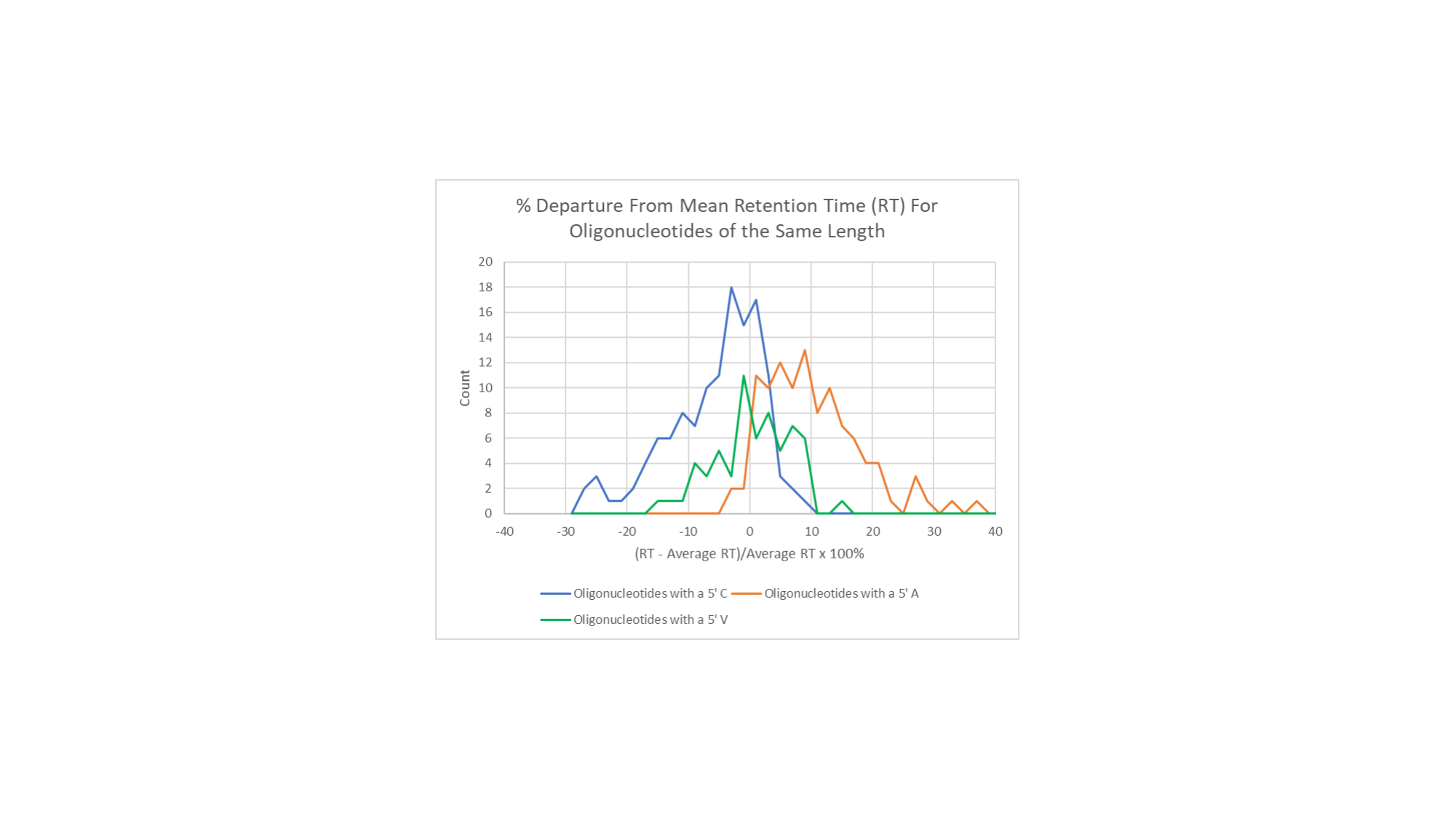

## Slide 4
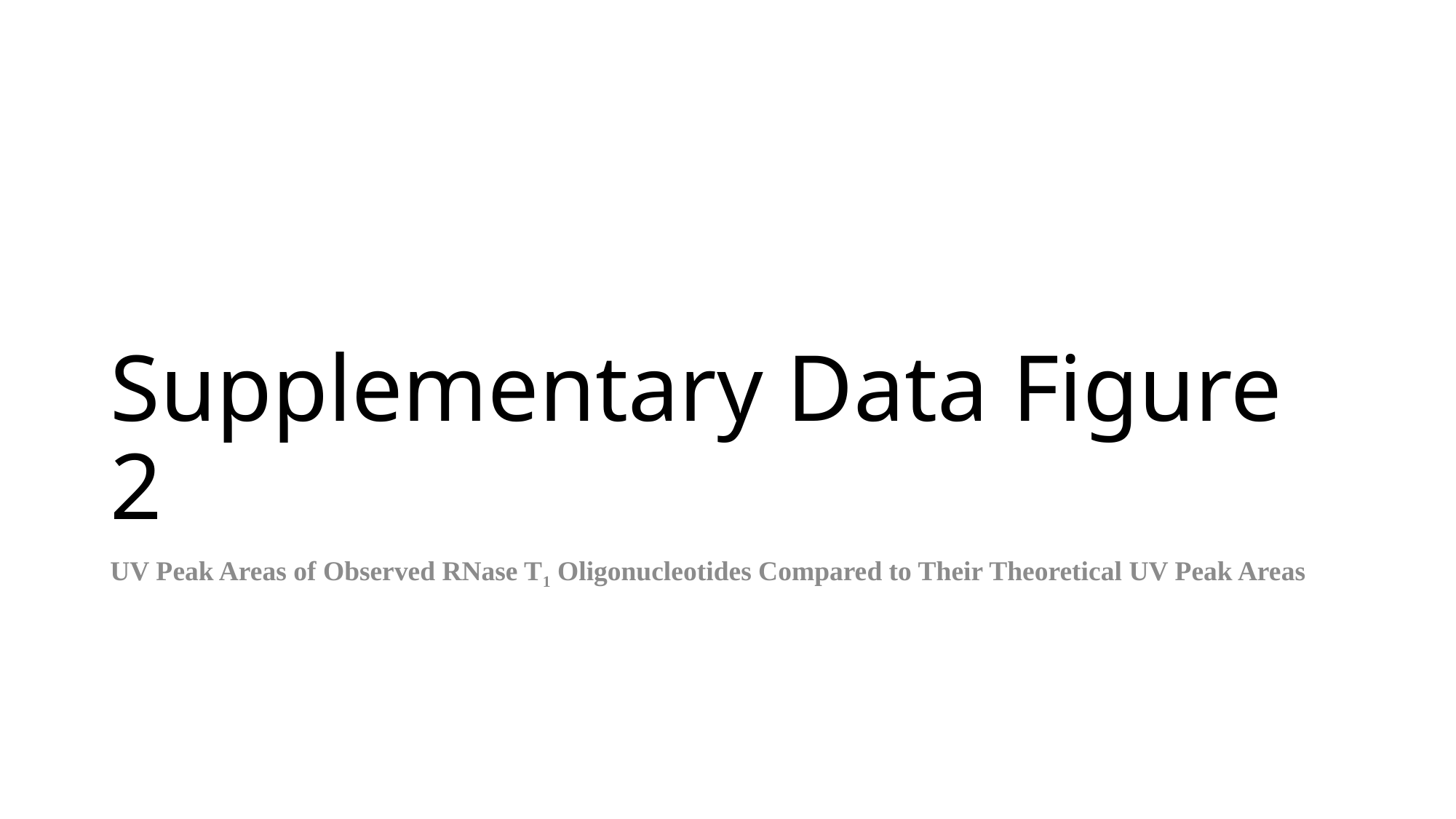

# Supplementary Data Figure 2
UV Peak Areas of Observed RNase T1 Oligonucleotides Compared to Their Theoretical UV Peak Areas

## Slide 5
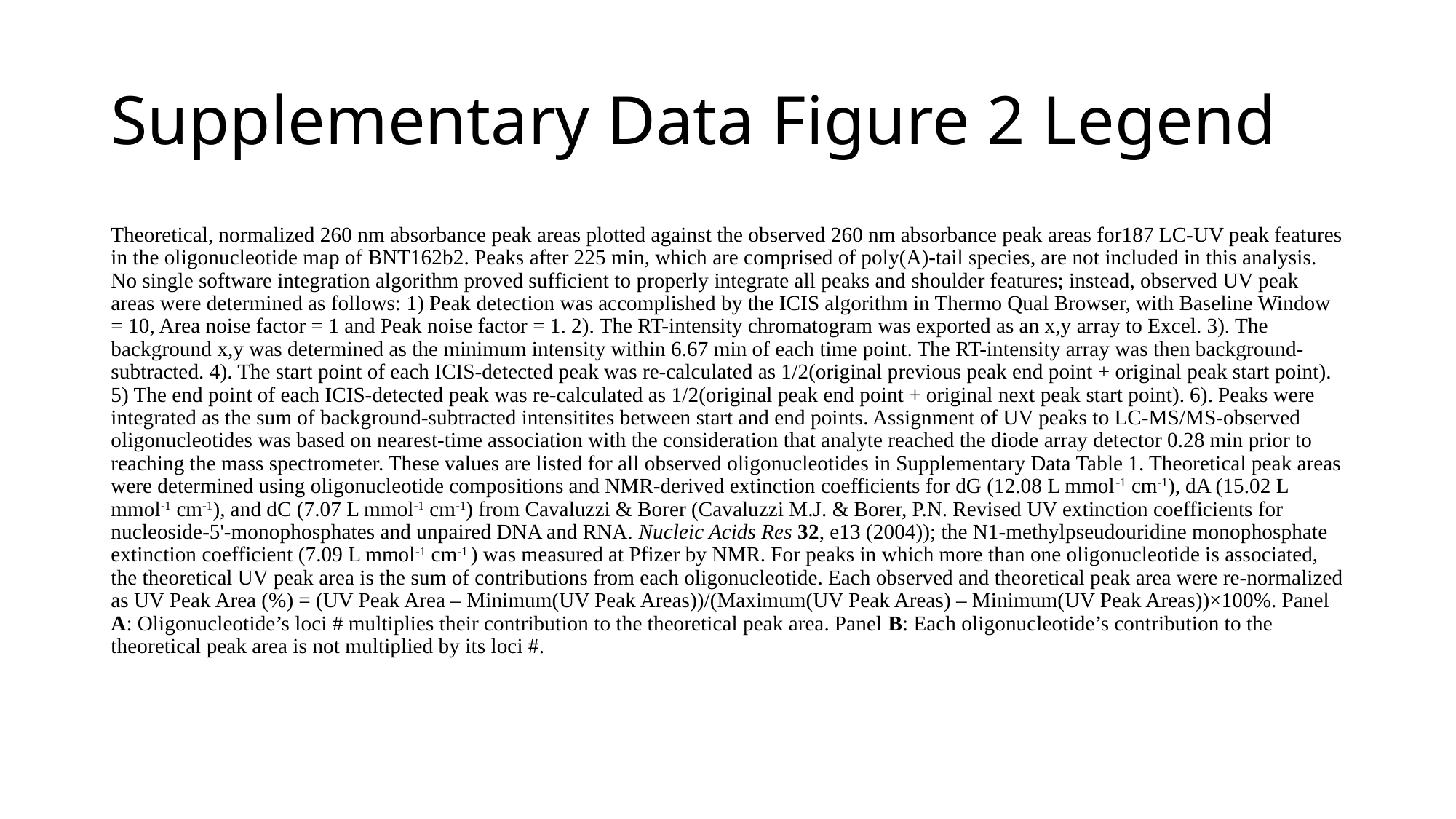

# Supplementary Data Figure 2 Legend
Theoretical, normalized 260 nm absorbance peak areas plotted against the observed 260 nm absorbance peak areas for187 LC-UV peak features in the oligonucleotide map of BNT162b2. Peaks after 225 min, which are comprised of poly(A)-tail species, are not included in this analysis. No single software integration algorithm proved sufficient to properly integrate all peaks and shoulder features; instead, observed UV peak areas were determined as follows: 1) Peak detection was accomplished by the ICIS algorithm in Thermo Qual Browser, with Baseline Window = 10, Area noise factor = 1 and Peak noise factor = 1. 2). The RT-intensity chromatogram was exported as an x,y array to Excel. 3). The background x,y was determined as the minimum intensity within 6.67 min of each time point. The RT-intensity array was then background-subtracted. 4). The start point of each ICIS-detected peak was re-calculated as 1/2(original previous peak end point + original peak start point). 5) The end point of each ICIS-detected peak was re-calculated as 1/2(original peak end point + original next peak start point). 6). Peaks were integrated as the sum of background-subtracted intensitites between start and end points. Assignment of UV peaks to LC-MS/MS-observed oligonucleotides was based on nearest-time association with the consideration that analyte reached the diode array detector 0.28 min prior to reaching the mass spectrometer. These values are listed for all observed oligonucleotides in Supplementary Data Table 1. Theoretical peak areas were determined using oligonucleotide compositions and NMR-derived extinction coefficients for dG (12.08 L mmol-1 cm-1), dA (15.02 L mmol-1 cm-1), and dC (7.07 L mmol-1 cm-1) from Cavaluzzi & Borer (Cavaluzzi M.J. & Borer, P.N. Revised UV extinction coefficients for nucleoside-5'-monophosphates and unpaired DNA and RNA. Nucleic Acids Res 32, e13 (2004)); the N1-methylpseudouridine monophosphate extinction coefficient (7.09 L mmol-1 cm-1 ) was measured at Pfizer by NMR. For peaks in which more than one oligonucleotide is associated, the theoretical UV peak area is the sum of contributions from each oligonucleotide. Each observed and theoretical peak area were re-normalized as UV Peak Area (%) = (UV Peak Area – Minimum(UV Peak Areas))/(Maximum(UV Peak Areas) – Minimum(UV Peak Areas))×100%. Panel A: Oligonucleotide’s loci # multiplies their contribution to the theoretical peak area. Panel B: Each oligonucleotide’s contribution to the theoretical peak area is not multiplied by its loci #.

## Slide 6
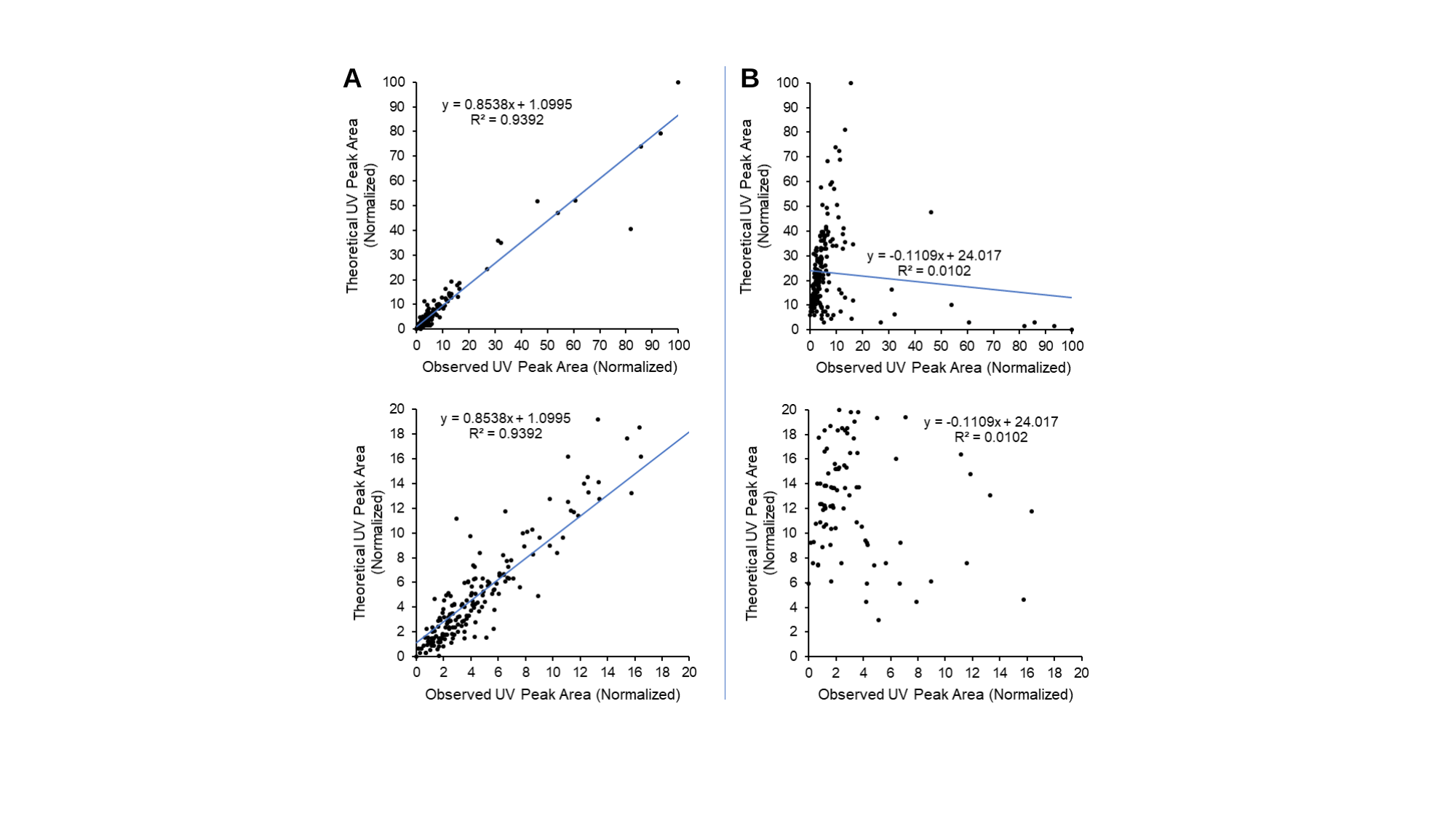

A
B

## Slide 7
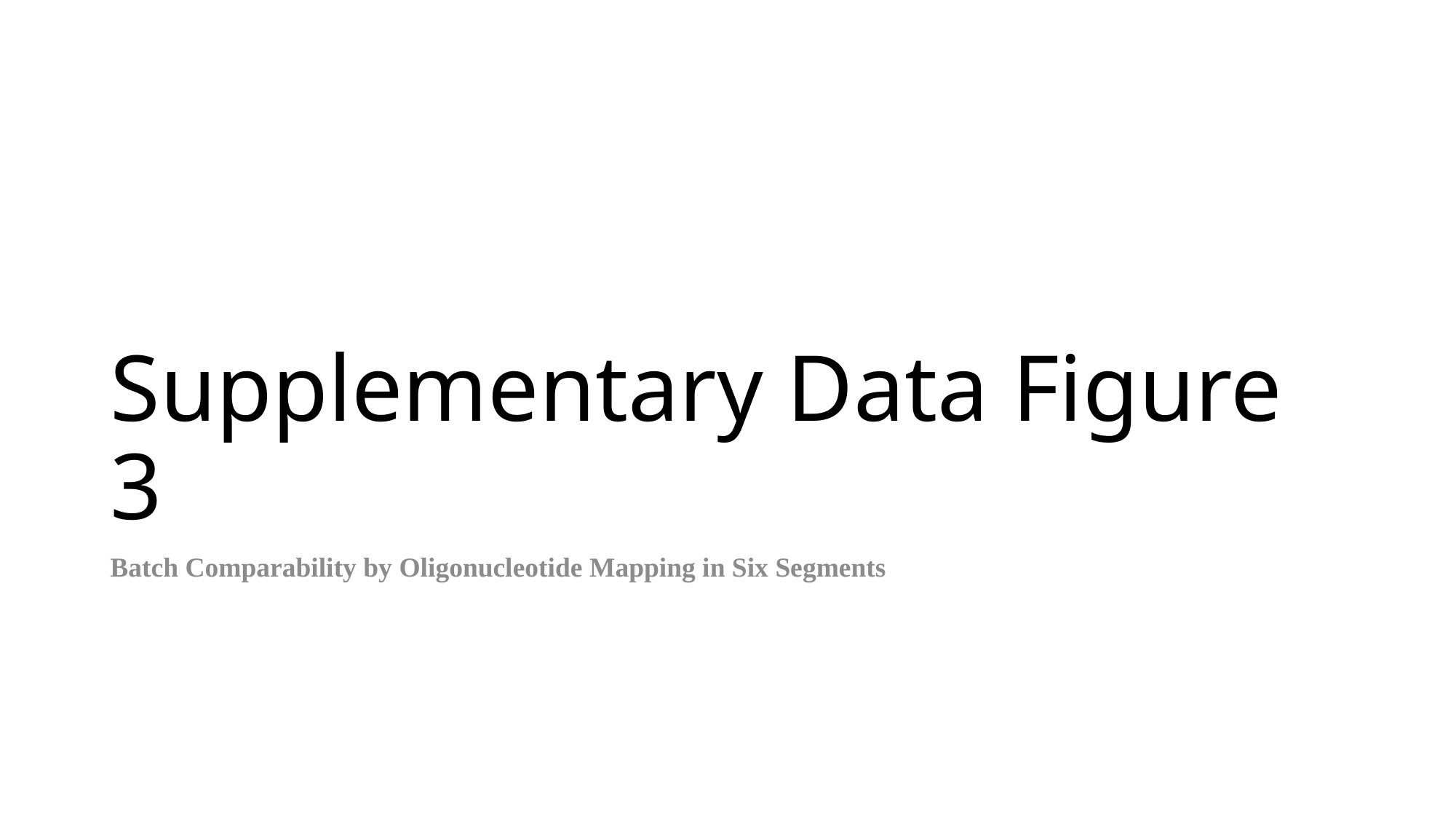

# Supplementary Data Figure 3
Batch Comparability by Oligonucleotide Mapping in Six Segments

## Slide 8
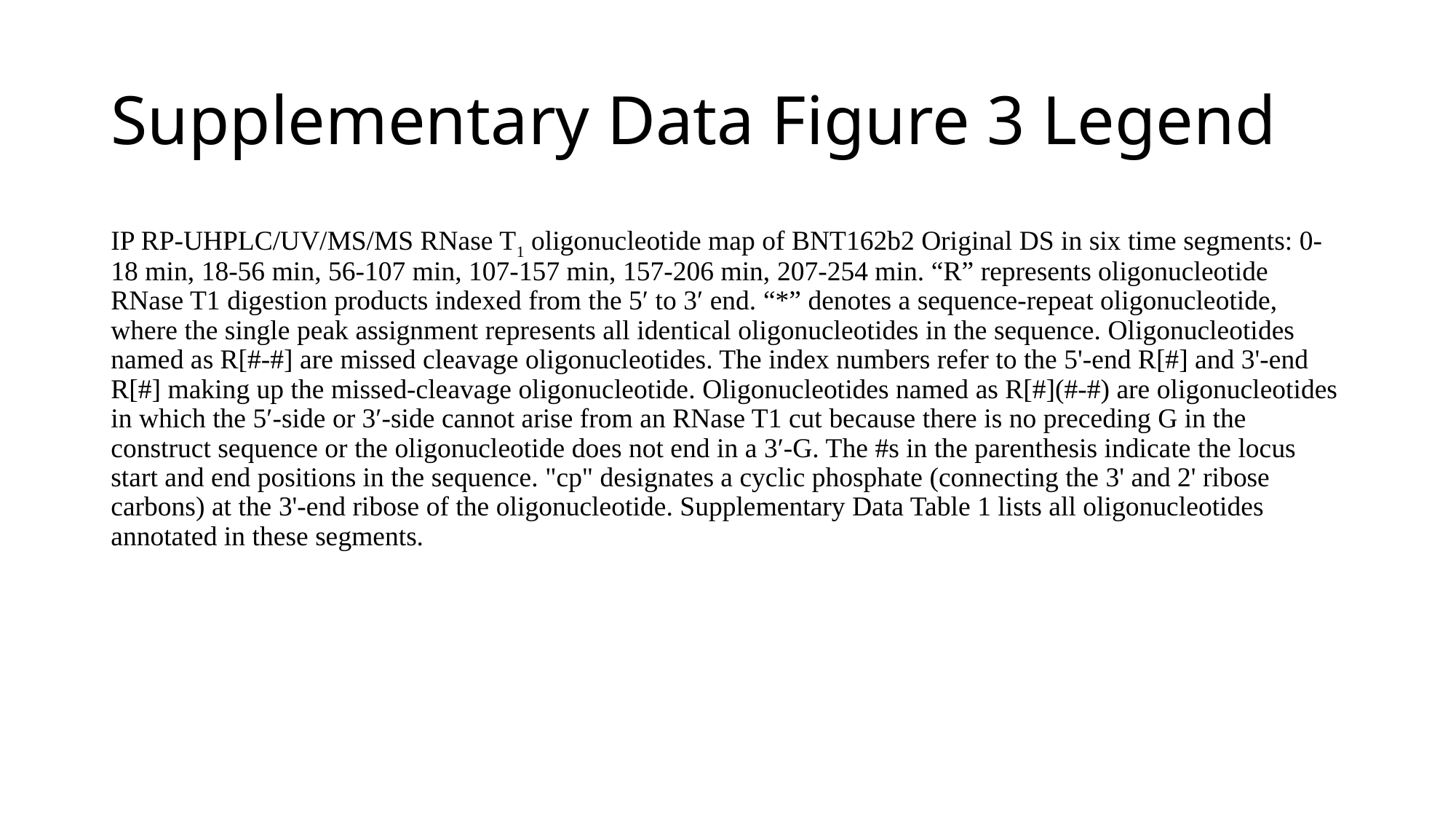

# Supplementary Data Figure 3 Legend
IP RP-UHPLC/UV/MS/MS RNase T1 oligonucleotide map of BNT162b2 Original DS in six time segments: 0-18 min, 18-56 min, 56-107 min, 107-157 min, 157-206 min, 207-254 min. “R” represents oligonucleotide RNase T1 digestion products indexed from the 5′ to 3′ end. “*” denotes a sequence-repeat oligonucleotide, where the single peak assignment represents all identical oligonucleotides in the sequence. Oligonucleotides named as R[#-#] are missed cleavage oligonucleotides. The index numbers refer to the 5'-end R[#] and 3'-end R[#] making up the missed-cleavage oligonucleotide. Oligonucleotides named as R[#](#-#) are oligonucleotides in which the 5′-side or 3′-side cannot arise from an RNase T1 cut because there is no preceding G in the construct sequence or the oligonucleotide does not end in a 3′-G. The #s in the parenthesis indicate the locus start and end positions in the sequence. "cp" designates a cyclic phosphate (connecting the 3' and 2' ribose carbons) at the 3'-end ribose of the oligonucleotide. Supplementary Data Table 1 lists all oligonucleotides annotated in these segments.

## Slide 9
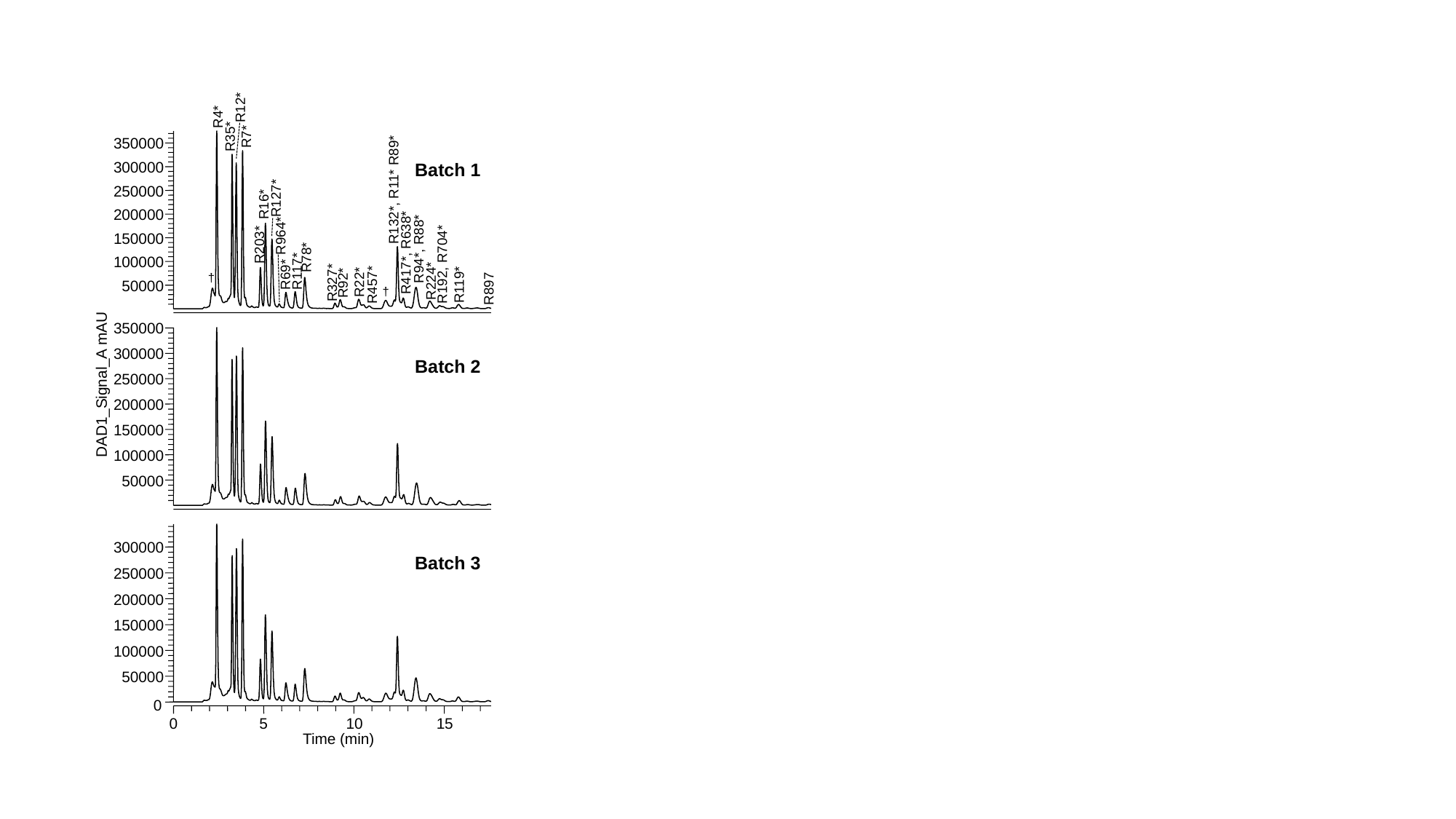

R12*
R4*
R35*
R7*
350000
300000
250000
200000
150000
100000
50000
R132*, R11* R89*
Batch 1
R127*
R16*
R417*, R638*
R94*, R88*
R964*
R192, R704*
R203*
R78*
R117*
R69*
R224*
R327*
R119*
R457*
R22*
R92*
†
R897
†
350000
300000
250000
200000
150000
100000
50000
Batch 2
DAD1_Signal_A mAU
300000
250000
200000
150000
100000
50000
0
0
5
10
15
Time (min)
Batch 3

## Slide 10
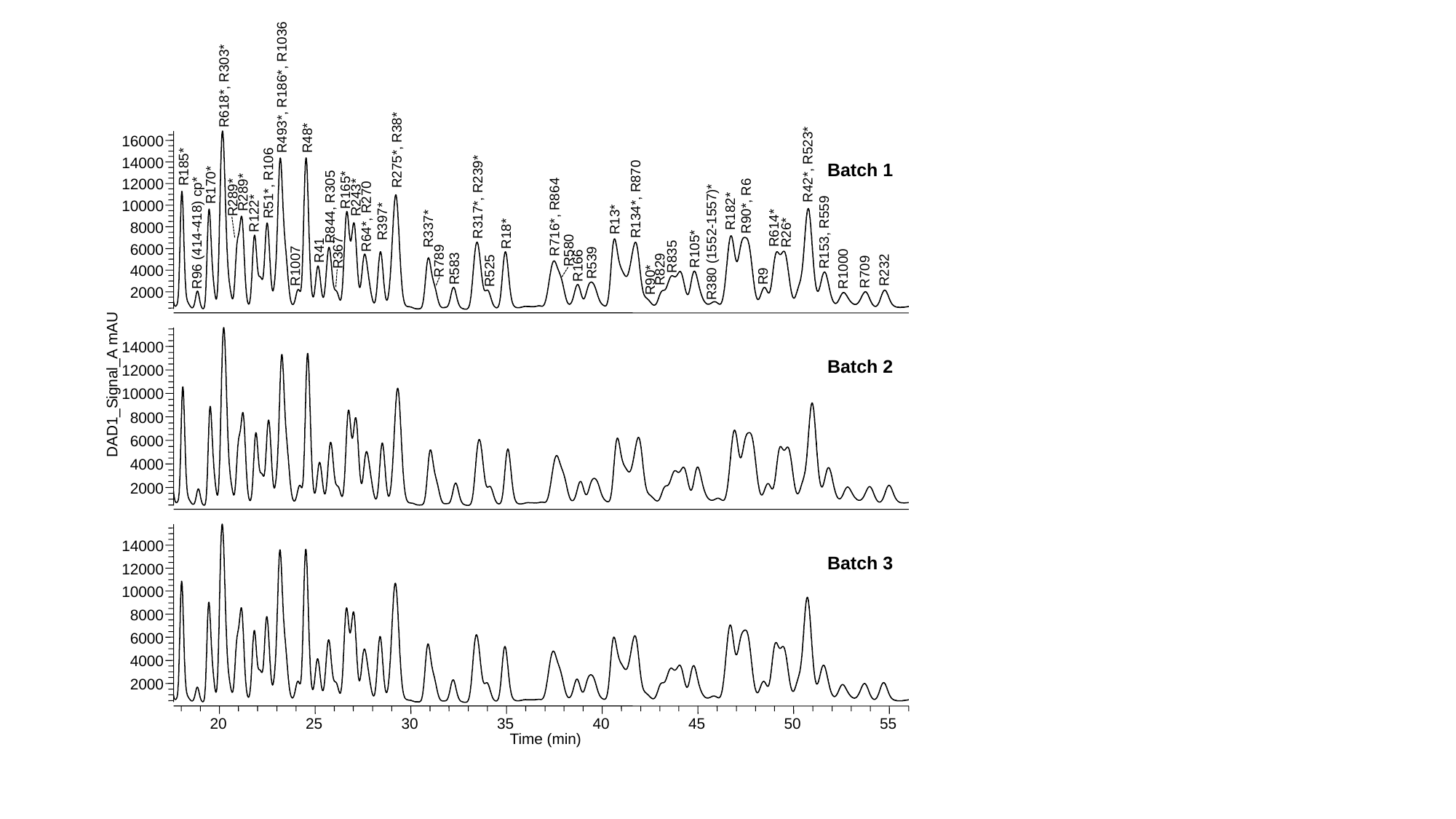

R493*, R186*, R1036
R618*, R303*
R275*, R38*
R48*
R42*, R523*
16000
14000
12000
10000
8000
6000
4000
2000
R51*, R106
R185*
Batch 1
R317*, R239*
R134*, R870
R170*
R844, R305
R165*
R96 (414-418) cp*
R289*
R716*, R864
R90*, R6
R289*
R243*
R64*, R270
R380 (1552-1557)*
R182*
R122*
R153, R559
R397*
R13*
R614*
R337*
R26*
R18*
R105*
R580
R367
R41
R835
R789
R1007
R539
R1000
R166
R583
R829
R232
R525
R709
R90*
R9
14000
12000
10000
8000
6000
4000
2000
Batch 2
DAD1_Signal_A mAU
14000
12000
10000
8000
6000
4000
2000
20
25
30
35
40
45
50
55
Time (min)
Batch 3

## Slide 11
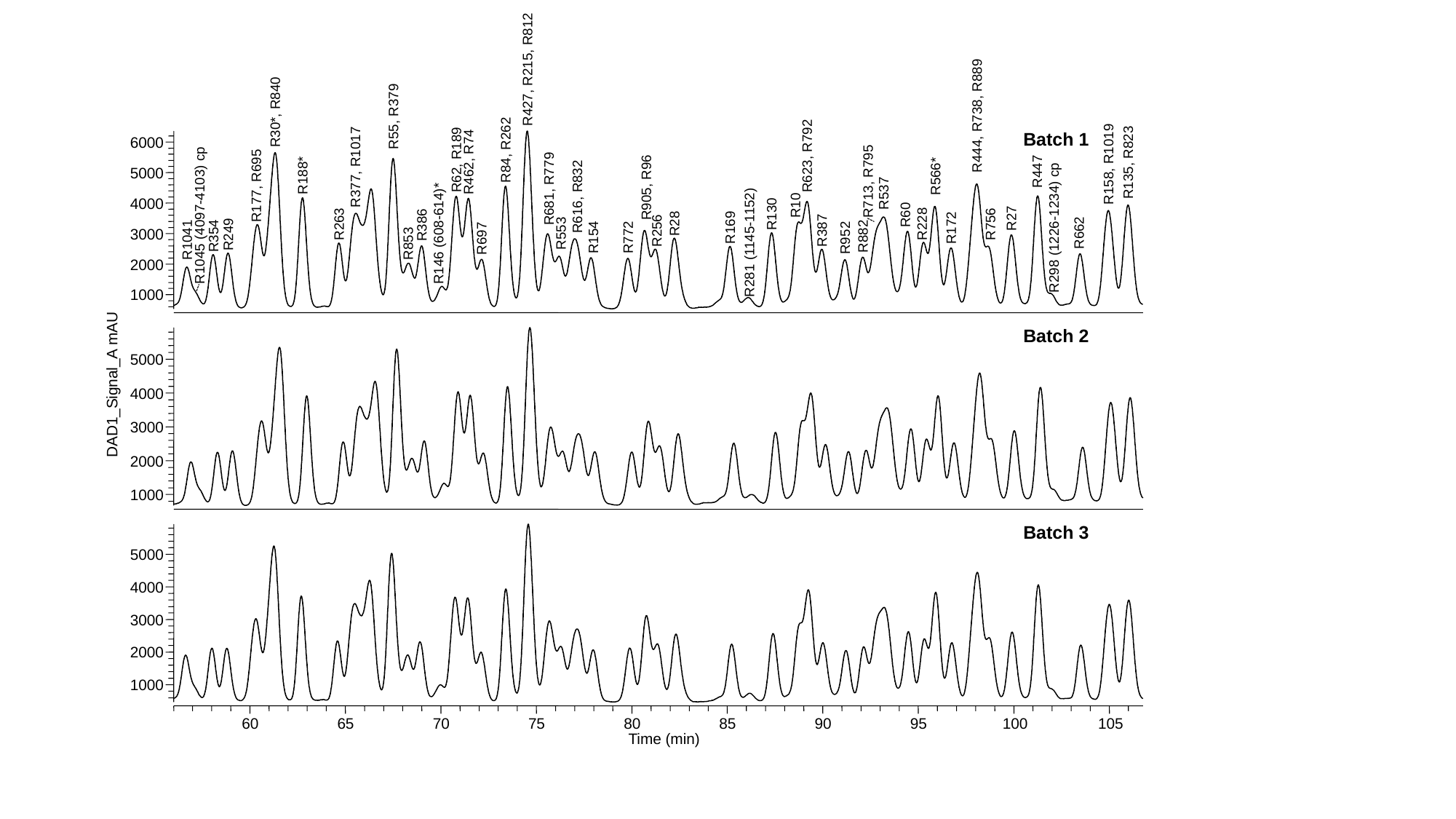

R427, R215, R812
R444, R738, R889
R30*, R840
R55, R379
R84, R262
R623, R792
Batch 1
R158, R1019
R135, R823
R377, R1017
R62, R189
R462, R74
6000
5000
4000
3000
2000
1000
R713, R795
R1045 (4097-4103) cp
R177, R695
R681, R779
R905, R96
R447
R188*
R566*
R616, R832
R298 (1226-1234) cp
R537
R146 (608-614)*
R281 (1145-1152)
R10
R130
R60
R27
R228
R263
R756
R386
R28
R169
R172
R387
R256
R662
R553
R249
R354
R1041
R882
R772
R154
R952
R697
R853
Batch 2
5000
4000
3000
2000
1000
DAD1_Signal_A mAU
Batch 3
5000
4000
3000
2000
1000
60
65
70
75
80
85
90
95
100
105
Time (min)

## Slide 12
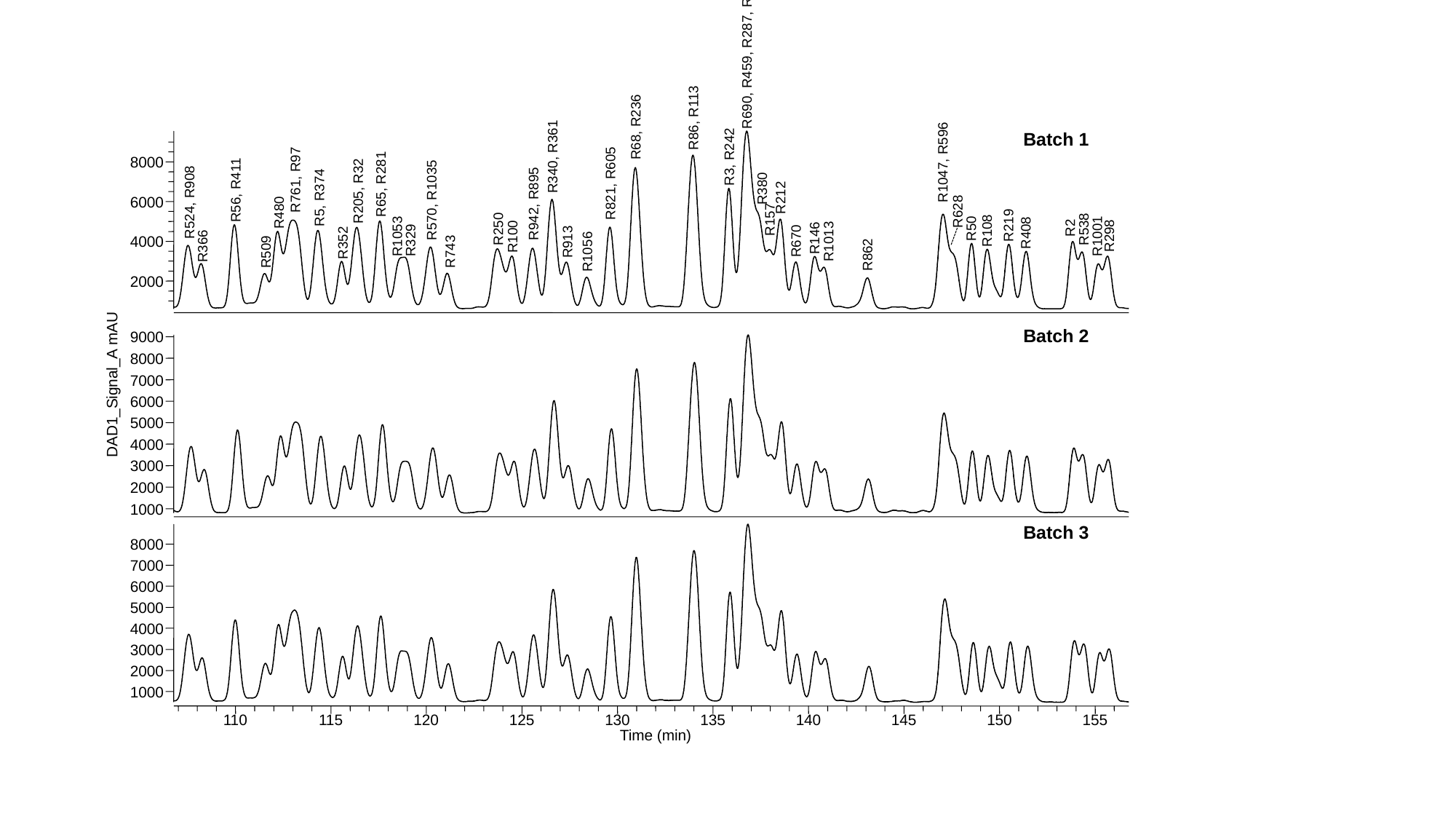

R690, R459, R287, R380
R86, R113
R68, R236
R340, R361
R1047, R596
Batch 1
R3, R242
8000
6000
4000
2000
R821, R605
R761, R97
R65, R281
R56, R411
R205, R32
R570, R1035
R524, R908
R942, R895
R5, R374
R380
R212
R628
R480
R157
R219
R250
R538
R108
R50
R1001
R1053
R408
R2
R298
R100
R1013
R146
R329
R670
R913
R352
R366
R1056
R743
R509
R862
Batch 2
9000
8000
7000
6000
5000
4000
3000
2000
1000
DAD1_Signal_A mAU
Batch 3
8000
7000
6000
5000
4000
3000
2000
1000
110
115
120
125
130
135
140
145
150
155
Time (min)

## Slide 13
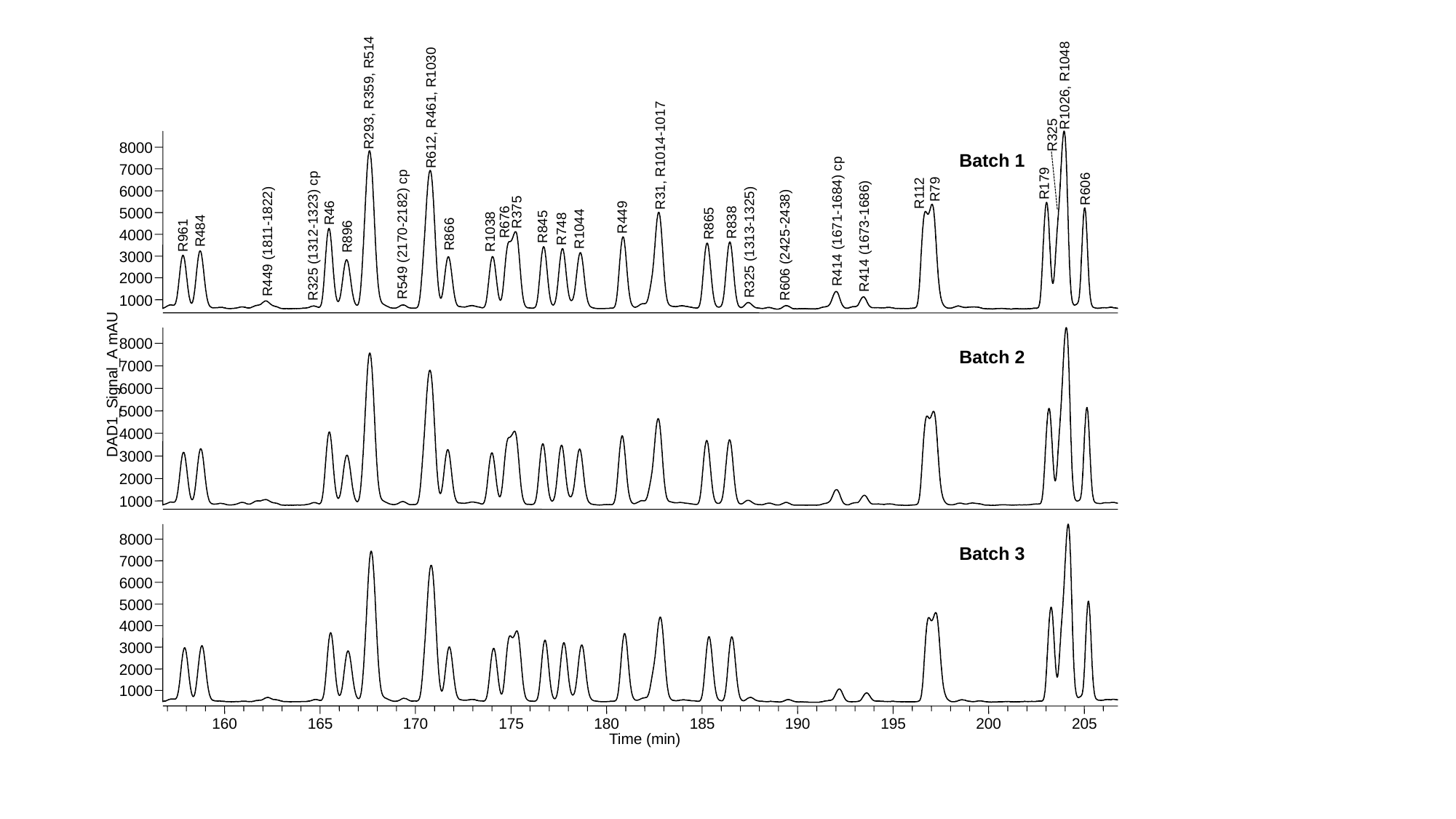

R293, R359, R514
R1026, R1048
R612, R461, R1030
R31, R1014-1017
R325
8000
7000
6000
5000
4000
3000
2000
1000
Batch 1
R414 (1671-1684) cp
R179
R606
R112
R79
R414 (1673-1686)
R449 (1811-1822)
R375
R46
R449
R676
R838
R865
R1044
R845
R1038
R748
R484
R866
R896
R961
R549 (2170-2182) cp
R325 (1312-1323) cp
R325 (1313-1325)
R606 (2425-2438)
8000
7000
6000
5000
4000
3000
2000
1000
Batch 2
DAD1_Signal_A mAU
8000
7000
6000
5000
4000
3000
2000
1000
160
165
170
175
180
185
190
195
200
205
Time (min)
Batch 3

## Slide 14
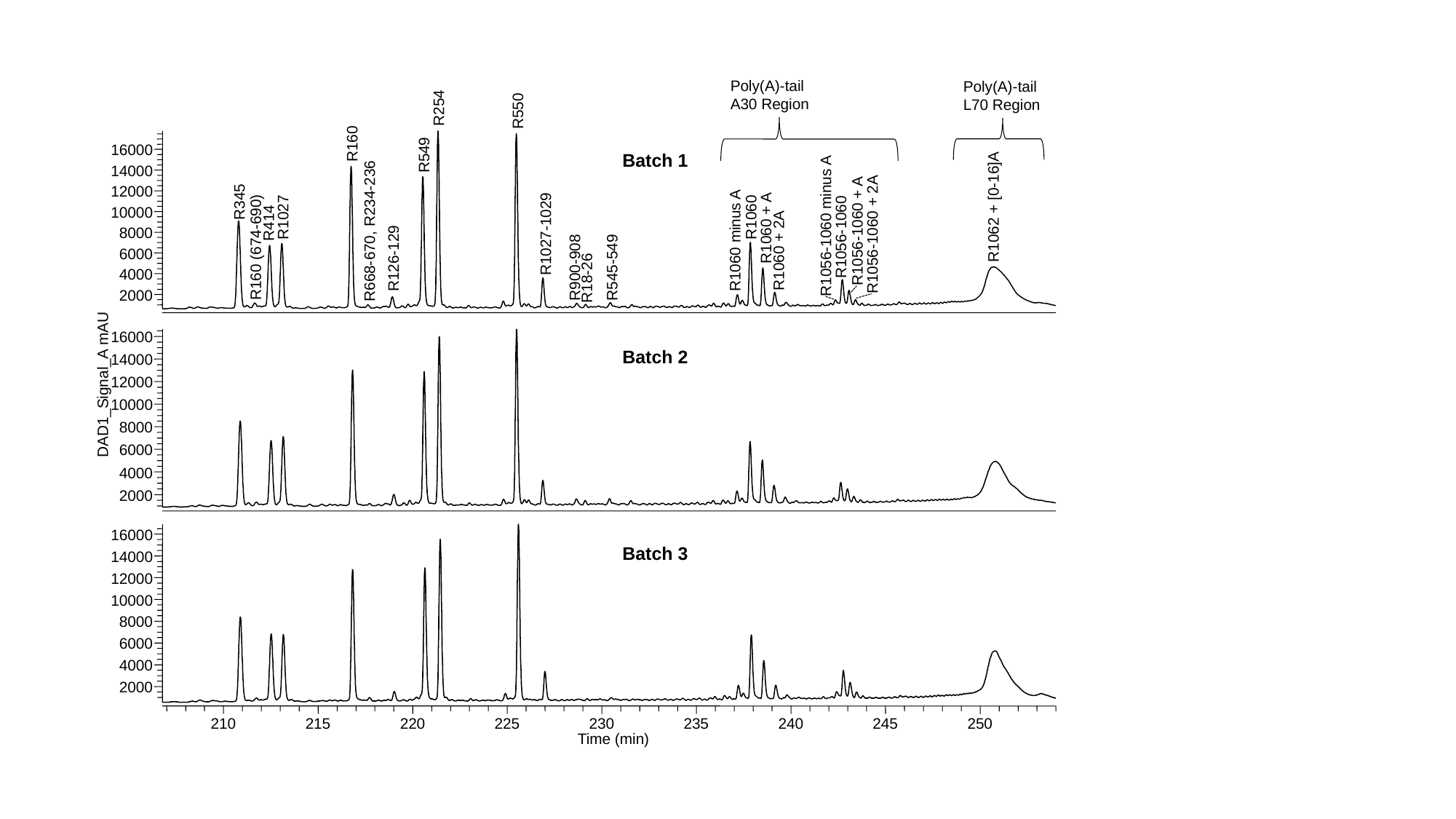

Poly(A)-tail A30 Region
Poly(A)-tail L70 Region
R254
R550
R160
16000
14000
12000
10000
8000
6000
4000
2000
R549
Batch 1
R1062 + [0-16]A
R1056-1060 minus A
R668-670, R234-236
R1056-1060 + 2A
R1056-1060 + A
R345
R1060 minus A
R1060 + A
R1027-1029
R160 (674-690)
R1027
R1060
R1056-1060
R414
R1060 + 2A
R126-129
R900-908
R545-549
R18-26
16000
14000
12000
10000
8000
6000
4000
2000
Batch 2
DAD1_Signal_A mAU
16000
14000
12000
10000
8000
6000
4000
2000
210
215
220
225
230
235
240
245
250
Time (min)
Batch 3

## Slide 15
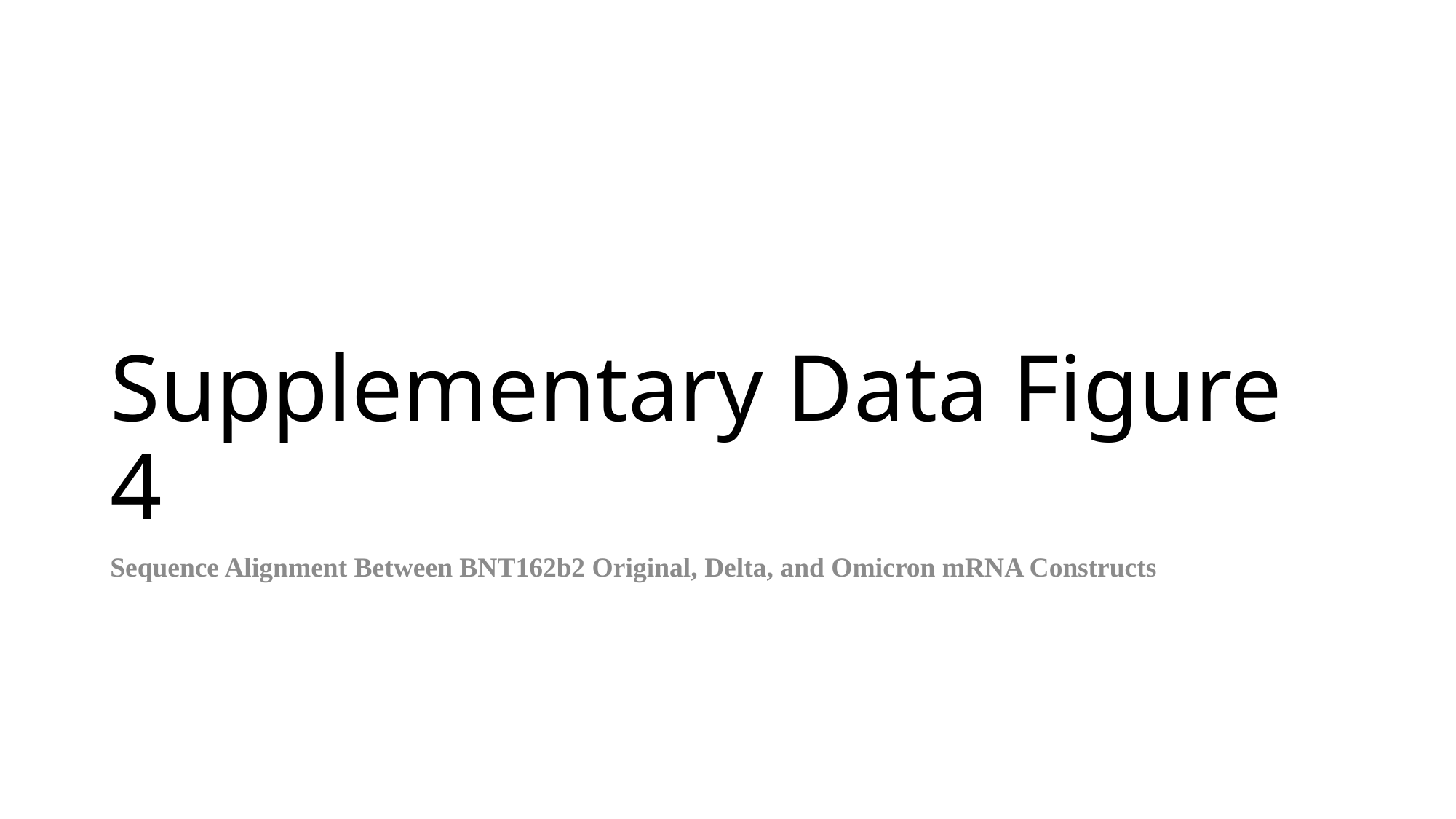

# Supplementary Data Figure 4
Sequence Alignment Between BNT162b2 Original, Delta, and Omicron mRNA Constructs

## Slide 16
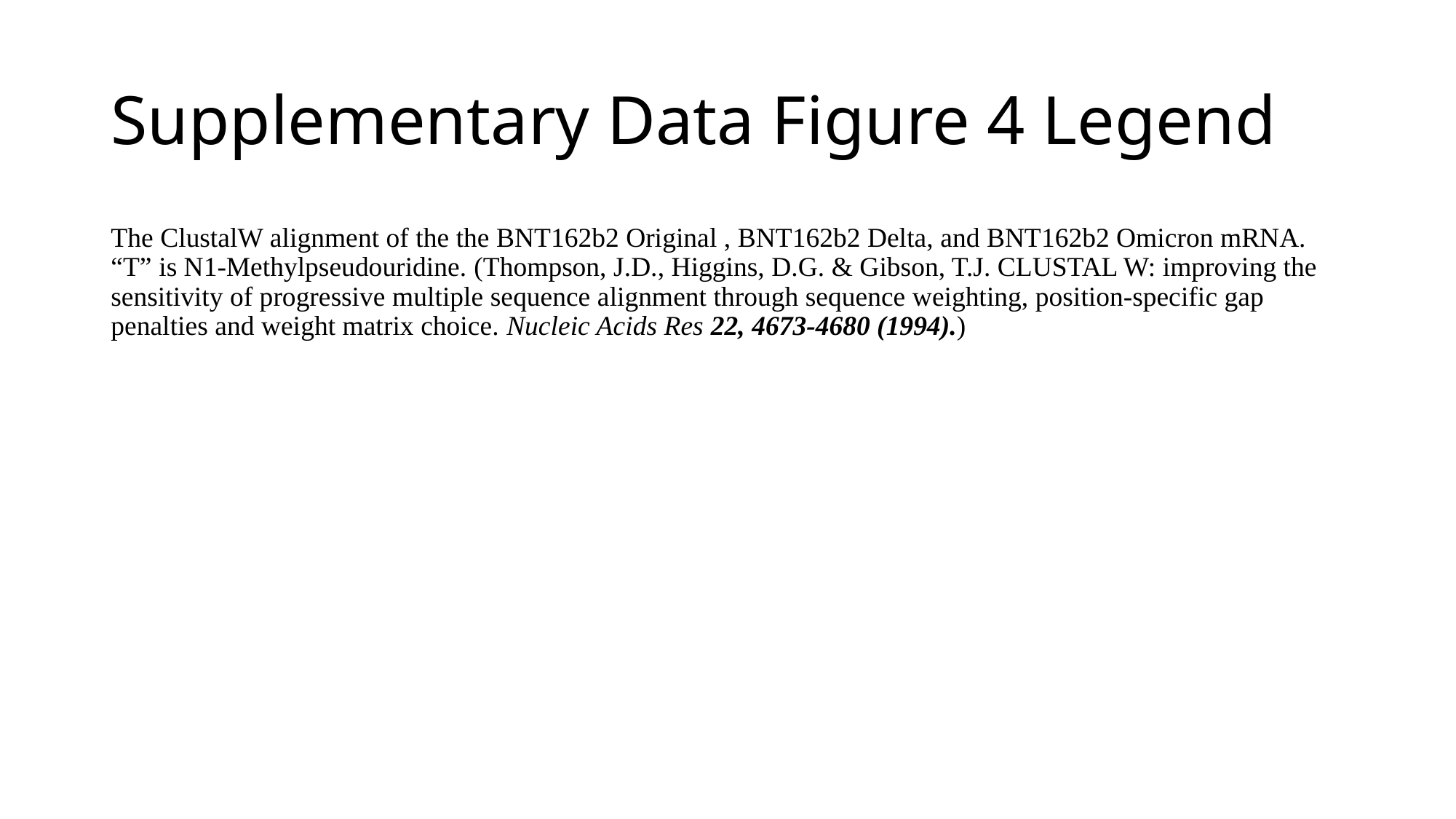

# Supplementary Data Figure 4 Legend
The ClustalW alignment of the the BNT162b2 Original , BNT162b2 Delta, and BNT162b2 Omicron mRNA. “T” is N1-Methylpseudouridine. (Thompson, J.D., Higgins, D.G. & Gibson, T.J. CLUSTAL W: improving the sensitivity of progressive multiple sequence alignment through sequence weighting, position-specific gap penalties and weight matrix choice. Nucleic Acids Res 22, 4673-4680 (1994).)

## Slide 17
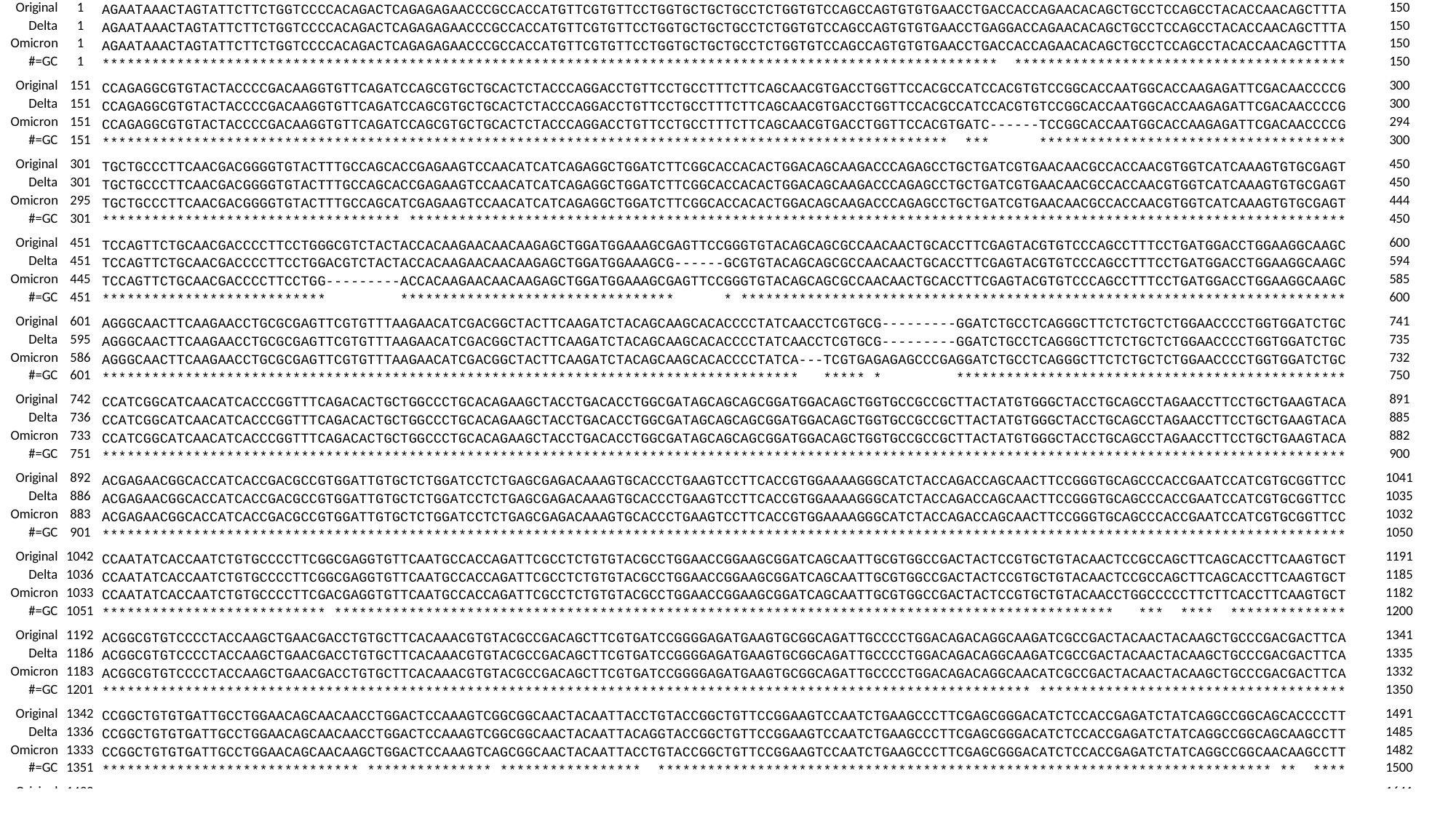

## Slide 18
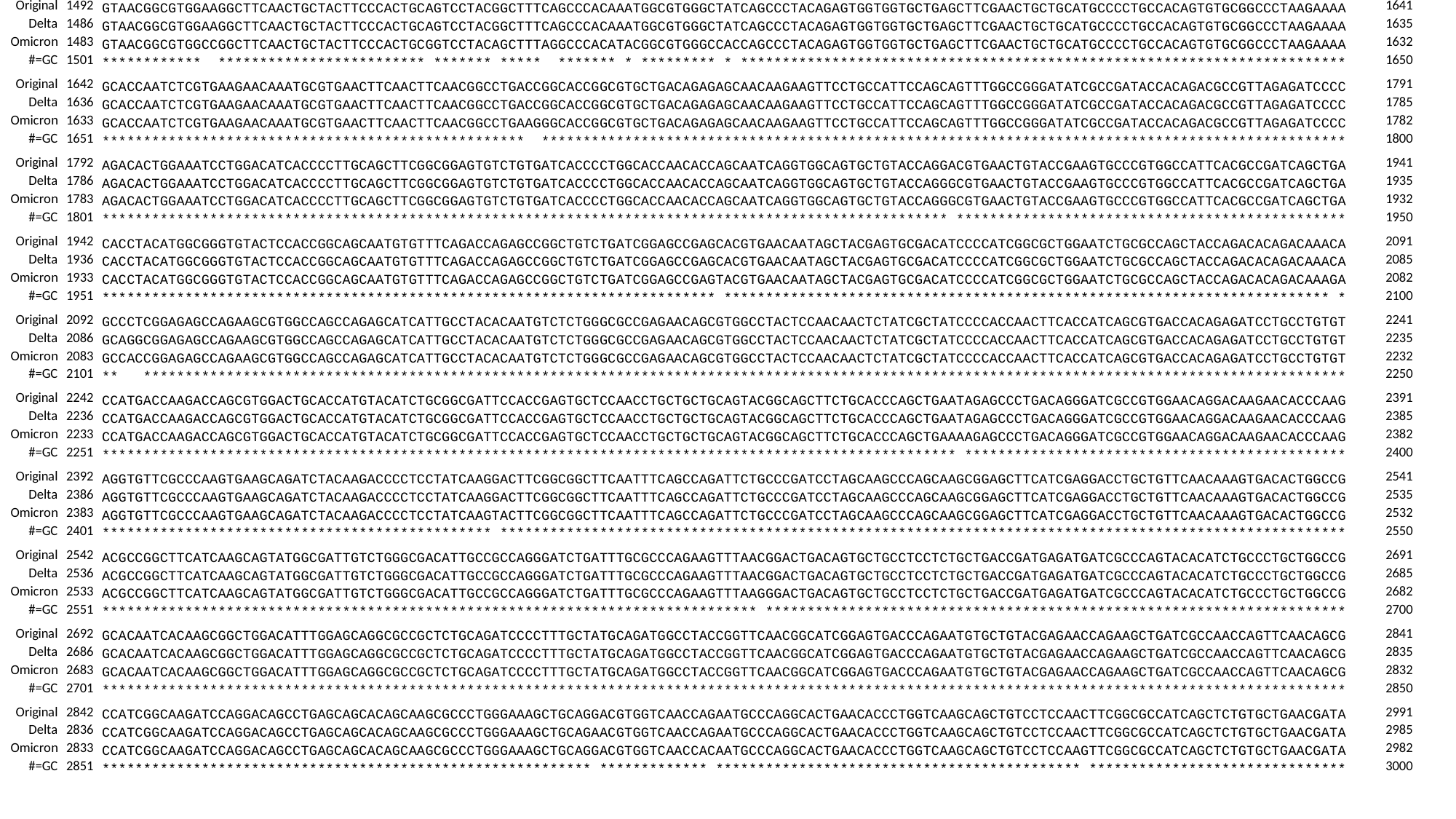

## Slide 19
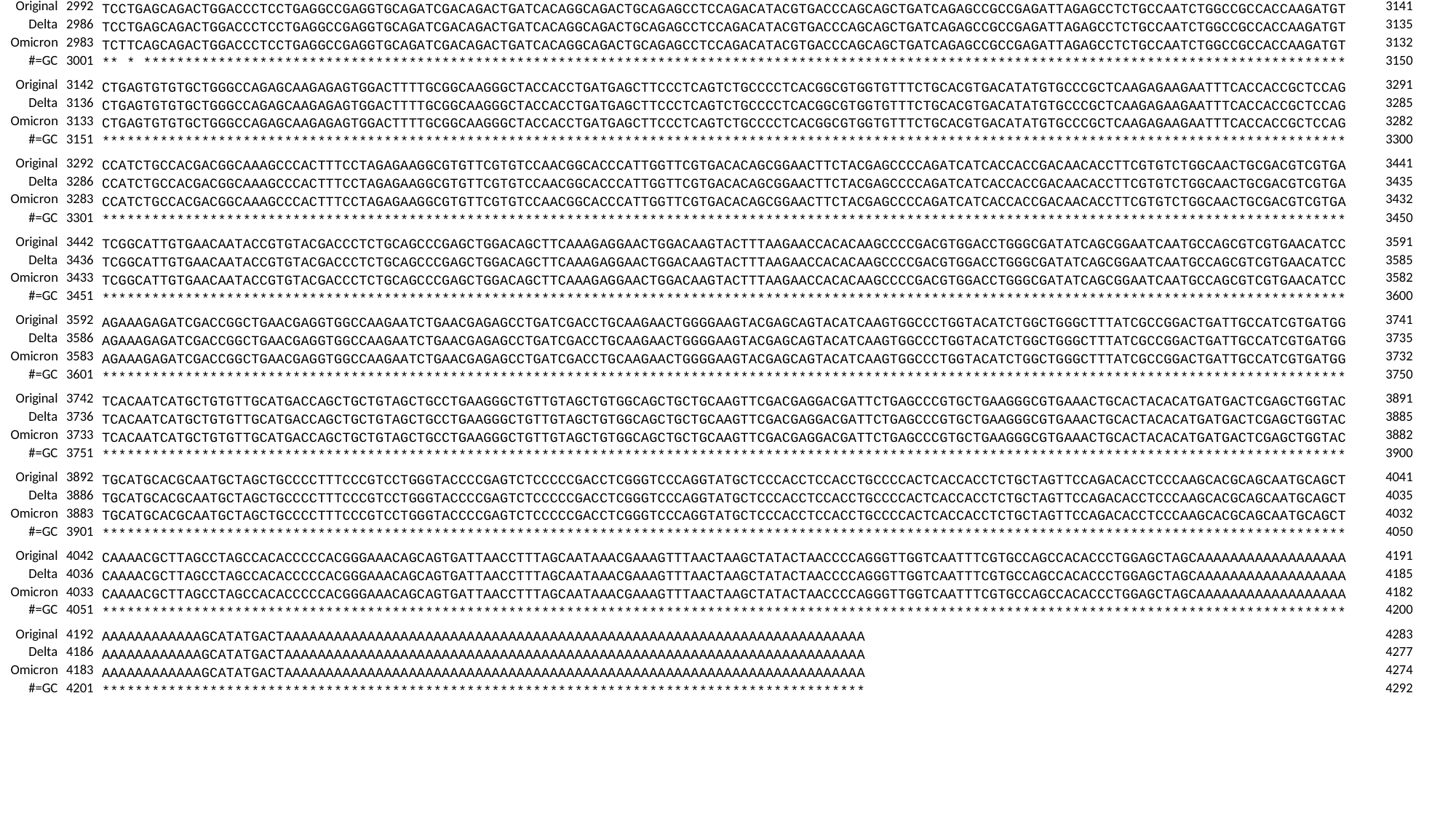

## Slide 20
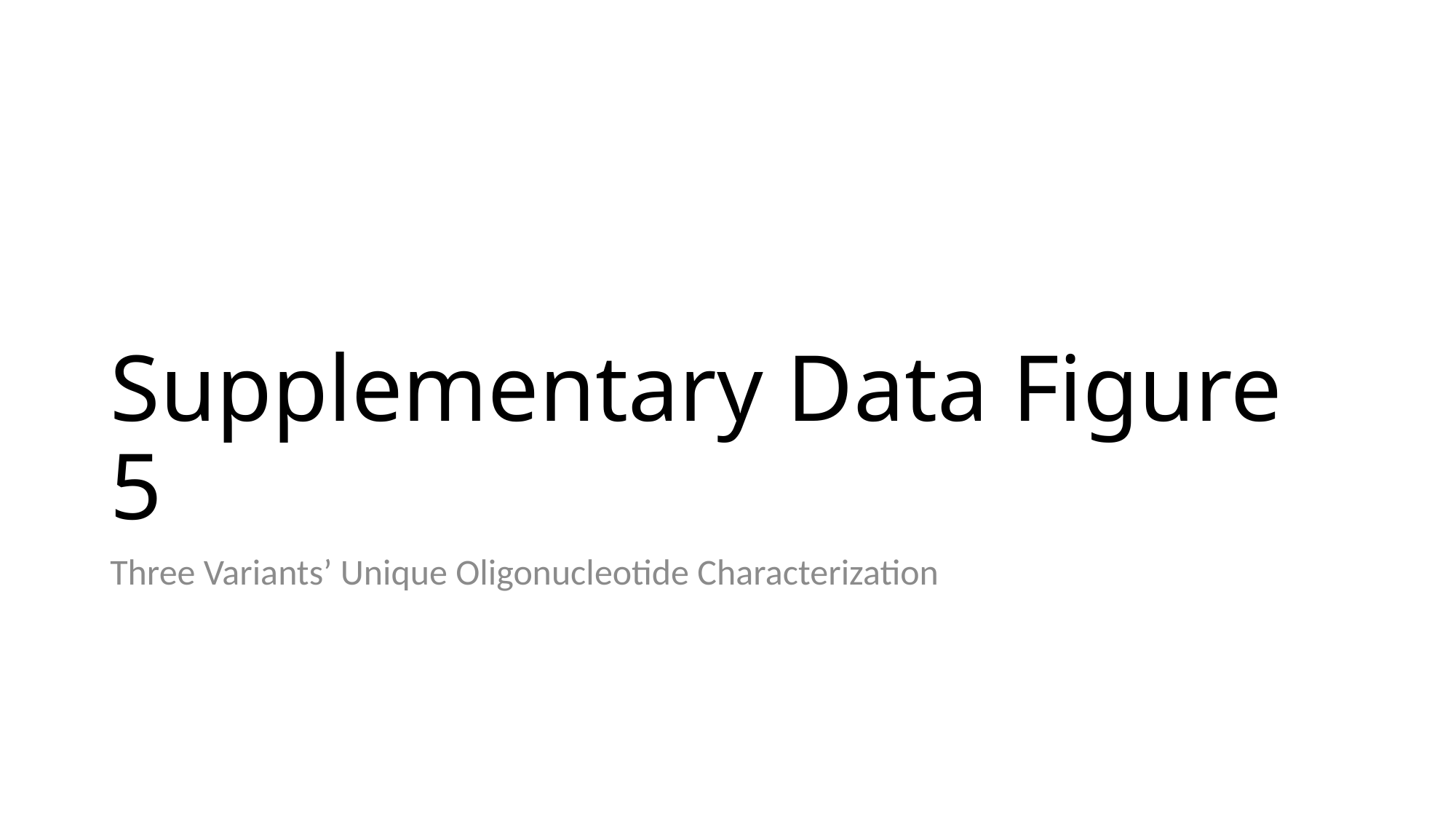

# Supplementary Data Figure 5
Three Variants’ Unique Oligonucleotide Characterization

## Slide 21
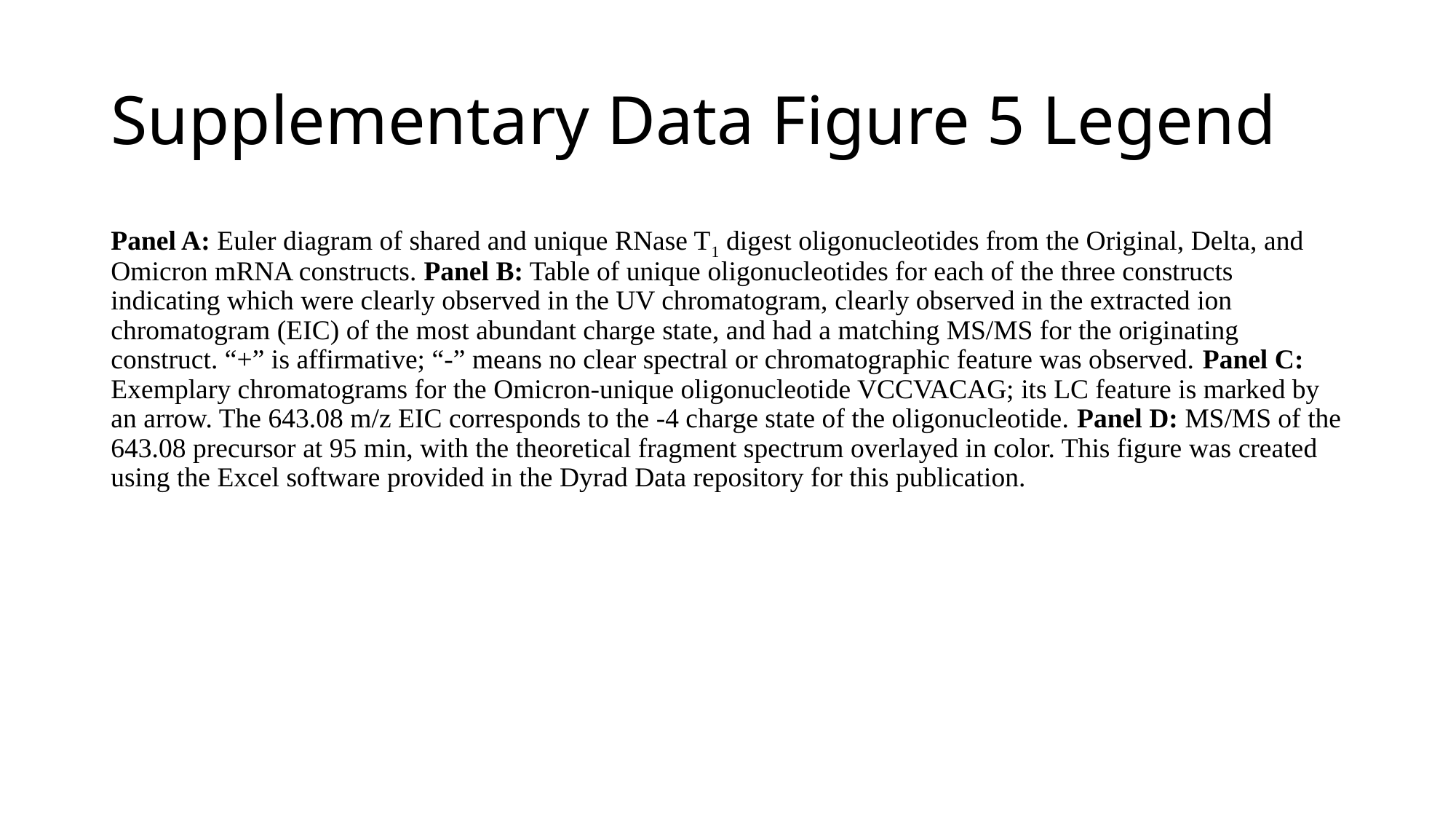

# Supplementary Data Figure 5 Legend
Panel A: Euler diagram of shared and unique RNase T1 digest oligonucleotides from the Original, Delta, and Omicron mRNA constructs. Panel B: Table of unique oligonucleotides for each of the three constructs indicating which were clearly observed in the UV chromatogram, clearly observed in the extracted ion chromatogram (EIC) of the most abundant charge state, and had a matching MS/MS for the originating construct. “+” is affirmative; “-” means no clear spectral or chromatographic feature was observed. Panel C: Exemplary chromatograms for the Omicron-unique oligonucleotide VCCVACAG; its LC feature is marked by an arrow. The 643.08 m/z EIC corresponds to the -4 charge state of the oligonucleotide. Panel D: MS/MS of the 643.08 precursor at 95 min, with the theoretical fragment spectrum overlayed in color. This figure was created using the Excel software provided in the Dyrad Data repository for this publication.

## Slide 22
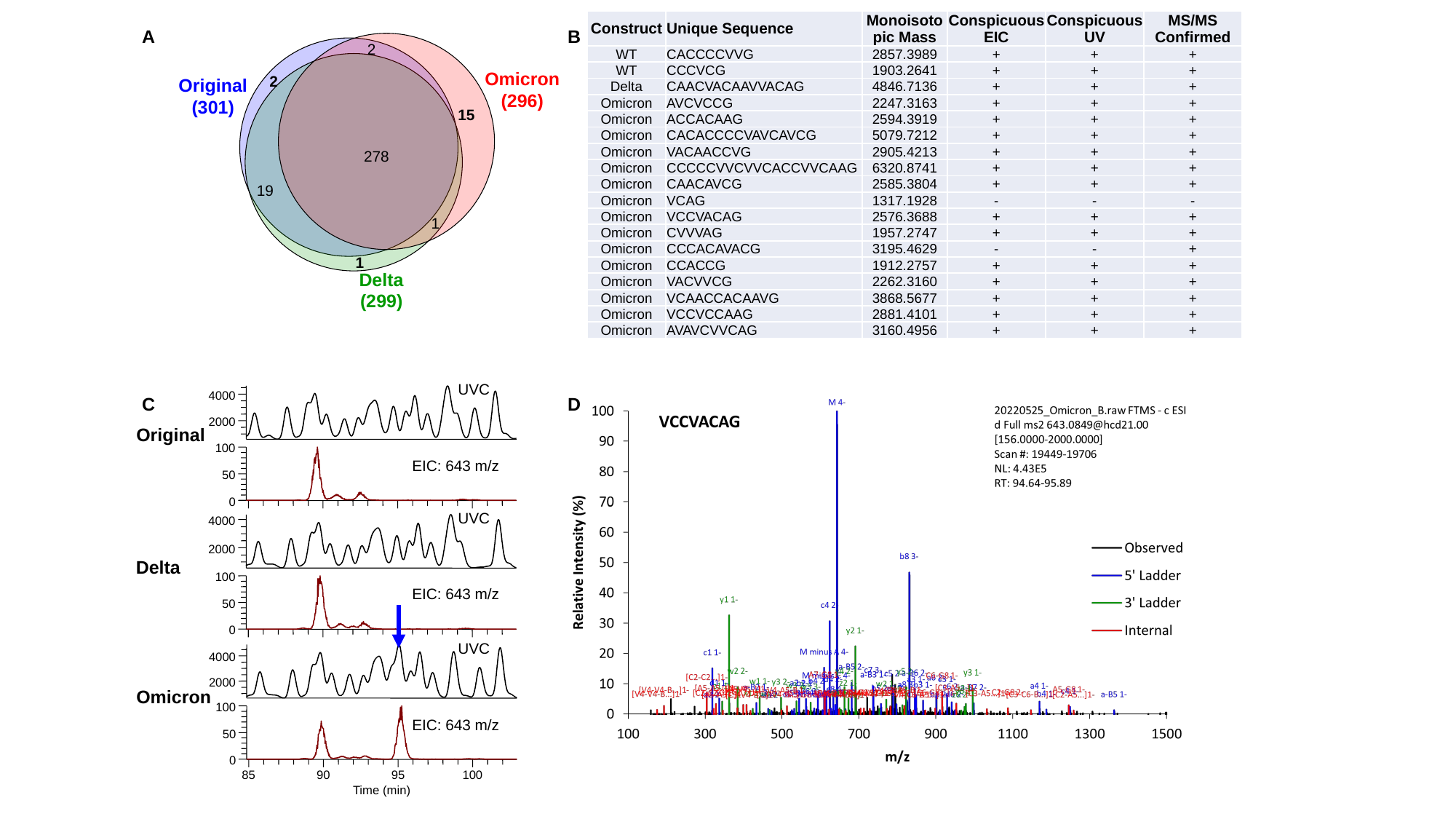

| Construct | Unique Sequence | Monoisotopic Mass | Conspicuous EIC | Conspicuous UV | MS/MS Confirmed |
| --- | --- | --- | --- | --- | --- |
| WT | CACCCCVVG | 2857.3989 | + | + | + |
| WT | CCCVCG | 1903.2641 | + | + | + |
| Delta | CAACVACAAVVACAG | 4846.7136 | + | + | + |
| Omicron | AVCVCCG | 2247.3163 | + | + | + |
| Omicron | ACCACAAG | 2594.3919 | + | + | + |
| Omicron | CACACCCCVAVCAVCG | 5079.7212 | + | + | + |
| Omicron | VACAACCVG | 2905.4213 | + | + | + |
| Omicron | CCCCCVVCVVCACCVVCAAG | 6320.8741 | + | + | + |
| Omicron | CAACAVCG | 2585.3804 | + | + | + |
| Omicron | VCAG | 1317.1928 | - | - | - |
| Omicron | VCCVACAG | 2576.3688 | + | + | + |
| Omicron | CVVVAG | 1957.2747 | + | + | + |
| Omicron | CCCACAVACG | 3195.4629 | - | - | + |
| Omicron | CCACCG | 1912.2757 | + | + | + |
| Omicron | VACVVCG | 2262.3160 | + | + | + |
| Omicron | VCAACCACAAVG | 3868.5677 | + | + | + |
| Omicron | VCCVCCAAG | 2881.4101 | + | + | + |
| Omicron | AVAVCVVCAG | 3160.4956 | + | + | + |
A
B
2
Omicron (296)
Original (301)
2
15
278
19
1
1
Delta (299)
UVC
4000
2000
100
50
0
4000
2000
100
50
0
4000
2000
100
50
0
85
90
95
100
Time (min)
Original
EIC: 643 m/z
UVC
Delta
EIC: 643 m/z
UVC
Omicron
EIC: 643 m/z
C
D

## Slide 23
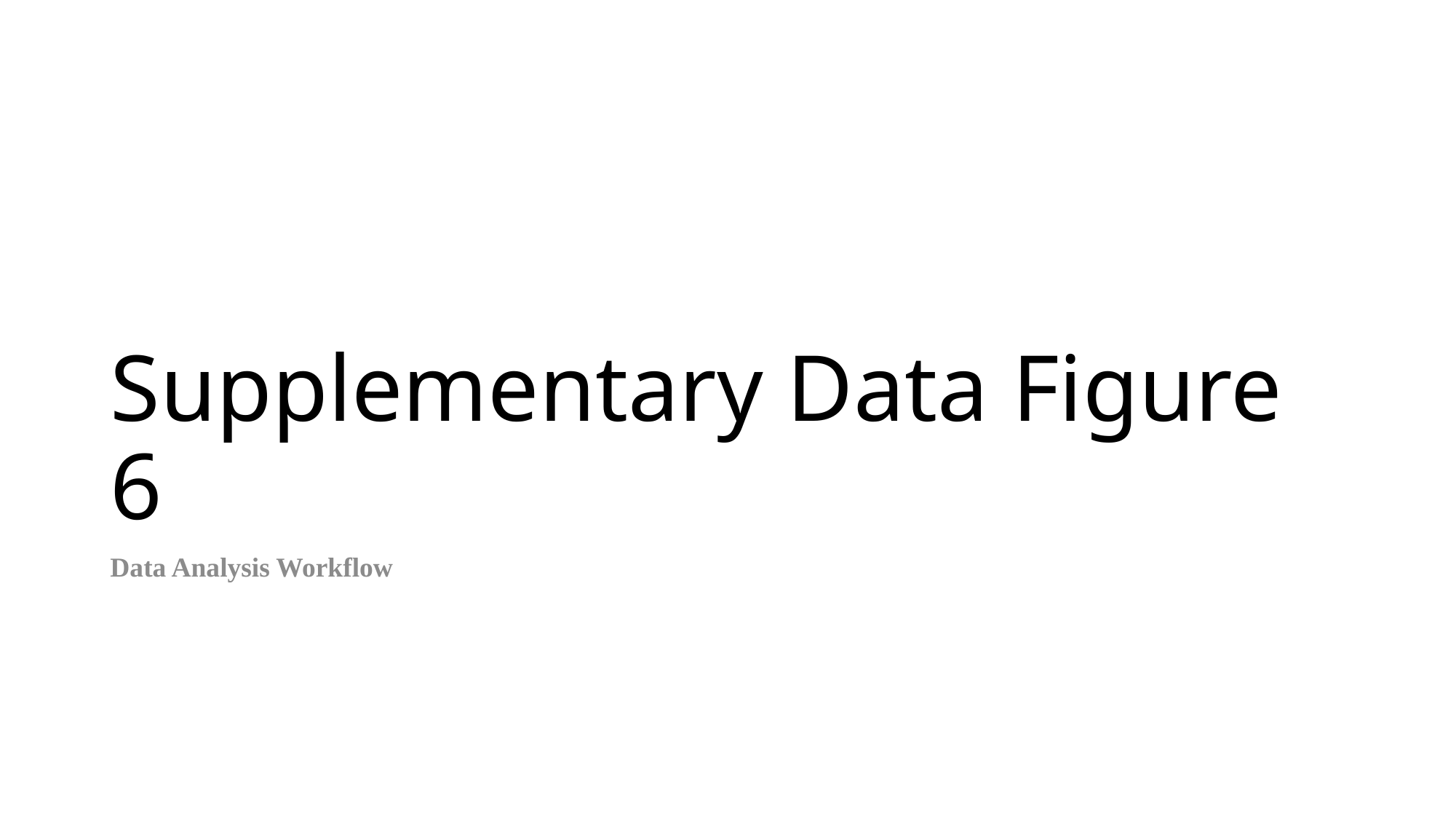

# Supplementary Data Figure 6
Data Analysis Workflow

## Slide 24
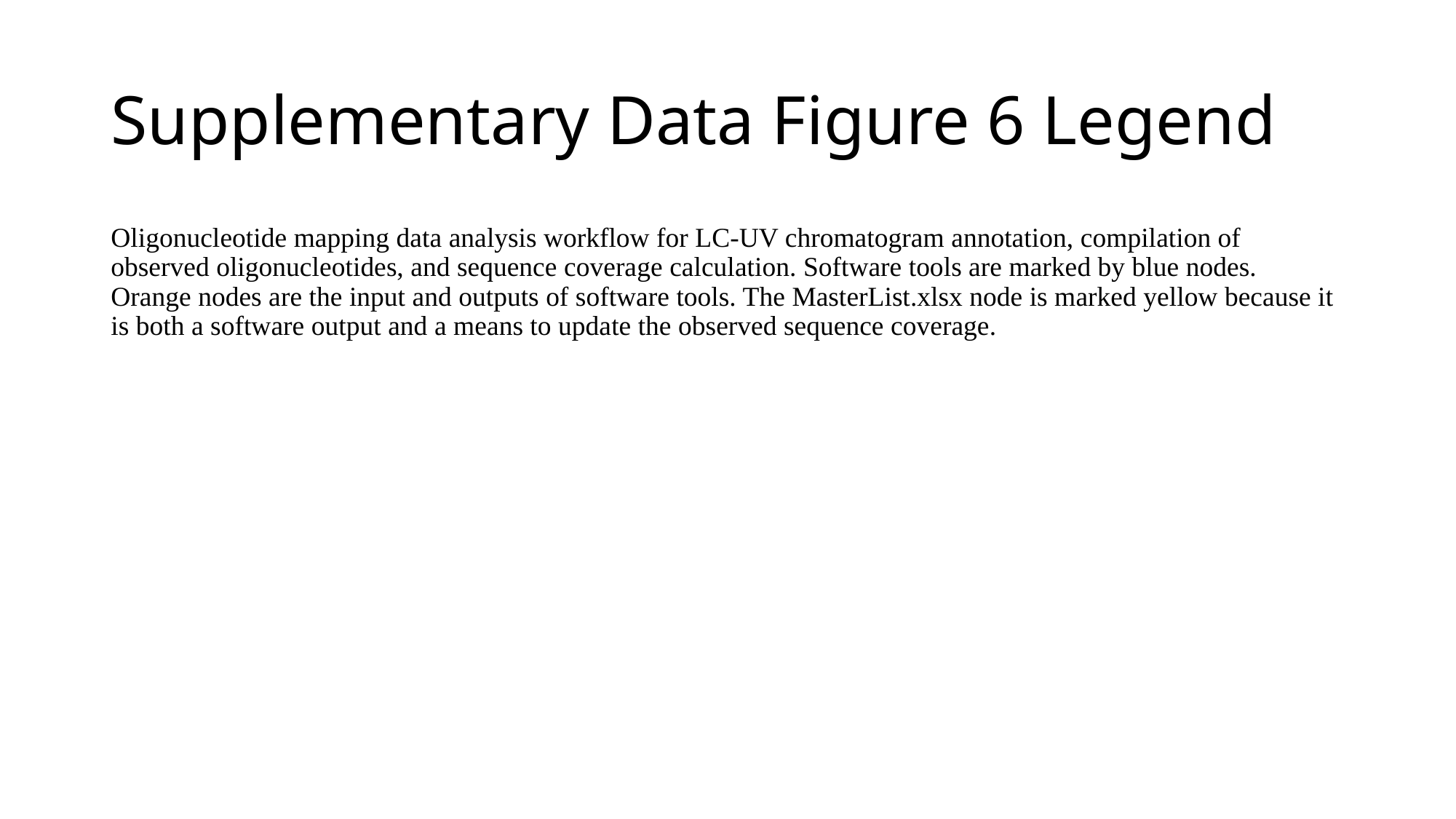

# Supplementary Data Figure 6 Legend
Oligonucleotide mapping data analysis workflow for LC-UV chromatogram annotation, compilation of observed oligonucleotides, and sequence coverage calculation. Software tools are marked by blue nodes. Orange nodes are the input and outputs of software tools. The MasterList.xlsx node is marked yellow because it is both a software output and a means to update the observed sequence coverage.

## Slide 25
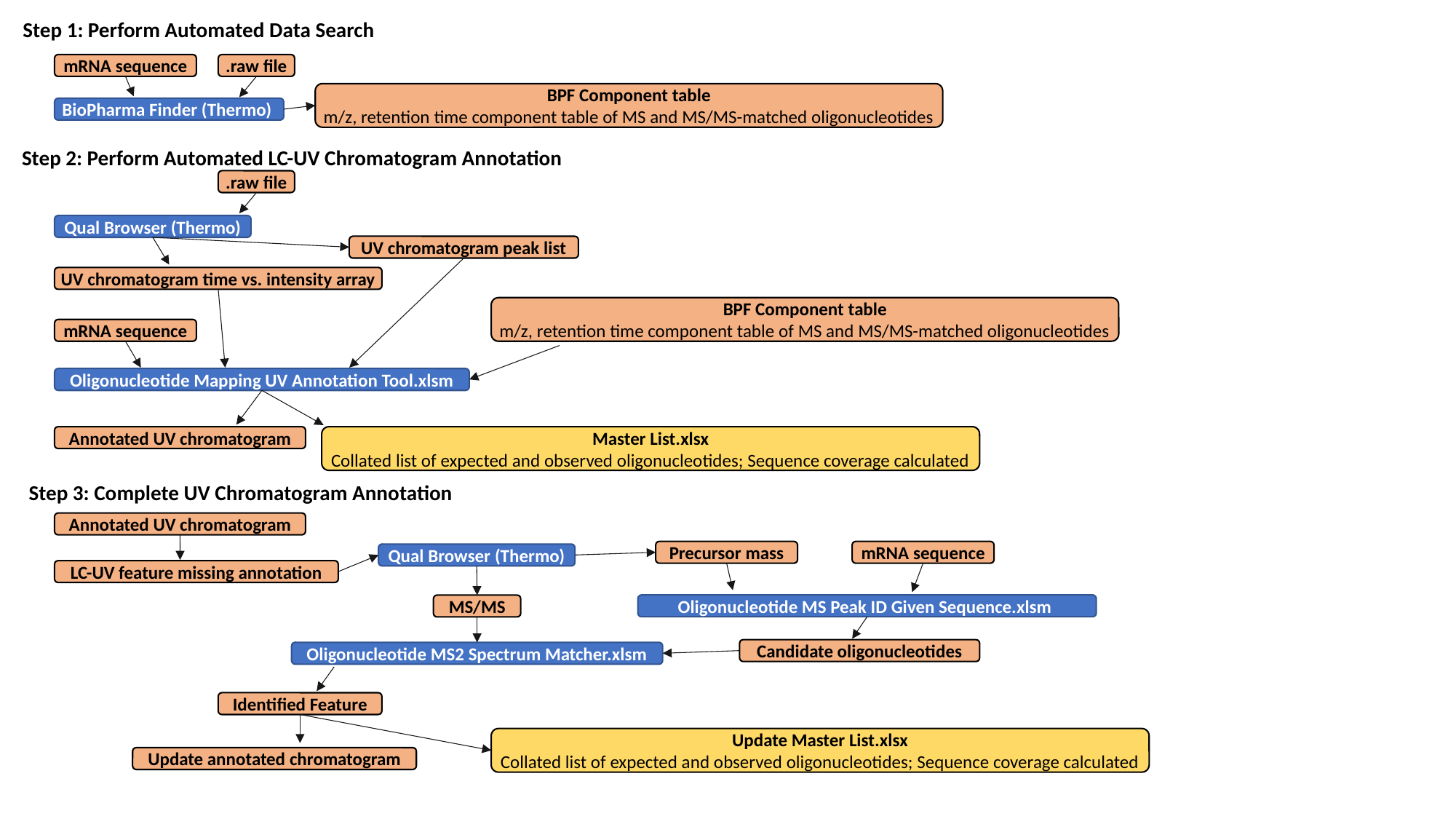

Step 1: Perform Automated Data Search
mRNA sequence
.raw file
BPF Component table
m/z, retention time component table of MS and MS/MS-matched oligonucleotides
BioPharma Finder (Thermo)
Step 2: Perform Automated LC-UV Chromatogram Annotation
.raw file
Qual Browser (Thermo)
UV chromatogram peak list
UV chromatogram time vs. intensity array
BPF Component table
m/z, retention time component table of MS and MS/MS-matched oligonucleotides
mRNA sequence
Oligonucleotide Mapping UV Annotation Tool.xlsm
Annotated UV chromatogram
Master List.xlsx
Collated list of expected and observed oligonucleotides; Sequence coverage calculated
Step 3: Complete UV Chromatogram Annotation
Annotated UV chromatogram
Precursor mass
mRNA sequence
Qual Browser (Thermo)
LC-UV feature missing annotation
Oligonucleotide MS Peak ID Given Sequence.xlsm
MS/MS
Candidate oligonucleotides
Oligonucleotide MS2 Spectrum Matcher.xlsm
Identified Feature
Update Master List.xlsx
Collated list of expected and observed oligonucleotides; Sequence coverage calculated
Update annotated chromatogram

## Slide 26
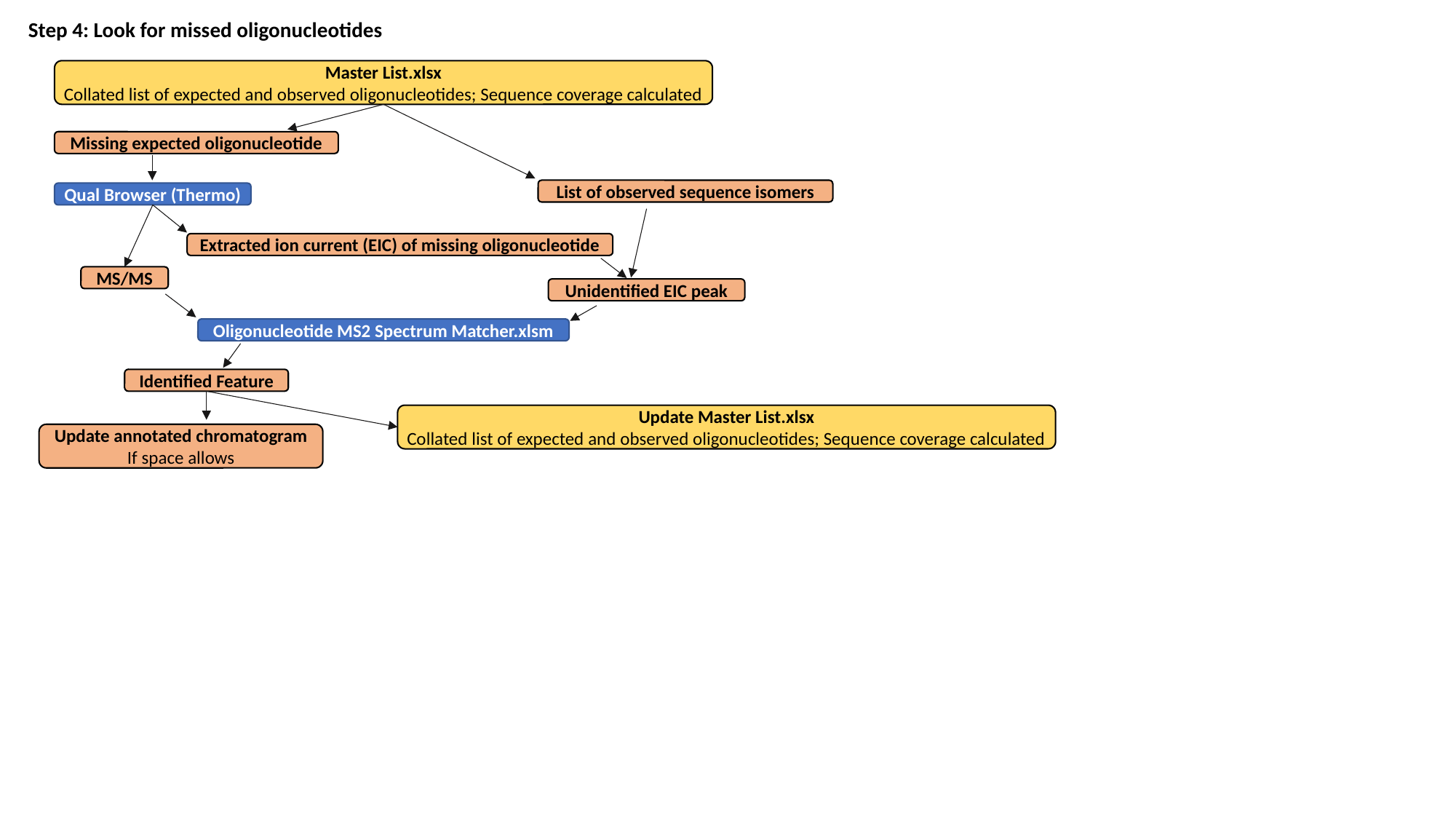

Step 4: Look for missed oligonucleotides
Master List.xlsx
Collated list of expected and observed oligonucleotides; Sequence coverage calculated
Missing expected oligonucleotide
List of observed sequence isomers
Qual Browser (Thermo)
Extracted ion current (EIC) of missing oligonucleotide
MS/MS
Unidentified EIC peak
Oligonucleotide MS2 Spectrum Matcher.xlsm
Identified Feature
Update Master List.xlsx
Collated list of expected and observed oligonucleotides; Sequence coverage calculated
Update annotated chromatogram
If space allows

## Slide 27
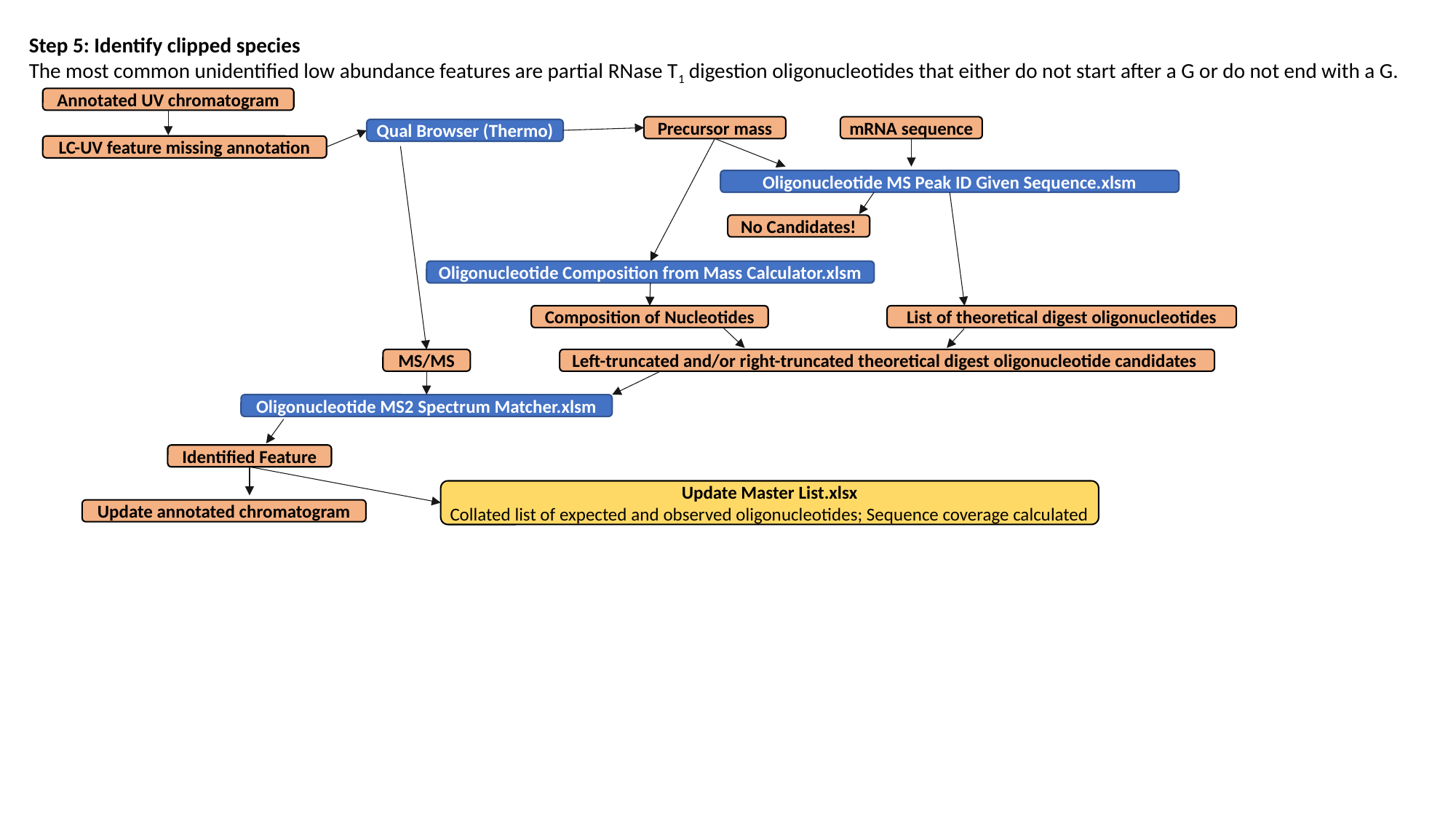

Step 5: Identify clipped species
The most common unidentified low abundance features are partial RNase T1 digestion oligonucleotides that either do not start after a G or do not end with a G.
Annotated UV chromatogram
Precursor mass
mRNA sequence
Qual Browser (Thermo)
LC-UV feature missing annotation
Oligonucleotide MS Peak ID Given Sequence.xlsm
No Candidates!
Oligonucleotide Composition from Mass Calculator.xlsm
Composition of Nucleotides
List of theoretical digest oligonucleotides
MS/MS
Left-truncated and/or right-truncated theoretical digest oligonucleotide candidates
Oligonucleotide MS2 Spectrum Matcher.xlsm
Identified Feature
Update Master List.xlsx
Collated list of expected and observed oligonucleotides; Sequence coverage calculated
Update annotated chromatogram

## Slide 28
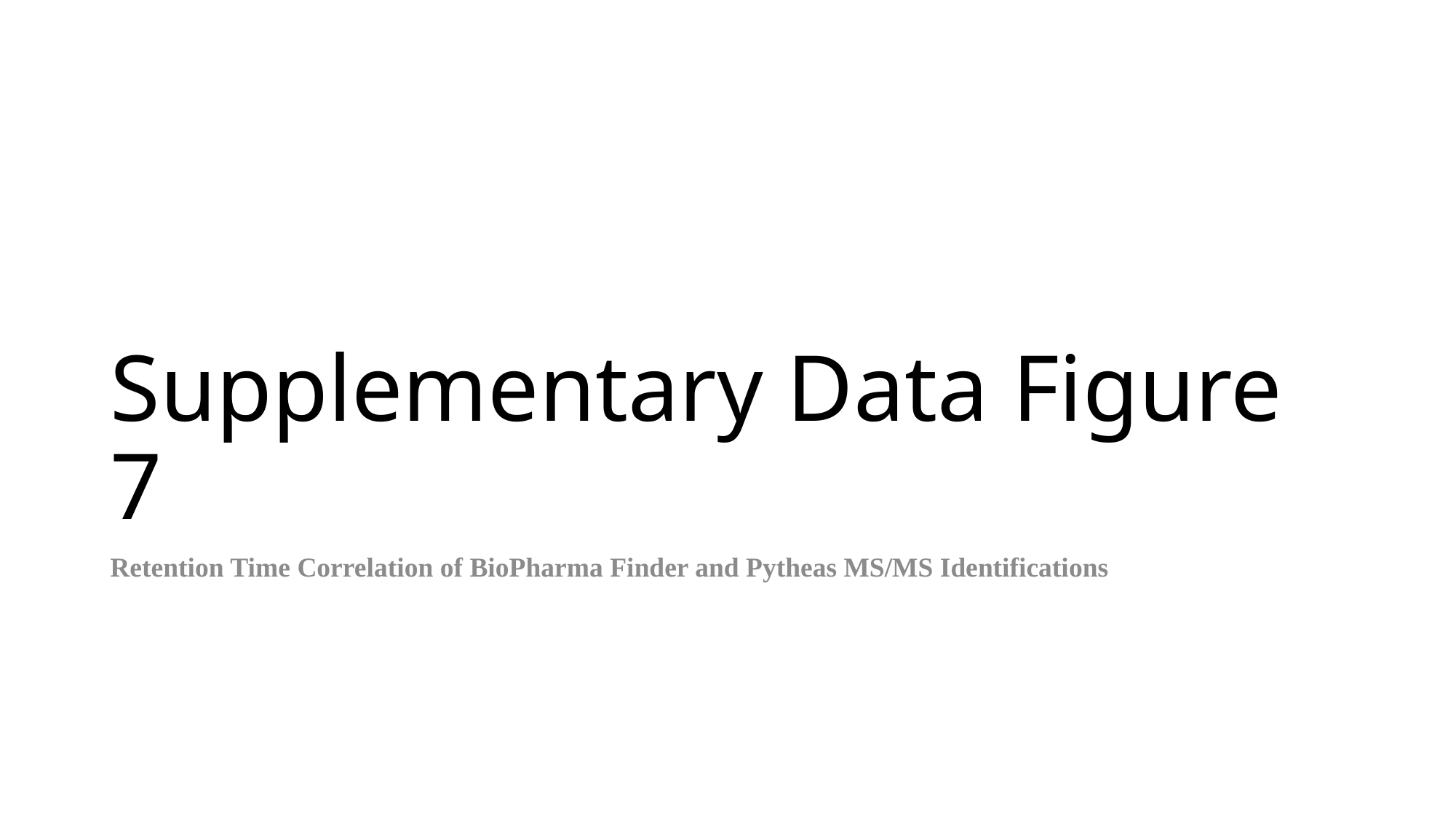

# Supplementary Data Figure 7
Retention Time Correlation of BioPharma Finder and Pytheas MS/MS Identifications

## Slide 29
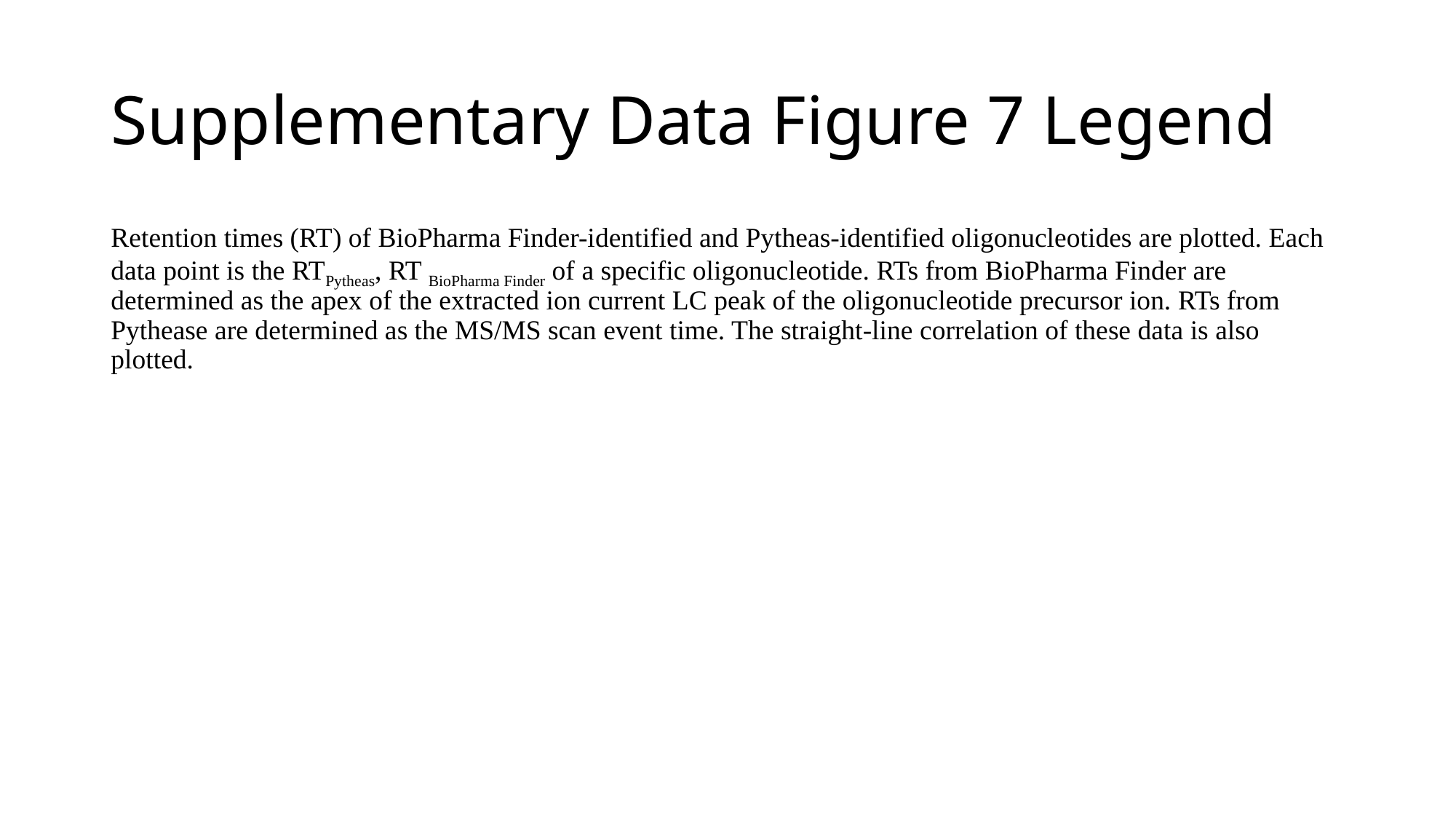

# Supplementary Data Figure 7 Legend
Retention times (RT) of BioPharma Finder-identified and Pytheas-identified oligonucleotides are plotted. Each data point is the RTPytheas, RT BioPharma Finder of a specific oligonucleotide. RTs from BioPharma Finder are determined as the apex of the extracted ion current LC peak of the oligonucleotide precursor ion. RTs from Pythease are determined as the MS/MS scan event time. The straight-line correlation of these data is also plotted.

## Slide 30
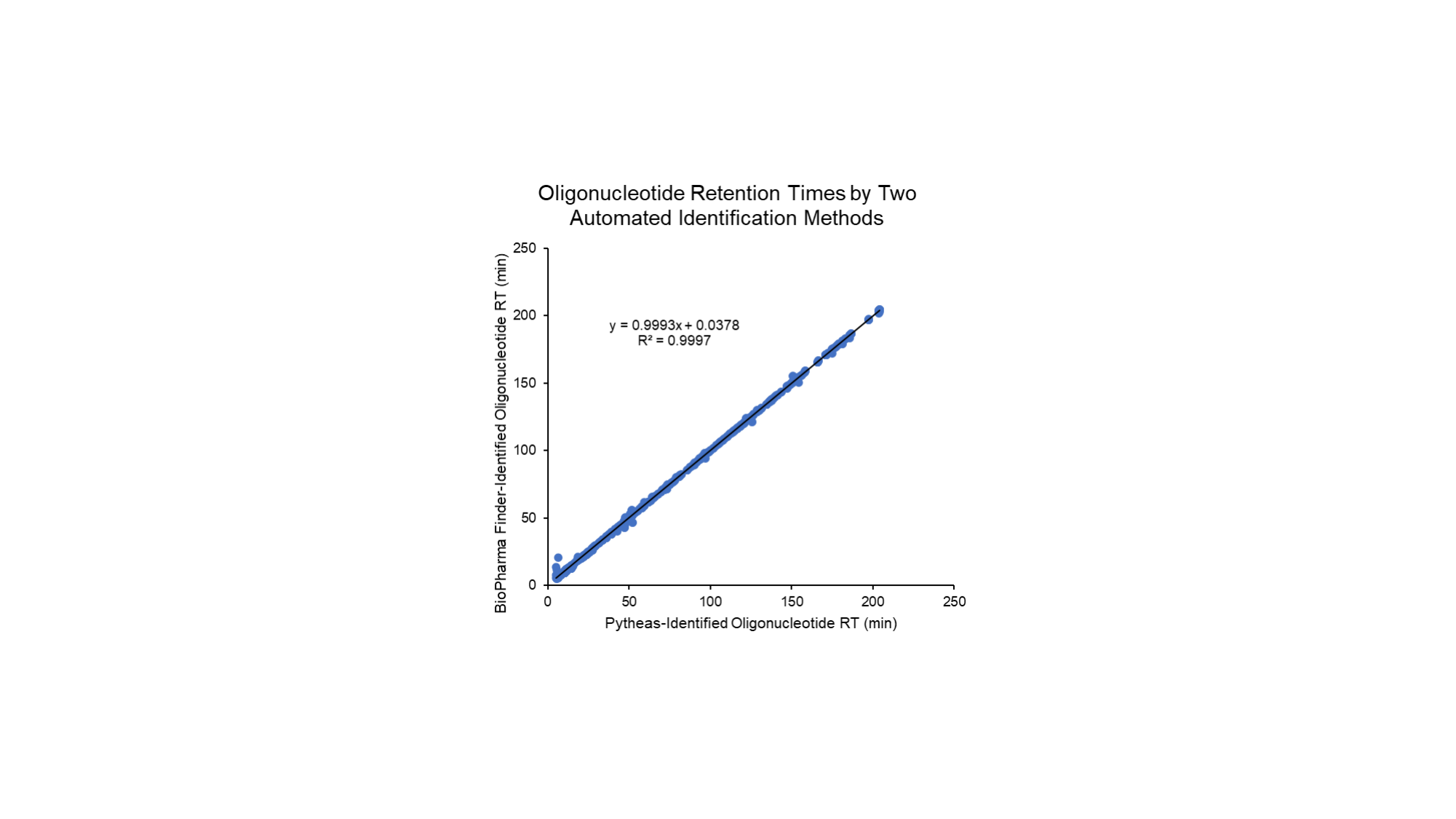

## Slide 31
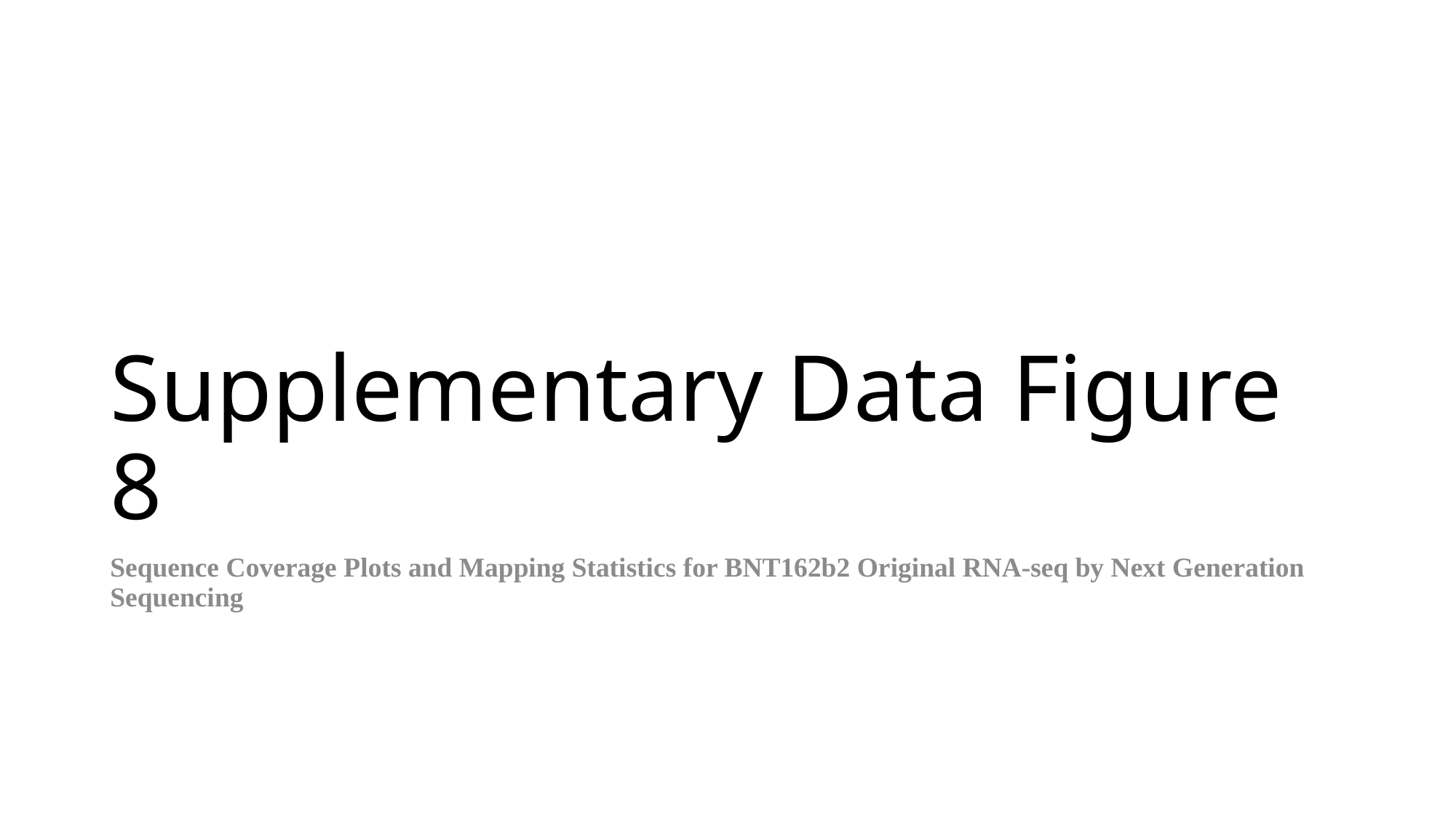

# Supplementary Data Figure 8
Sequence Coverage Plots and Mapping Statistics for BNT162b2 Original RNA-seq by Next Generation Sequencing

## Slide 32
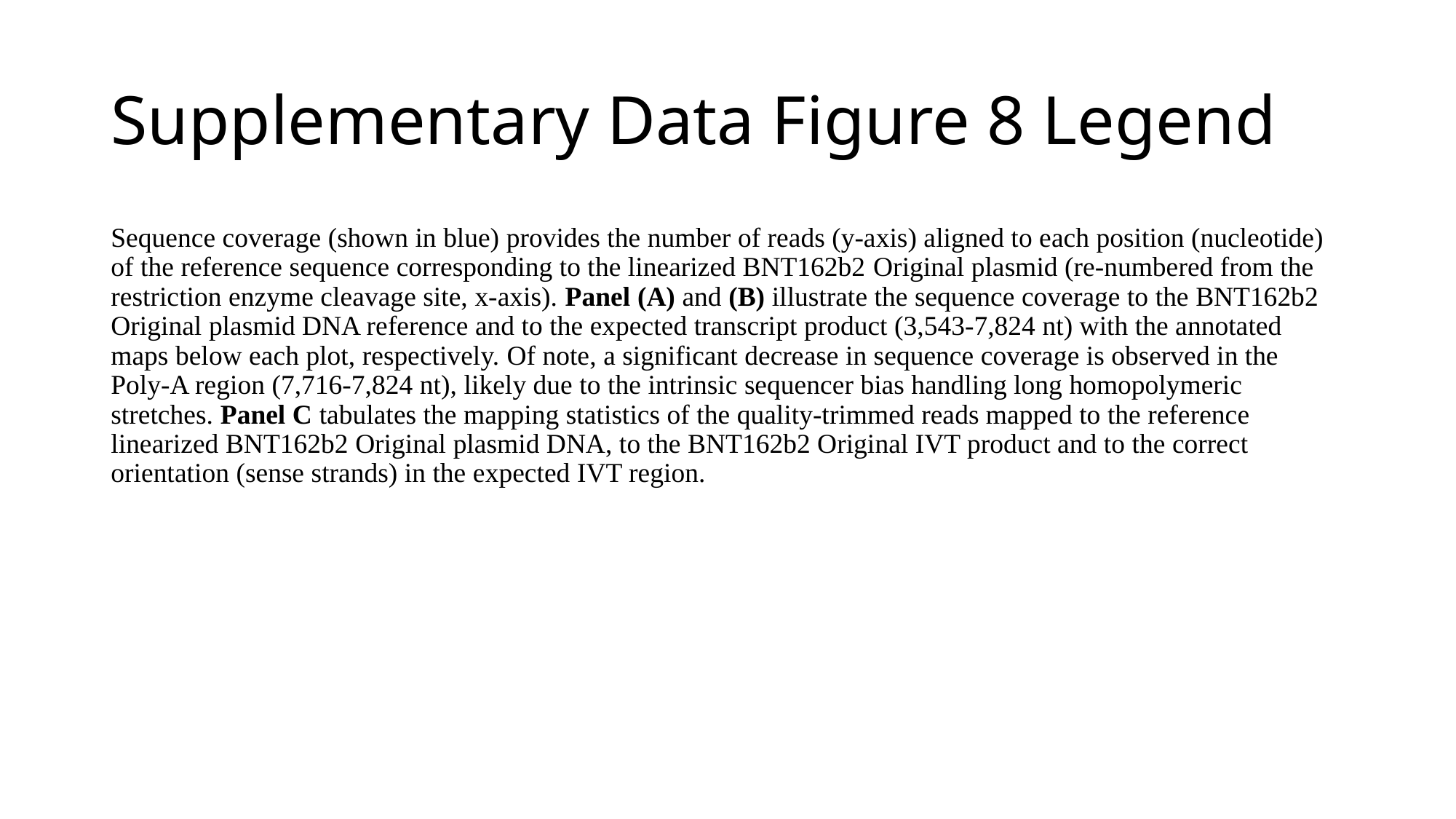

# Supplementary Data Figure 8 Legend
Sequence coverage (shown in blue) provides the number of reads (y-axis) aligned to each position (nucleotide) of the reference sequence corresponding to the linearized BNT162b2 Original plasmid (re-numbered from the restriction enzyme cleavage site, x-axis). Panel (A) and (B) illustrate the sequence coverage to the BNT162b2 Original plasmid DNA reference and to the expected transcript product (3,543-7,824 nt) with the annotated maps below each plot, respectively. Of note, a significant decrease in sequence coverage is observed in the Poly-A region (7,716-7,824 nt), likely due to the intrinsic sequencer bias handling long homopolymeric stretches. Panel C tabulates the mapping statistics of the quality-trimmed reads mapped to the reference linearized BNT162b2 Original plasmid DNA, to the BNT162b2 Original IVT product and to the correct orientation (sense strands) in the expected IVT region.

## Slide 33
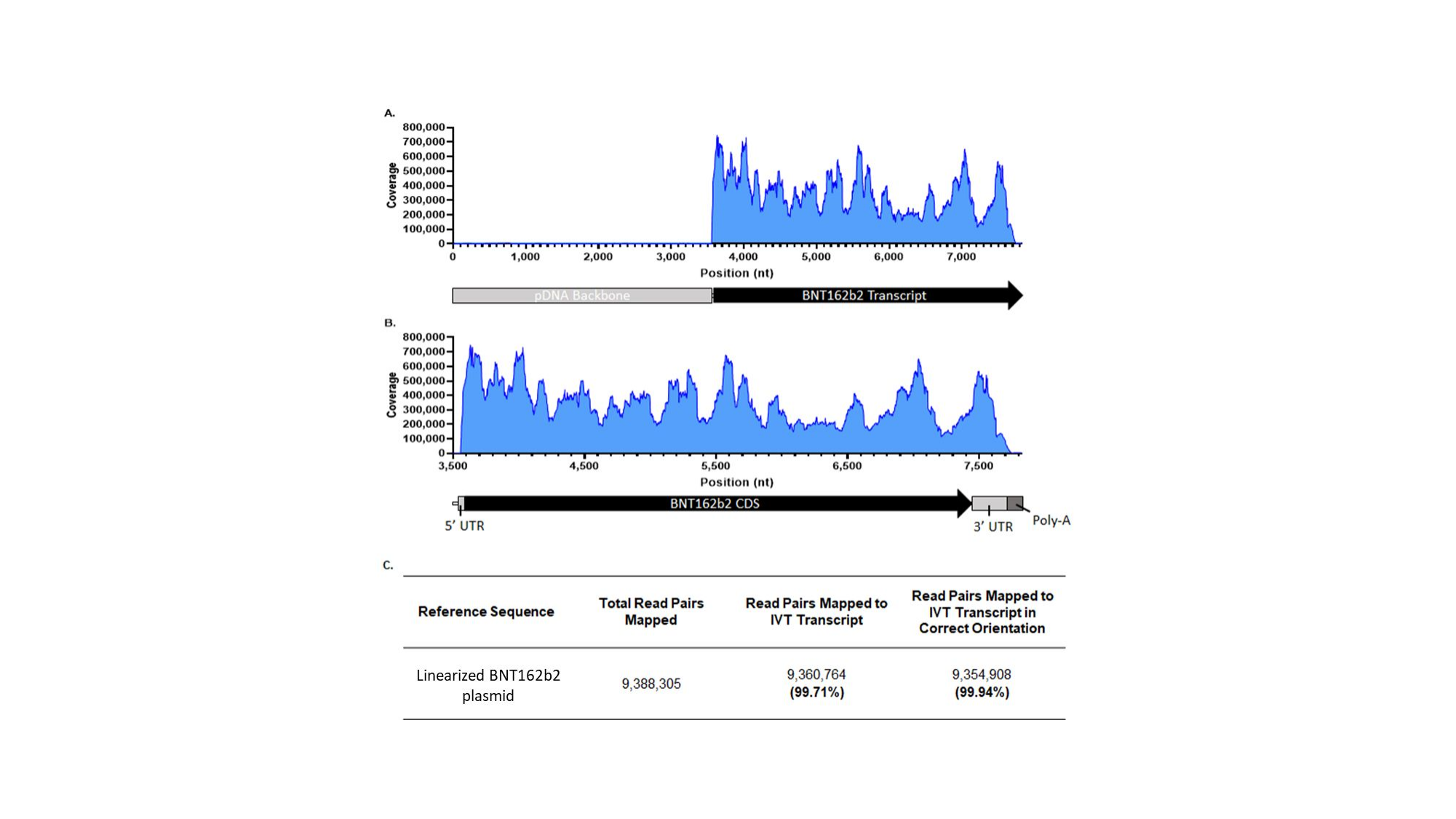

Supplement: Supplementary file 3 — Supplementary Figures. [file 41598_2023_36193_MOESM3_ESM.pptx]
